# Exploring African Medicinal Plants for Potential Anti-Diabetic Compounds with the DIA-DB Inverse Virtual Screening Web Server

**DOI:** 10.3390/molecules24102002

**Published:** 2019-05-24

**Authors:** Andreia S.P. Pereira, Helena den Haan, Jorge Peña-García, Marién M. Moreno, Horacio Pérez-Sánchez, Zeno Apostolides

**Affiliations:** 1 Department of Biochemistry, Genetics and Microbiology, University of Pretoria, Pretoria, Hillcrest 0083, South Africa; aspdpereira@gmail.com; 2 Structural Bioinformatics and High Performance Computing Research Group (BIO-HPC), Universidad Católica de Murcia, 30107 Murcia, Spain; hden@alu.ucam.edu (H.d.H.); Jorge.dlpg@gmail.com (J.P.-G.); memoreno@ucam.edu (M.M.M.)

**Keywords:** diabetes, anti-diabetic, DIA-DB, medicinal plants, *in silico*, virtual screening

## Abstract

Medicinal plants containing complex mixtures of several compounds with various potential beneficial biological effects are attractive treatment interventions for a complex multi-faceted disease like diabetes. In this study, compounds identified from African medicinal plants were evaluated for their potential anti-diabetic activity. A total of 867 compounds identified from over 300 medicinal plants were screened *in silico* with the DIA-DB web server (http://bio-hpc.eu/software/dia-db/) against 17 known anti-diabetic drug targets. Four hundred and thirty compounds were identified as potential inhibitors, with 184 plants being identified as the sources of these compounds. The plants *Argemone ochroleuca*, *Clivia miniata*, *Crinum bulbispermum*, *Danais fragans*, *Dioscorea dregeana*, *Dodonaea angustifolia*, *Eucomis autumnalis*, *Gnidia kraussiana*, *Melianthus comosus*, *Mondia whitei*, *Pelargonium sidoides*, *Typha capensis*, *Vinca minor*, *Voacanga africana*, and *Xysmalobium undulatum* were identified as new sources rich in compounds with a potential anti-diabetic activity. The major targets identified for the natural compounds were aldose reductase, hydroxysteroid 11-beta dehydrogenase 1, dipeptidyl peptidase 4, and peroxisome proliferator-activated receptor delta. More than 30% of the compounds had five or more potential targets. A hierarchical clustering analysis coupled with a maximum common substructure analysis revealed the importance of the flavonoid backbone for predicting potential activity against aldose reductase and hydroxysteroid 11-beta dehydrogenase 1. Filtering with physiochemical and the absorption, distribution, metabolism, excretion and toxicity (ADMET) descriptors identified 28 compounds with favorable ADMET properties. The six compounds—crotofoline A, erythraline, henningsiine, nauclefidine, vinburnine, and voaphylline—were identified as novel potential multi-targeted anti-diabetic compounds, with favorable ADMET properties for further drug development.

## 1. Introduction

According to the World Health Organization, in 2016, diabetes was the seventh leading cause of death, with an estimated 1.6 million people having died from the disease [1]. Diabetes is a chronic disease arising from impaired insulin secretion and insulin resistance, leading to its defining feature of hyperglycemia [2]. It is a multi-organ disease affecting the pancreas, liver, muscles, kidney, and central nervous system, and several complications such as hypertension, stroke, blindness, and kidney disease are associated with diabetes [2,3]. The main type of treatment for diabetes and controlling the associated hyperglycemia is in the form of insulin that primarily focuses on lowering and maintaining blood glucose levels [2]. However, in more recent years, as diabetes is a multifaceted disease, there has been an increase in the development of specific enzyme-targeted drugs, and specific inhibitors for targets like alpha-glucosidase, dipeptidyl peptidase-4 (DPP4), glucagon-like peptide-1 (GLP-1) receptor, and sodium-glucose co-transporter-2 (SGLT2) have been approved [3]. Unfortunately, some of these approved drugs have been met with some adverse effects [3]. As a better understanding of the pathogenesis and complexity in treating the disease arises, so too does the need for the development of more effective and safer drugs to treat the disease.

Throughout history, plants have played an important role in medicinal drug discovery as rich sources of unique and novel compounds for drug development. In several cultures, there is widespread traditional use of decoctions prepared from medicinal plants in the treatment of diabetes [4,5,6,7,8]. The use of decoctions prepared from medicinal plants in the treatment of a complex multi-faceted disease like diabetes is attractive, as they often contain more than one compound with various beneficial biological effects, thus potentially creating an effective and affordable multi-targeted treatment strategy [9,10]. In some cases, extensive scientific evaluations have been conducted on some of these traditional medicinal plants to validate their use in the treatment of diabetes, however, for the majority, there is a lack of scientific knowledge. 

*In silico* virtual screening methodologies are ideal for initial exploratory evaluations of the potential anti-diabetic activity of traditional medicinal plants. As plants are complex mixtures of several different compounds, with *in silico* virtual screening methods, hundreds of compounds can be screened against multiple diabetes targets rapidly and cost effectively. This strategy has been employed to identify anti-cancer, anti-stroke, and anti-Alzheimer’s compounds from traditional Chinese medicines, as well as their potential mechanisms of action [11,12,13]. In this study, we have implemented similar *in silico* methodologies to identify novel African medicinal plants as rich sources of compounds with potential anti-diabetic activity. 

## 2. Results and Discussion

### 2.1. Inverse Virtual Screening and Identification of Compounds with Potential Anti-Diabetic Activity

In this study, the anti-diabetic potential of natural compounds from African medicinal plants was explored with the DIA-DB web server (http://bio-hpc.eu/software/dia-db/) [14]. A total of 867 compounds were screened *in silico* against 17 diabetes targets. The ligands found crystallized with each protein target were also screened to decide a cutoff docking score, so as to distinguish between potential active and inactive compounds. The docking scores of the crystallized ligands ranged from −11.3 to −5.7 kcal/mol, and in some cases, the test compounds had better docking scores than the docking scores for the crystallized ligands (Table 1). A docking cutoff score of −9 kcal/mol was set, as it was deemed a reasonable average docking score that covered the top 10%–20% of the test compounds for each protein target [11,12,13]. 

**Figure 1:**
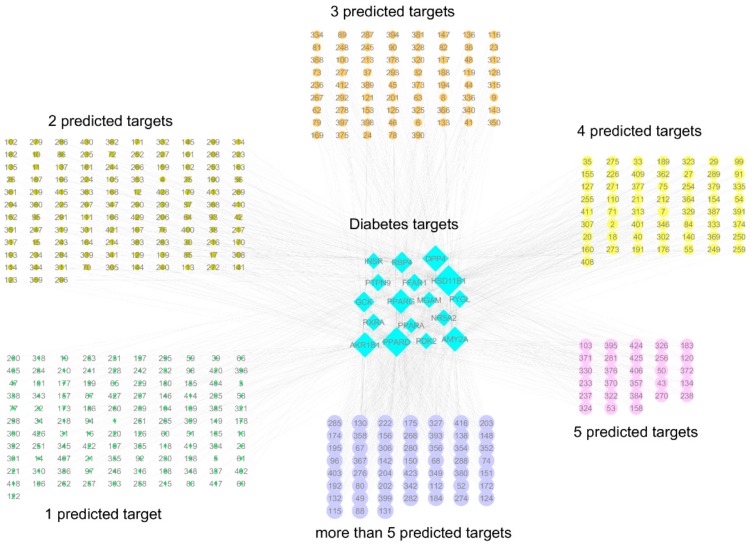
The network of compounds identified by virtual screening with the DIA-DB web server and their predicted targets. All of the predicted active compounds are represented by a number that corresponds to those given to the compounds in Table S2. The size of the target node depicts the number of predicted compounds, while the size of the compound node depicts the number of predicted targets. For the individual compound–target networks, please refer to Figure S1.

Of the 867 test compounds, a total of 430 were predicted as potentially active compounds, and the majority of these compounds were not limited to a single protein target only, with 30% of the predicted active compounds having five or more protein targets (Figure 1 and Table S2). Hydroxysteroid 11-beta dehydrogenase 1 (HSD11B1), peroxisome proliferator-activated receptor delta (PPARD), and DPP4 had the most predicted active compounds, with 208, 190, and 149, respectively, while protein targets peroxisome proliferator-activated receptor alpha (PPARA), insulin receptor (INSR), and intestinal maltase-glucoamylase (MGAM) had the least, with 6, 18, and 18, respectively (Figure S1). The difference in the number of predicted active compounds likely reflects the differences in the nature of the binding pockets of the target proteins, with some having large binding cavities that can accommodate different types and sizes of scaffolds. 

**Figure 2:**
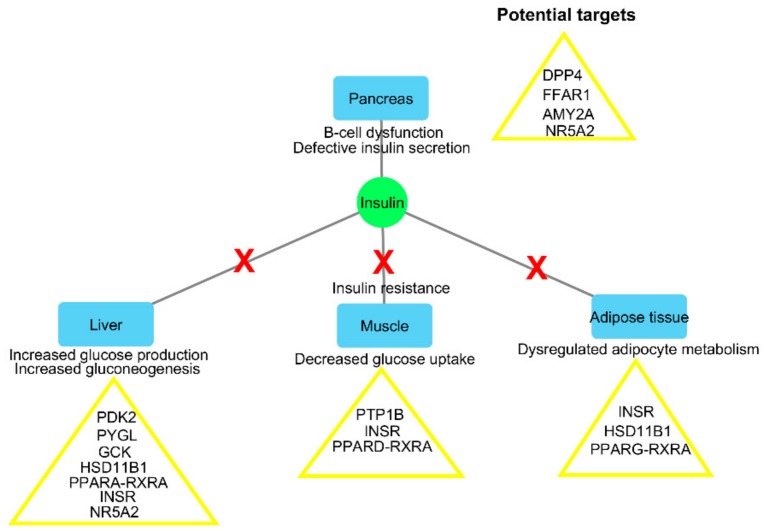
A simplified overview of some of the organ systems and their dysregulation involved in diabetes pathogenesis (adapted from Moller, 2001 [26]; Defronzo et al., 2014 [2]). Potential targets identified in each organ system relate to some of the virtual screening targets of the DIA-DB web server. Dipeptidyl peptidase-4 (DPP4); free fatty acid receptor 1 (FFAR1); glucokinase (GCK); hydroxysteroid 11-beta dehydrogenase 1 (HSD11B1); insulin receptor (INSR); liver glycogen phosphorylase (PYGL); liver receptor homolog-1 (NR5A2); pancreatic alpha-amylase (AMY2A); peroxisome proliferator-activated receptor alpha (PPARA); peroxisome proliferator-activated receptor delta (PPARD); peroxisome proliferator-activated receptor gamma (PPARG); protein tyrosine phosphatase (PTP); pyruvate dehydrogenase kinase isoform 2 (PDK2); retinoid X receptor alpha (RXRA).

The significance of the potential for multi-targeted compounds becomes apparent when one looks at the complexity of the diabetes disease pathogenesis. Diabetes is not the result of the dysregulation of a single target and/or pathway, but rather the dysregulation of multiple processes such as glucose and lipid metabolism, as well as insulin signaling in several organ systems, such as the pancreas, liver, muscles, and adipose tissue, leading to the hallmark of hyperglycemia [2,26] (Figure 2). Compounds capable of regulating one or more of the protein targets associated with these dysregulated processes across the different organ systems may be more effective in managing the disease than a “single target single drug” approach [27,28,29]. Not surprising, several manuscripts can be found in the literature on the potential use of combination drug therapy for the treatment of diabetes, including the combination of drugs with medicinal plants or herbs [9,10,30].

A total of 184 plants were identified as sources for the predicted active compounds (Table 2 and Table S3). Some plants were found to contain several predicted active compounds, while with others only one compound was identified. A limitation to this study was that one could not look at plants specifically enriched for anti-diabetic compounds, as not all of the compounds for all of the plants were listed in the books, and in several cases, only one major compound was listed for a plant. Following extensive literature searches for previous literature on the anti-diabetic potential of the plants and the compounds, it was found that of the 430 predicted active compounds, 125 had previous literature on their anti-diabetic potential, leaving a total of 305 newly identified potential anti-diabetic compounds.

From the plants, 82 plants were found with previous literature (namely traditional and experimental evidence) (Table S3); 12 plants were identified with traditional use for diabetes (Table S3), but no experimental evidence to date; and 90 plants were identified as new potential sources of anti-diabetic compounds (Table 2). Of particular interest was that the majority of these 90 plants were sourced from *Poisonous Plants of South Africa* [31], indicating the potential for toxicity of the compounds.

More than 60% of the plants with previous experimental literature on their anti-diabetic activity were found to contain one or more compound/s that were also found to have previous literature on their anti-diabetic potential. This suggests that these compounds are likely responsible for the observed experimental activity of the medicinal plant. This is true in the case of several plants, such as Aspalathus linearis and compounds aspalathin, isoorientin, orientin, and quercetin [32,33,34]; Cryptolepis sanguinolenta and compound cryptolepine [35]; Garcinia kola and compounds garcinia biflavonoid 1 and 2 and kolaflavanone [36,37]; Glycyrrhiza glabra and compound glycyrrhizin [38]; Hoodia gordonii and compound P57 [39]; Ligustrum lucidum and compound oleanolic acid [40]; Moringa oleifera and compounds kaempferol and quercetin [41]; Olea europaea and compounds oleuropein and oleanolic acid [42]; Punica granatum and compounds punicalin and punicalagin [43]; Ruta graveolens and compound rutin [44]; Styphnolobium japonicum and compound sophoricoside [45]; Syzygium cordatum and compound oleanolic acid [46]; Vernonia amygdalina and compounds 1,5-dicaffeoylquinic acid, chlorogenic acid and luteolin-7-rutinoside [47]; and Withania somnifera and compound withaferin A [48]. The identification of both plants and compounds with previous literature on their potential anti-diabetic activity provides some validation for the methodology used in this study.

**Figure 3:**
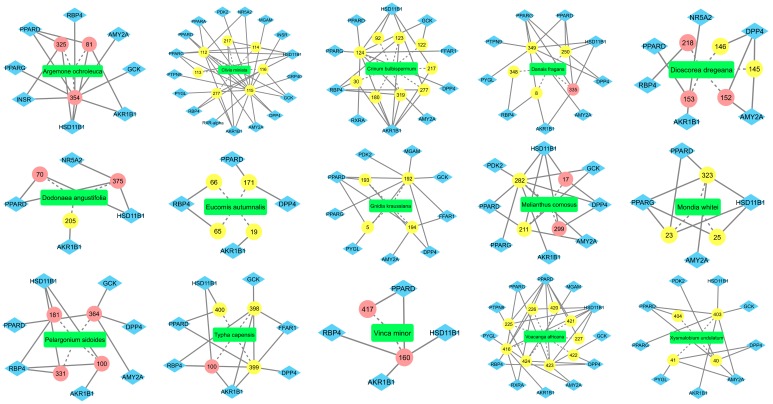
Fifteen plants identified as new sources rich in compounds with potential anti-diabetic activity for exploratory *in vitro* anti-diabetic studies. Compounds represented by their assigned numerical identity (Table S2); compounds represented by pink ellipses are compounds with previous literature on their anti-diabetic potential; compounds represented by yellow ellipses are novel compounds. Dashed edges represent the edges connecting the plant with its predicted bioactive compounds; solid edges represent the edges connecting the compounds with their predicted protein targets.

Of interest were the plants found containing compounds with previous literature on the compound’s potential anti-diabetic activity, but to date, the medicinal plant itself has not been evaluated for its potential antidiabetic activity. These plants were Argemone ochroleuca with compounds berberine [49], protopine [50] and sanguinarine [51]; Dioscorea dregeana with compounds dioscin [52,53], diosgenin [18,54] and hiricinol [55]; Dodonaea angustifolia with compounds beta-sitosterol [56] and stigmasterol [57,58]; Melianthus comosus with compounds 3-epioleanolic acid [59] and oleanolic acid [60]; Pelargonium sidoides with compounds catechin [61], gallocatechin [62,63], quercetin [64] and sitosterol-3-glucoside [65,66]; and Vinca minor with compounds eburnamonine and vincamine [67]. These plants represent a good initial point for exploratory *in vitro* anti-diabetic studies. These plants with their bioactive compounds and predicted targets are depicted in Figure 3.

Other plants of interest were those that had no previous literature, but contained several compounds (also with no previous literature) that were identified in this study as having a potential anti-diabetic activity. These plants were Mondia whitei and compounds 5-chloropropacin, 7-hydroxy-4,6-dimethoxypropacin and propacin; Voacanga Africana and its compounds ibogaine, ibogamine, iboxygaine, vinburnine, voacamine, voacangine, voacorine, voaphylline and vobtusine; and Xysmalobium undulatum and compounds allouzarin, alloxysmalorin, uzarigenin, uzarin (Figure 3). Of note, these three plants have been used traditionally to treat diabetes, but lack the accompanying scientific evidence [68,69,70]. The identification of the compounds found in these plants with a potential anti-diabetic activity provide some rationale for the traditional use of these plants in the treatment of diabetes. The plants Clivia miniata, Crinum bulbispermum, Danais fragans, Eucomis autumnalis, Gnidia kraussiana, and Typha capensis were also of interest, as these plants were found to contain four or more compounds that had been previously identified as having potential anti-diabetic activity (Figure 3).

### 2.2. Identification of Potentially Important Scaffolds for Enzyme Activity

A hierarchical clustering analysis of the compounds identified in each protein target group was performed using Tanimoto similarities to identify whether any compounds showed some similar molecular features [11,12,13] (Figure S2). From these clustering results, the maximum common substructure (MCS) analysis was performed in an attempt to identify any potential scaffolds important for predicting the potential activity within the largest cluster group identified (Table 3). No clustering of compounds was found for six of the protein target-compound groups, namely INSR, liver receptor homolog-1 (NR5A2), pyruvate dehydrogenase kinase isoform 2 (PDK2), PPARA, protein tyrosine phosphatase non-receptor type 9 (PTPN9), liver glycogen phosphorylase (PYGL), and retinoid X receptor alpha (RXRA). This is not surprising, as these protein target–compound groups were relatively small groups, with the number of predicted active compounds below 50—the two exceptions being the free fatty acid receptor 1 (FFAR1) and the MGAM protein-compound groups, which had only 37 and 18 predicted active compounds, respectively. Within the FFAR1 and MGAM groups, two clusters of similar compounds were evident that encompassed the majority of the compounds within the groups, namely 26 of 37 for FFAR1 and 12 of 18 for MGAM. Interestingly though, the MCS analysis produced only relatively small scaffold structures for the similar compounds within these groups, namely, a phenol group for FFAR1 and a methoxyphenol for MGAM. The importance of the benzene ring with a substituent group was also evident in the protein–compound groups of glucokinase (GCK), PPARD, peroxisome proliferator-activated receptor gamma (PPARG), and retinol binding protein 4 (RBP4).

The hierarchical clustering analysis of the HSD11B1 group revealed a total of 37 different clusters (Figure S2), with the largest cluster containing 40 similar compounds, and based on an MCS analysis, an important scaffold for HSD11B1 activity would be a flavonoid type of backbone. Interestingly, three compounds found in this group, namely apigenin, quercetin, and genistein, were recently shown to inhibit HSD11B1 [71]. Similarly, in the aldose reductase (AKR1B1) group, the centroid of the largest cluster found was calycosin, an isoflavone, and the MCS was a benzopyranol scaffold that can be found in the backbone of flavonoids. As with HSD11B1, there is literature on the inhibitory activity of AKR1B1 by flavonoids and their glycosides [72,73]. For the DPP4 and pancreatic alpha-amylase (AMY2A) groups, a more hydrophobic core scaffold with a hydrophilic head/tail was observed as the MCS for these two protein targets. The compounds found in the largest cluster of these groups had predominately triterpenoid or steroidal backbones with/without a glycosidic group attached, such as shown in the two centroid compounds maslinic acid and balanitin-6. The compounds corosolic acid, betulinic acid, glycyrrhizin, and sitosterol-3-glucoside with this type of backbone found in the AMY2A group, have been shown in previous literature to inhibit the enzyme [52,66,74].

### 2.3. Molecular Similarity Evaluation of Predicted Active Compounds and Known/Experimental Anti-Diabetic Drugs

**Figure 4:**
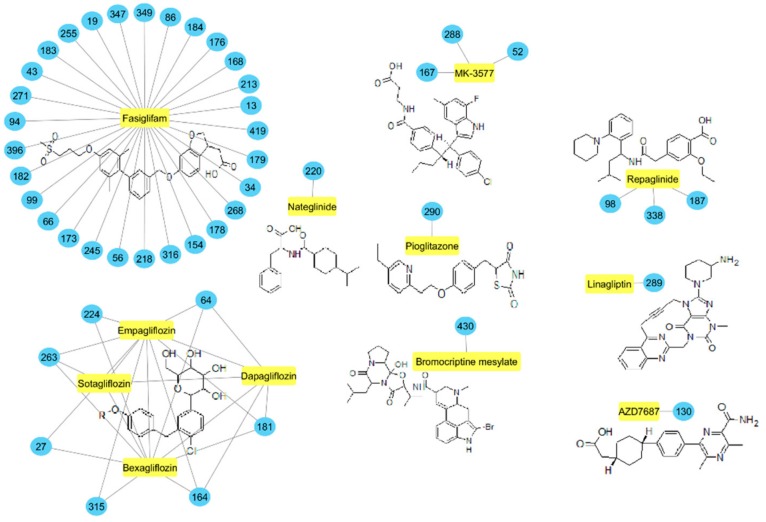
Molecular similarity analysis of predicted active compounds and some known/experimental anti-diabetic drugs. The similarity was performed on the extended connectivity fingerprint 4 (ECFP4) molecular fingerprints of compounds with a Tanimoto similarity cut-off score of 0.7.

A Tanimoto similarity analysis was performed to determine whether any similar molecular features occurred between the natural compounds and known/experimental anti-diabetic drugs [11,12,13]. As seen in Figure 4, only a small portion (approximately 10%) of the predicted active compounds showed some similarity with the known anti-diabetic drugs. Thus, for the most part, natural compounds from African medicinal plants present rather novel and unique scaffolds for anti-diabetic drug design. The majority of these compounds showed similar molecular features to fasiglifam (TAK-875), an experimental FFAR1 agonist [2].

Three of these compounds, namely, biochanin A (86), fujikinetin (176), and hesperitin (213), were also found by the DIA-DB web server as potential FFAR1 agonists; thus, these similarity studies with known drugs may further support their potential activity. Of interest was that seven of the predicted active compounds, namely, 8-hydroxypinoresinol (27), aspalathin (64), epicatehin (164), gallocatechin (181), hypoxoside (224), leucocyanidin (263), and pinoresinol (315), showed some structural similarity with the gliflozins bexagliflozin, dapagliflozin, empagliflozin, and sotagliflozin. The gliflozins are SGLT2 inhibitors [3]. Although SGLT2 was not included in the DIA-DB target screening panel, the similarity of these compounds with the known drugs may present SGLT2 as a novel anti-diabetic target for these seven compounds, and, of note, aspalathin has been found to be an inhibitor of SGLT2 [75]. Similarly, compounds carapanaubine (98), gelsemicine (187), and rauvoxinin (338), as well as hyoscyamine (220), showed some molecular similarity with repaglinide and nateglinide, respectively. Repaglinide and nateglinide are ATP-dependent potassium (K^+^) channel binders that stimulate the release of insulin from the pancreatic β-cells [76].

### 2.4. Prediction of Oral Bioavailability and Favourable Abosrption, Distribution, Metabolism, Excretion and Toxicity (ADMET) Properties of the Predicted Active Compounds

The oral bioavailability, as well as some ADMET parameters, were evaluated for each of the compounds. These are not only important parameters to evaluate for further drug development [11,12,13], but considering that in some areas where easy access to anti-diabetic medication is not always a possibility, an important way for patients to receive some form of anti-diabetic treatment would be through the use of a decoction from a medicinal plant having anti-diabetic properties. Therefore, factors such as the aqueous solubility and oral bioavailability of the bioactive compounds would be of great importance. Also, as several of the compounds investigated in this study were found in *Poisonous Plants of South Africa* [31], it is important to study the potential toxicity of these compounds.

The ADMET parameters for the predicted active compounds were compared to a group of 48 approved and experimental anti-diabetic drugs [2,3]. Also, a comparison of the ADMET parameters for the predicted active compounds with no previous literature (novel compounds) was compared to that of the predicted active compounds that had some previous literature on their potential anti-diabetic activity (known compounds). These known compounds would serve as another “positive control”.

**Figure 5:**
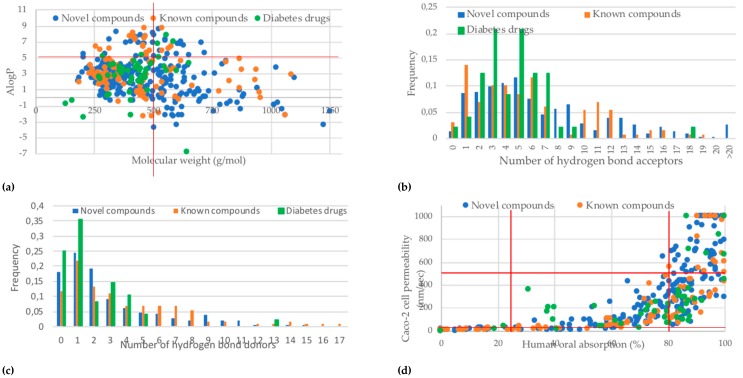
Prediction of druglikeness and bioavailability of hit compounds (novel and known) versus diabetic drugs (**a**) molecular weight versus AlogP, Lipinski’s rule of five, namely: compounds need to have a molecular weight of 500 g/mol or less and AlopP must be below 5; (**b**) frequency of hydrogen bond acceptors, Lipinski’s rule of five—not more than 10 hydrogen bond acceptors; (**c**) frequency of hydrogen bond donors, Lipinski’s rule of five—not more than 5 hydrogen bond donors; (**d**) QikProp prediction of percent human oral absorption versus Caco-2 cell permeability, percentage oral bioavailability below 25% is poor and above 80% is high, predicted cell permeability for non-active transport below 25 nm/s is poor, while above 500 nm/s is very good.

A summary of the Lipinski’s rule of five is depicted in Figure 5. As can be seen from Figure 5, a major violation of Lipinski’s rule of five was the molecular weight of the compounds, with 30% of the predicted active compounds violating this rule, namely, that the molecular weight must not exceed 500 g/mol (Figure 5a) [77]. This was also the major violation for the anti-diabetic drug control group. The number of hydrogen bond donors and acceptors for the majority of predicted active compounds was within the limitations (Figure 5b,c). No compound was found to violate all four rules, and 16% had three violations—only acarbose in the anti-diabetic drug control group had three violations. Nearly 50% of the predicted active compounds violated one or more of Lipinski’s rule of five, versus only 25% of the anti-diabetic drug control group. This is not surprising, as often such target-specific anti-diabetic drugs are designed taking these factors into consideration. It was also observed that the compounds predicted as having poor oral absorption were also predicted to have poor Caco-2 cell permeability and vice versa (Figure 5d).

A complete summary of all of the ADMET parameters evaluated for the compounds can be found in Table 4. The two major toxicity failures and points of concern for the predicted active compounds were immunotoxicity and blockage of the hERG K^+^ channels, with 75% of the compounds being predicted as potential immunotoxins, and 45% predicted as potential inhibitors of the hERG K+ channels. Interestingly, these two toxicity parameters were also the two major failures for the anti-diabetic drug control group. The model for the prediction of immunotoxicity is built on a training set of T- and B-cell growth inhibition data from the National Cancer Institute [78]. In some cases, it is likely that the predicted immunotoxicity may rather be a function of the compound concentration than a specific effect, and also, the model cannot distinguish immunosuppressive effects from immunomodulatory or immunostimulant effects. The predictive model for the human ether-a-go-go-related gene potassium (hERG K^+^) channel blockage is often used to predict the potential cardiac toxicity of the compounds [79]. It was expected that some of the compounds would be predicted as potential cardiac toxins, as some of the predicted active compounds are known cardiac glycosides, such as digitoxin, tyledoside C, bovoside, oleandrin, proscillaridin A, scillaren A, uzarin, and gomphoside [31,80].

After taking all of the ADMET parameters into account, only 28 of the predicted active compounds were found to have favorable ADMET properties, and these are shown in Table 5. These compounds present novel scaffolds with potential anti-diabetic activity and favorable ADMET properties for further drug design and development. Of these 28 compounds, eight have shown anti-diabetic properties in previous studies, and these were 2-hydroxygenistein [81], apigenin [82], catechin [61], cyanidin [83], eburnamonine [67], epicatechin [84], eriodictyol [85], and lapachol [86]. 

Ten of the compounds, namely, apigenin, catechin, crotofoline A, cyanidin, eburnamonine, erythraline, henningsiine, nauclefidine, vinburnine, and voaphylline were predicted as potential inhibitors of three or more anti-diabetic targets. AKR1B1, HSD11B1 PPARD, and RBP4 were the major targets identified for the 28 compounds. Also, of particular note, was that the plant *Voacanga africana* was found to contain three of these compounds with favorable ADMET properties, namely vinburnine, voaphylline, and withasomnine, and two of these compounds, vinburnine and voaphylline, were identified as potential multi-targeted compounds. These observations provide some evidence for the traditional use of *Voacanga africana* in the treatment of diabetes and further *in vitro* and *in vivo* studies are now needed to validate its use for diabetes.

## 3. Materials and Methods

### 3.1. Preparation of Compound Structures and Inverse Virtual Screening of Potential Anti-Diabetic Activity

The natural compounds were sourced from three books that catalogue the different medicinal plants found in Africa, as well as their medicinal uses and chemical constituents. These three books were *African Herbal Pharmacopoeia* [87], *Medicinal Plants of South Africa* [80], *and Poisonous Plants of South Africa* [31]. Where a graphical representation of the compound was given in the books, the two-dimensional structure of the compounds was created with Advanced Chemistry Development (ACD)/ChemSketch freeware version 12.02, 2010 [88], and then converted to its representative simplified molecular-input line-entry system (SMILES) notation. Where only the name of the compound was given, the two-dimensional structure and SMILES notation was obtained from PubChem [89]. The SMILES notations for the compounds analyzed in this study can be found in Table S1.

The SMILES notation of each compound was subsequently submitted to the DIA-DB web server that employs inverse virtual screening of compounds with Autodock Vina against a given set of 17 protein targets associated with diabetes [14]. These targets were AKR1B1, DPP4, FFAR1, GCK, HSD11B1, INSR, MGAM, PYGL, NR5A2, AMY2A, PPARA, PPARD, PPARG, PTPN9, PDK2, RXRA, and RBP4.

A cutoff docking score of −9 kcal/mol was set to distinguish between potential active and inactive compounds. The predicted compound–target network was generated by Cytoscape version 3.4.0 [90], and the NetworkAnalyzer Application version 2.7 [91] was used to evaluate some of the basic network features.

### 3.2. Clustering and Maximum Common Substructure Analysis of Predicted Active Compounds

A hierarchical clustering analysis was performed for each compound–target group using Schrödinger Canvas Suite version 3.2.013 [92]. The molecular fingerprint was calculated from the two-dimensional structure of the compounds in the form of extended connectivity fingerprint 4 (ECFP4). From these fingerprints, a hierarchical clustering analysis was performed using the metric of the Tanimoto similarity and the average cluster linkage method, which clusters according to the average distance between all of the inter-cluster pairs. An MCS analysis was then performed on the largest cluster identified within each compound–target group using the criteria of atomic number, aromaticity, and bond order.

### 3.3. Similarity Studies with Known/Experimental Anti-Diabetic Drugs

The known/experimental anti-diabetic drugs were sourced from Defronzo et al., 2014 [2], and Gougari et al., 2017 [3], and their SMILES representations were obtained from PubChem. The molecular similarity network was generated with Cytoscape and the ChemViz2 Application version 1.1.0 [93]. The molecular similarity was performed using the metric of the Tanimoto similarity on the calculated ECFP4 molecular fingerprints of the compounds. A Tanimoto score of 0.7 or greater indicated molecular similarity. 

### 3.4. Studies on Oral Bioavailability and ADMET Properties of the Predicted Active Compounds

The physiochemical descriptors of molecular weight, AlogP, hydrogen bond acceptors, hydrogen bond donors, number of rotatable bonds, and polar surface area were calculated from the two-dimensional structures of the compounds using the Schrödinger Canvas Suite [92]. For the calculation of the QikProp descriptors, three-dimensional structures of the compounds were generated and optimized with LigPrep from Schrödinger Maestro version 11.2.013 [94]. The QikProp descriptors of aqueous solubility (QPlogS), Caco-2 cell permeability, binding to human serum albumin, percent human oral absorption, and blockage of the hERG K+ channels, were subsequently calculated from the three-dimensional structures with the Schrödinger Canvas Suite. The ProTox-II web server was used to predict the potential toxicity of the compounds from their SMILES notation representations [95]. The rat oral lethal dose 50 (LD50), hepatotoxicity, carcinogenicity, cytotoxicity, mutagenicity, and immunotoxicity were evaluated with the ProTox-II web server.

## 4. Conclusions

**Figure 6:**
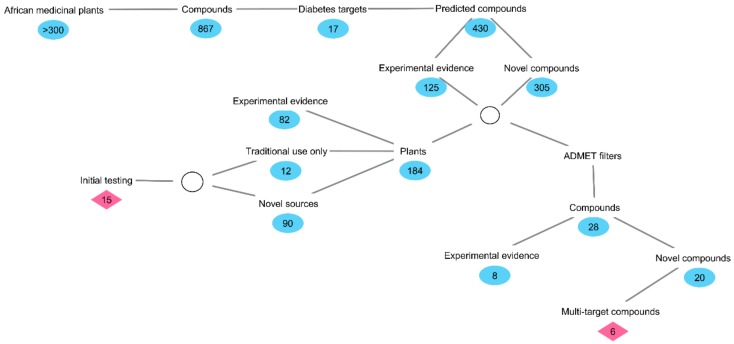
A brief summary of the methodology and results obtained for the *in silico* exploration of African medical plants for potential anti-diabetic compounds.

African medicinal plants were identified as rich sources of compounds with potential anti-diabetic activity through the use of inverse virtual screening with the DIA-DB web server (Figure 6). The observation that some of the compounds identified with the DIA-DB web server had some previous literature on their potential anti-diabetic activity provided validation for the use of the DIA-DB web server for the identification of compounds with potential anti-diabetic activity. Also, the identification of compounds with previous literature on their potential anti-diabetic activity provided some clues as to the bioactive constituents of medicinal plants with known anti-diabetic activity, as well as the rationale for the traditional use of some medicinal plants.

Several plants were identified as new sources rich in compounds with potential anti-diabetic activity, and included Argemone ochroleuca, Clivia miniata, Crinum bulbispermum, Danais fragans, Dioscorea dregeana, Dodonaea angustifolia, Eucomis autumnalis, Gnidia kraussiana, Melianthus comosus, Mondia whitei, Pelargonium sidoides, Typha capensis, Vinca minor, Voacanga Africana, and Xysmalobium undulatum. These plants represent a good initial point for exploratory *in vitro* anti-diabetic studies. As for the compounds, a total of 28 compounds were identified as having favorable ADMET properties, and importantly, several of these were identified as novel potential multi-targeted anti-diabetic compounds, such as crotofoline A, erythraline, henningsiine, nauclefidine, vinburnine, and voaphylline. These compounds present as novel scaffolds for further drug design and development. There is now the need for further *in vitro* and in vivo studies to confirm the potential bioactivity of these compounds identified by the DIA-DB web server.

## Figures and Tables

**Table 1:** The docking scores obtained for the ligands crystallised with protein targets versus the lowest energy obtained for a test compound.

Mode of Action	Protein Target	Function	PDB Code	Crystallized Ligand–Docking Score (kcal/mol)	Test Compounds–Lowest Energy (kcal/mol)	Test Compound Name
Regulation of insulin secretion and sensitivity	DPP4	Degrades and inactivates glucagon-like peptide-1 that stimulates insulin secretion from the pancreas [15]	4A5S	−10.5	−11.8	Cryptospirolepine
	FFAR1	Binding of free fatty acids to the receptor results in increased glucose-stimulated insulin secretion [16]	4PHU	−9.8	−11.6	Procyanidin C1
	HSD11B1	Coverts inactive glucocorticoid precursors to active glucocorticoids; glucocorticoids counteract the effects of insulin [17]	4K1L	−8.3	−12.8	Cryptomisrine
	INSR	Regulates glucose uptake, as well as glycogen, lipid, and protein synthesis [15]	3EKN	−8.7	−10.9	Typharin
	PTPN9	Dephosphorylates the insulin receptor, thereby reducing insulin sensitivity [18]	4GE6	−7.7	−10.2	Cryptospirolepine
	RBP4	Secreted as an adipokine that reduces insulin signaling and promotes gluconeogenesis [19]	2WR6	−7.9	−11	Benzo[c]phenanthridine
Regulation of glucose metabolism	AKR1B1	Catalyses the reduction of glucose to sorbitol in the polyol pathway, and plays a role in diabetic complications [20]	3G5E	−11.3	−11.9	Pterygospermin
	AMY2A	Hydrolyses alpha-1,4-glycosidic bonds to starch during digestion of starch to glucose [21]	4GQR	−7.9	−11.5	Clivimine
	GCK	Phosphorylates glucose to glucose-6-phosphate for glycolysis or glycogen synthesis [18]	3IMX	−10.6	−13	Cryptomisrine
	MGAM	Hydrolyzes 1,4-alpha bonds, the last step in the digestion of starch to glucose [21]	3L4Y	−5.7	−10	Cryptospirolepine
	PDK2	Responsible for inactivating the pyruvate dehydrogenase complex that is involved during glucose oxidation [22]	4MPC	−7.8	−11.5	Clivimine
	PYGL	Catalyses the first step of glycogenolysis by the phosphorolysis of glycogen to glucose-1-phosphate [23]	3DDS	−9.6	−10.8	Cryptomisrine
Regulation of lipid metabolism	NR5A2	Regulates the expression of the genes involved in bile acid synthesis, cholesterol synthesis, and steroidogenesis [24]	4DOR	−6.5	−12.2	Clivimine
	PPARA	Regulates the expression of the genes involved in lipid metabolism, in particular, the oxidation of fatty acids, as well as lipoprotein assembly and lipid transport [25]	3FEI	−8.3	−11.4	Biscryptolepine
	PPARD	Regulates the expression of the genes involved in fatty acid catabolism [25]	3PEQ	−11.3	−14.3	Cryptomisrine
	PPARG	Regulates the expression of the genes involved in adipogenesis and lipid metabolism, particularly fatty acid transport, lipid droplet formation, triacyglycerol metabolism, and lipolysis of triglycerides [25]	2FVJ	−10	−11.9	Cryptoquindoline
	RXRA	Heterodimerizes with PPARs, thereby initiating gene transcription [25]	1FM9	−10.6	−10.9	Crinasiatine

Aldose reductase (AKR1B1); dipeptidyl peptidase-4 (DPP4); free fatty acid receptor 1 (FFAR1); glucokinase (GCK); hydroxysteroid 11-beta dehydrogenase 1 (HSD11B1); insulin receptor (INSR); intestinal maltase-glucoamylase (MGAM); liver glycogen phosphorylase (PYGL); liver receptor homolog-1 (NR5A2); pancreatic alpha-amylase (AMY2A); peroxisome proliferator-activated receptor alpha (PPARA); peroxisome proliferator-activated receptor delta (PPARD); peroxisome proliferator-activated receptor gamma (PPARG); protein tyrosine phosphatase non-receptor type 9 (PTPN9); pyruvate dehydrogenase kinase isoform 2 (PDK2); retinoid X receptor alpha (RXRA); retinol binding protein 4 (RBP4).

**Table 2:** Plants with no previous anti-diabetic evidence, identified by virtual screening and their predicted bioactive compounds.

Plant Name	Family	Compounds
*Acokanthera oppositifolia*	Apocynaceae	Acolongifloroside K^31^, acovenoside A^32^, ouabain^304^
*Adenium multiflorum*	Apocynaceae	Obebioside^294^
*Agapanthus africanus*	Amaryllidaceae	Agapanthagenin^36^
*Amaryllis belladonna*	Amaryllidaceae	Acetylcaranine^30^, caranine*^97^, lycorine^277^
*Anagallis arvensis*	Primulaceae	Arvenin I^60^, arvenin II^61^
*Asclepias fruticosa*	Apocynaceae	Afroside^35^, 19-deoxyuscharin^20^, gomphoside^195^
*Aster bakeranus*	Asteraceae	ent-16-Kauren-18-oic-acid^162^, ent-16-Kauren-19-oic-acid^163^, friedelin*^174^
*Balanites maughamii*	Zygophyllaceae	Cryptogenin^127^, diosgenin*^153^
*Bersama lucens*	Melianthaceae	Melianthugenin^282^
*Boophane disticha*	Amaryllidaceae	3-Acetylnerbowdine^16^, buphanisin^93^
*Bowiea volubilis*	Asparagaceae	Bovogenin A^89^, bovoside A^90^
*Brabejum stellatifolium*	Proteaceae	Amygdalin*^51^
*Cestrum laevigatum*	Solanaceae	Parquin^310^
*Chrysanthemum cinerariifolium*	Asteraceae	Pyrethrin I^330^
*Clivia miniata*	Amaryllidaceae	Cliviamartine^112^, cliviasine^113^, clividine^114^, clivimine^115^, clivonine^116^, hippeastrine^217^, lycorine^277^
*Cotyledon orbiculata*	Crassulaceae	Orbicuside A^302^, tyledoside C^397^
*Crinum bulbispermum*	Amaryllidaceae	Acetylcaranine^30^, bulbispermine^92^, crinamine^122^, crinasiadine^123^, crinasiatine^124^, galanthamine^180^, hippeastrine^217^, lycorine^277^, pratorimine^319^
*Crinum macowanii*	Amaryllidaceae	Crinamine^122^, lycorine^277^, pratorimine^319^
*Crotalaria spartioides*	Fabaceae	Retrorsine^343^
*Croton gratissimus*	Euphorbiaceae	Crotofolin A^125^, crotonin^126^
*Cucumis africanus*	Cucurbitaceae	Cucurbitacin B^133^
*Cyclamen persicum*	Primulaceae	Cyclamin^137^
*Cynanchum africanum*	Apocynaceae	Cynafoside B^139^
*Danais fragans*	Rubiaceae	1-Hydroxydimethylanthraquinone^8^, kaempferol-3-O-rhamnodiglucoside^250^, quercitrin*^335^, rubiadin^348^, rubiadin xyloglucoside^349^
*Datura stramonium*	Solanaceae	Hyoscyamine^220^
*Delphinium grandiflorum*	Ranunculaceae	Nudicauline^293^
*Digitalis purpurea*	Plantaginaceae	Digitoxin^150^
*Dioscorea dregeana*	Dioscoreaceae	Deltonin^145^, deltoside^146^, dioscin*^152^, diosgenin*^153^, hircinol*^218^
*Dodonaea angustifolia*	Sapindaceae	Beta-sitosterol*^70^, hautriwaic acid^205^, stigmasterol*^375^
*Drimia robusta*	Hycinthaceae	12-Beta-hydroxyscillirosidin^4^, proscillardin A^324^
*Eriocephalus africanus*	Asteraceae	Ivangustine^246^
*Erythrina caffra*	Fabaceae	Erythraline^169^
*Erythrina lysistemon*	Fabaceae	Erythraline^169^
*Erythrophleum lasianthum*	Fabaceae	Erythrophleine^170^
*Eschscholzia californica*	Papaveraceae	Dihydrosanguinarine*^151^
*Eucomis autumnalis*	Asparagaceae	Autumnariniol^65^, autumnariol^66^, 3,9-dihydroeucomnalin^19^, eucosterol^171^
*Euphorbia ingens*	Euphorbiaceae	Ingenol^231^
*Ficus salicifolia*	Moraceae	Aviprin^69^
*Geigeria ornativa*	Asteraceae	Vermeerin^407^
*Geranium incanum*	Geraniaceae	Geraniin*^189^
*Gnidia kraussiana*	Thymelaeaceae	Gnidicin^192^, gnidilatin^193^, gniditrin^194^, 12-hydroxydaphnetoxin^5^
*Griffonia simplicifolia*	Fabaceae	Indole-3-acetyl aspartic acid^230^
*Homeria pallida*	Iridaceae	1,2-Epoxyscillirosidin^1^
*Hyaenanche globosa*	Picrodendraceae	Urushiol III^402^
*Hypericum aethiopicum*	Hypericaceae	Hypericin^222^
*Ipomoea purpurea*	Convolvulaceae	Ergine^167^
*Kalanchoe lanceolata*	Crassulaceae	Lanceotoxin A^258^, hellebrigenin^210^
*Lippia rehmannii*	Verbenaceae	Icterogenin^229^, lantadene A^259^
*Lotononis laxa*	Fabaceae	Integerrimine^234^, senecionine^359^
*Melianthus comosus*	Francoaceae	3-Epioleanolic acid*^17^, hellebrigenin-3-acetate^211^, melianthugenin^282^, oleanolic acid*^299^
*Melilotus alba*	Fabaceae	Dicoumarol^148^
*Moraea polystachya*	Iridaceae	16-Beta-formyloxybovogenin A^7^
*Mundulea sericea*	Fabaceae	Deguelin^142^, rotenone^347^, tephrosin^384^
*Ocotea bullata*	Lauraceae	Ocubullenone^295^
*Peddiea africana*	Thymelaeaceae	Peddiea factor A1^311^
*Pelargonium sidoides*	Geraniaceae	Catechin*^100^, gallocatechin*^181^, quercetin*^331^, sitosterol-3-glucoside*^364^
*Phytolacca dodecandra*	Phytolaccaceae	Lemmatoxin^262^, oleanoglycotoxin^298^
*Plumbago auriculata*	Plumbaginaceae	Plumbagin*^318^
*Polygala fruticosa*	Polygalaceae	Frutinone A^175^, presenegenin^321^
*Ptaeroxylon obliquum*	Rutaceae	Umtatin^22^
*Quercus robur*	Fagaceae	Catalagin*^99^, digallic acid^149^
*Rapanea melanophloeos*	Primulaceae	3-Oxo-20,24-dammaradien-26-ol^18^, sakurasosaponin^353^
*Rhododendron indicum*	Ericaceae	Grayanotoxin I^197^
*Rhus undulata*	Anacardiaceae	Apigenin dimethylether^56^
*Sanseviera hyacinthoides*	Asparagaceae	Ruscogenin-(25S)-form^350^
*Sarcostemma viminale*	Apocynaceae	Sarcovimiside B^356^
*Scabiosa columbaria*	Caprifoliaceae	Chlorogenic acid*^106^
*Scadoxus puniceus*	Amaryllidaceae	Haemanthamine^206^, haemanthidine^207^
*Schotia brachypetala*	Fabaceae	3,3,4,5,5-Pentahydroxystilbene*^14^
*Scilla natalensis*	Asparagaceae	Proscillardin A^324^
*Senecio retrorsus*	Asteraceae	Retrorsine^343^
*Senecio serratuloides*	Asteraceae	Platyphylline^317^, senecionine^359^
*Smodingium argutum*	Anacardiaceae	3,8,11-Heptadecadienylcatechol^15^
*Solanum pseudocapsicum*	Solanaceae	Solanocapsine^367^
*Spirostachys africana*	Euphorbiaceae	Stachenol^372^, stachenone^373^
*Strophanthus speciosus*	Apocynaceae	Christyoside^107^
*Synadenium grantii*	Euphorbiaceae	4-Deoxy-13-O-phenylacetyl-12-O-tigloylphorbol^21^
*Synaptolepis kirkii*	Thymelaeaceae	Synaptolepis factor K1^381^, synaptolepis factor K7^382^
*Tetradenia riparia*	Lamiaceae	Ibozol^228^, 8-(14)-15-isopimaradiene-7,18-diol^26^
*Thesium minkwitzianum*	Santalaceae	Thesinine^389^
*Thesium hystrix*	Santalaceae	Quercetin*^331^
*Thevetia peruviana*	Apocynaceae	Thevetin A^390^, thevetin B^391^
*Tylecodon wallichii*	Crassulaceae	Cotyledoside^121^
*Typha capensis*	Typhaceae	Catechin*^100^, typhaphtalide^398^, typharin^399^, thyphasterol^400^
*Urginea maritima*	Asparagaceae	Scillaren A^357^, scillarenin^358^
*Urginea sanguinea*	Asparagaceae	Scillaren A^357^
*Valeriana capensis*	Valerianaceae	Valerenic acid^405^
*Vinca minor*	Apocynaceae	Eburnamonine*^160^, vincamine*^417^
*Xerophyta retinervis*	Velloziaceae	Amentoflavone*^49^
*Zanthoxylum capense*	Rutaceae	Sanguinarine*^354^

The numbers 1–430 serves as the identification of each compound in Figure 1. * All of the compounds identified with some previous literature on their potential anti-diabetic activity.

**Table 3:** Summary of hierarchical clustering and maximum common substructure found in the largest cluster for each protein target group.

Target Enzyme	Total Number of Compounds	Largest Cluster	Cluster Centroid	Maximum Common Substructure
11HSDB1	208	40	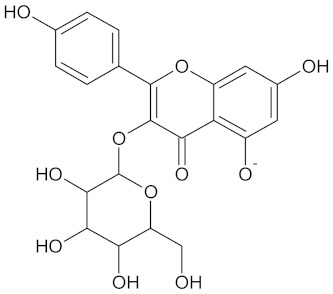 Kaempferol-3-glucoside	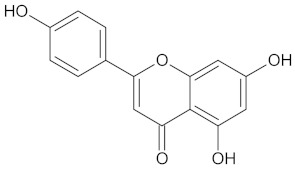
AKR1B1	135	71	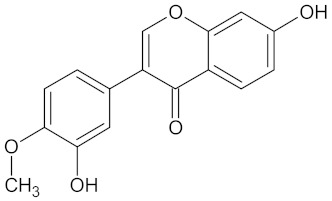 Calycosin	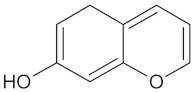
AMY2A	129	38	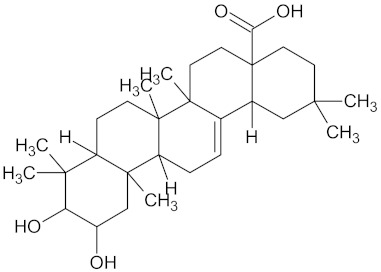 Maslinic acid	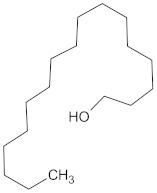
DPP4	149	23	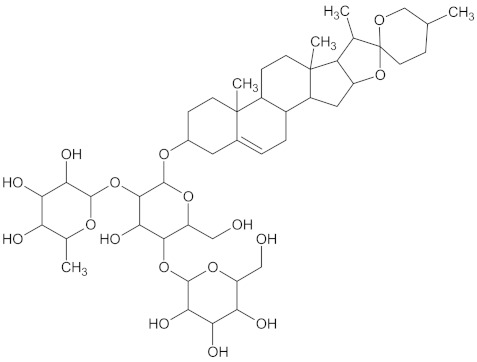 Balanitin-6	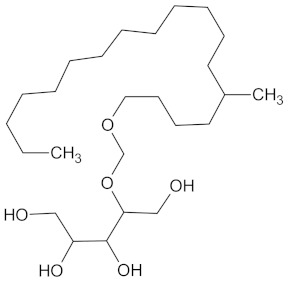
FFAR1	37	26	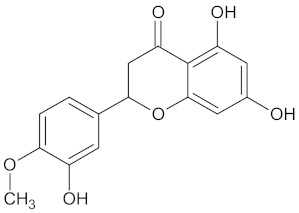 Hesperitin	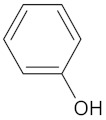
GCK	77	33	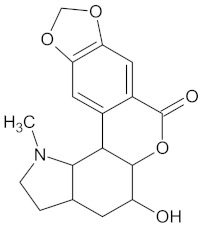 Clivonine	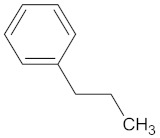
MGAM	18	12	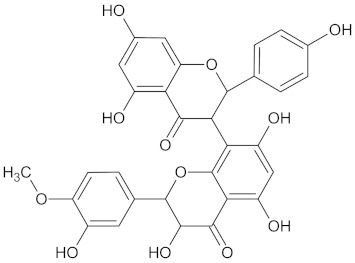 Kolaflavanone	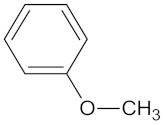
PPARD	190	57	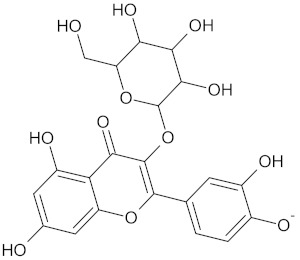 Hyperin	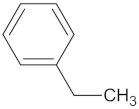
PPARG	124	89	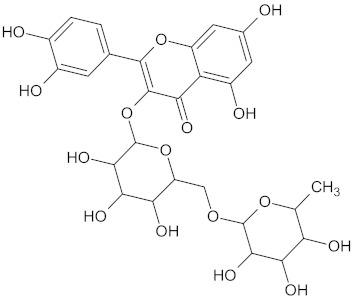 Rutin	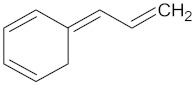
RBP4	85	48	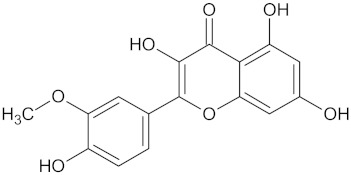 Isorhamnetin	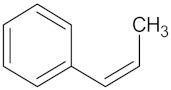

**Table 4:** Summary of the Absorption, Distribution, Metabolism, Excretion and Toxicity (ADMET) parameters predicted *in silico* for predicted active compounds versus diabetes drugs.

ADMET Property	Unknown Compounds	Known Compounds	Diabetes Drugs
Lipinski violations (1–4)	136/305 (45%)	75/125 (60%)	12/48 (25%)
Veber violations (1–2)	89/305 (29%)	42/125 (36%)	9/48 (19%)
Aqueous solubility QPlogS	34/305 (11%)	33/125 (26%)	6/48 (13%)
Caco-2 cell permeability (<25 nm/s)	66/305 (22%)	40/125 (32%)	3/48 (6%)
Binding to human serum albumin	37/305 (12%)	23/125 (18%)	6/48 (13%)
Human oral absorption (<25%)	55/305 (18%)	32/125 (26%)	3/48 (6%)
Rat oral LD_50_ (1–50 mg/kg)	53/305 (17%)	4/125 (3%)	1/48 (2%)
Hepatotoxicity	4/305 (1%)	4/125 (3%)	8/48 (17%)
Carcinogenicity	70/305 (23%)	31/125 (25%)	6/48 (13%)
Immunotoxicity	233/305 (76%)	89/125 (71%)	16/48 (33%)
Mutagenicity	49/305 (16%)	17/125 (14%)	1/48 (2%)
Cytotoxicity	58/305 (19%)	11/125 (9%)	1/48 (2%)
Blockage of hERG K^+^ channels	132/305 (43%)	58/125 (46%)	20/48 (42%)

* Recommended values: QPlogS: predicted aqueous solubility should be between −6.5 and 0.5 mol dm^−3^; Caco-2 cell permeability: <25 nm/s poor and >500 nm/s great; Binding to human serum albumin: QPlogKhsa should be between −1.5 and 1.5; Human oral absorption: <25% poor and >80% great; Rat oral LD_50:_ <50mg/kg is fatal if swallowed; Blockage of hERG K^+^ channels: concern if predicted QPlogHERG is <−5.

**Table 5:** Predicted active compounds with favorable ADMET properties.

Compound	Structure	Predicted Targets (Docking Score in kcal/mol)	Potential Anti-Diabetic Effect	Plant
2-Hydroxygenistein	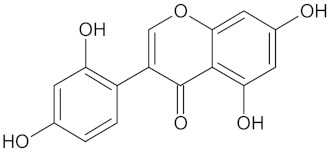	AKR1B1 (−9.1)	Regulation of glucose metabolism	*Cajanus cajan*
Apigenin	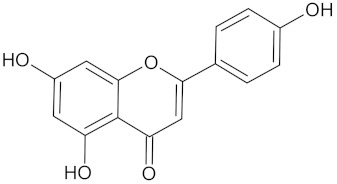	AKR1B1 (−9.1), HSD11B1 (−9.0), RBP4 (−9.9), and RXRA (−9.1)	Regulation of insulin secretion, glucose metabolism, and lipid metabolism	*Cajanus cajan*
Autumnarinol	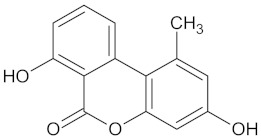	RBP4 (−9.0)	Regulation of insulin secretion	*Eucomis autumnalis*
Catechin	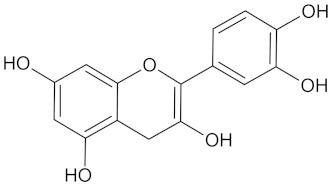	AKR1B1 (−9.0), HSD11B1 (−9.5), and RBP4 (−9.3)	Regulation of insulin secretion and glucose metabolism	*Adansonia digitate*, *Combretum micranthum*, *Prunus africana*, *Sclerocarya birrea*, *Pelargonium sidoides*, and *Typha capensis*
Crotofoline A	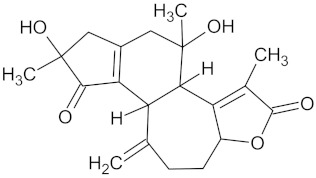	AMY2A (−9.2), HSD11B1 (−9.9), and PPARD (−9.3)	Regulation of insulin secretion, glucose metabolism, and lipid metabolism	*Croton gratissimus*
Cyanidin	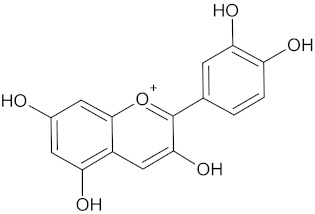	AKR1B1 (−9.1), HSD11B1 (−9.5), and RBP4 (−9.2)	Regulation of insulin secretion and glucose metabolism	*Rhoicissus tridentate*
Desacetylformonoakuammiline	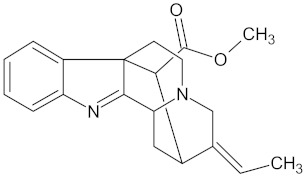	HSD11B1 (−9.1), PPARD (−9.0)	Regulation of insulin secretion and lipid metabolism	*Rauvolfia vomitoria*
Eburnamonine	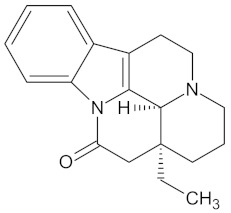	AKR1B1 (-9.4), HSD11B1 (−9.2), PPARD (−9.3), and RBP4 (−9.4)	Regulation of insulin secretion, glucose metabolism, and lipid metabolism	*Vinca minor*
Ent-16-kauran-19-oic acid	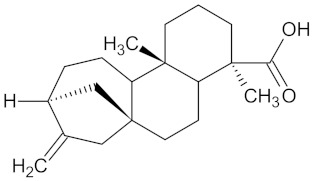	HSD11B1 (−9.4) and PPARD (−9.4)	Regulation of insulin secretion and lipid metabolism	*Aster bakeranus*
Epicatechin	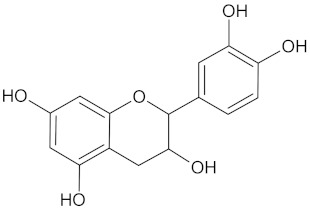	AKR1B1 (−9.2) and RBP4 (−9.3)	Regulation of insulin secretion and glucose metabolism	*Acacia karroo*, *Harungana madagascariensis*, and *Prunus Africana*
Ergine	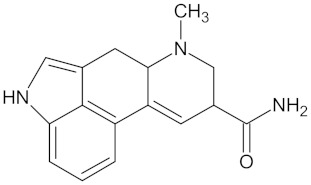	HSD11B1 (−9.5) and RBP4 (−9.4)	Regulation of insulin secretion	*Ipomoea purpurea*
Eriodictyol	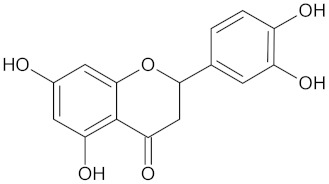	HSD11B1 (−9.2) and RBP4 (−9.5)	Regulation of insulin secretion	*Cyclopia intermedia*
Erythraline	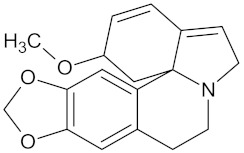	AKR1B1 (−9.0), GCK (−9.8), and RBP4 (−9.0)	Regulation of insulin secretion and glucose metabolism	*Erythrina caffra* and *Erythrina lysistemon*
Furanoeudesma-1,3-diene	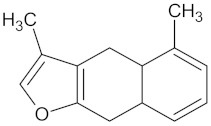	RBP4 (−9.0)	Regulation of insulin secretion	*Commiphora myrrha*
Hautriwaic acid	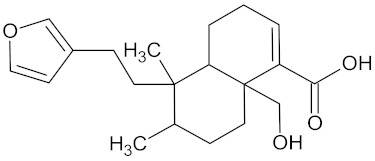	AKR1B1 (−9.3)	Regulation of glucose metabolism	*Dodonaea angustifolia*
Henningsiine	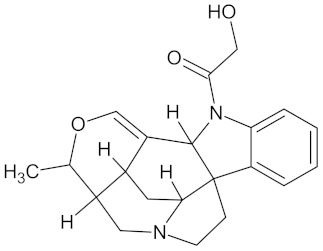	AMY2A (−9.1), HSD11B1 (−9.6), PPARD (−10.0), and PPARG (−9.0)	Regulation of insulin secretion, glucose metabolism, and lipid metabolism	*Strychnos henningsii*
Ibozol	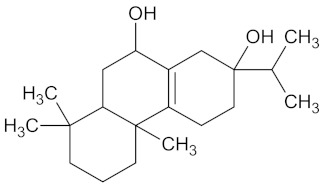	GCK (−9.7)	Regulation of glucose metabolism	*Tetradenia riparia*
Integerrimine	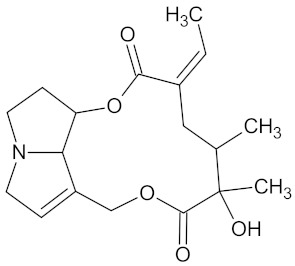	HSD11B1 (−9.1) and PPARD (−9.3)	Regulation of insulin secretion and lipid metabolism	*Lotononis laxa*
Lapachol	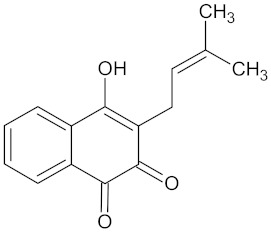	AKR1B1 (−9.2)	Regulation of glucose metabolism	*Kigelia africana*
Nauclefidine	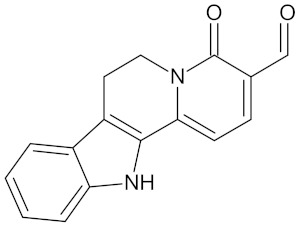	AKR1B1 (−10.1), HSD11B1 (−9.0), and RBP4 (−10.0)	Regulation of insulin secretion and glucose metabolism	*Nauclea latifolia*
N-methylflindersine	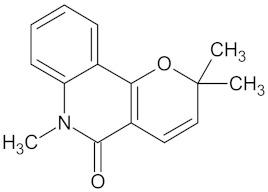	AKR1B1 (−9.2) and RBP4 (−9.5)	Regulation of insulin secretion and glucose metabolism	*Toddalia asiatica*
Platyphylline	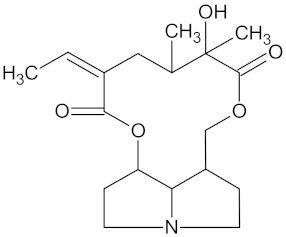	HSD11B1 (−9.4) and PPARD (−9.3)	Regulation of insulin secretion and lipid metabolism	*Senecio serratuloides*
Rhinocerotinoic acid	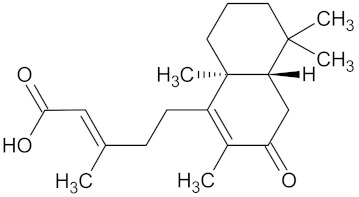	HSD11B1 (−9.2) and RBP4 (−9.9)	Regulation of insulin secretion	*Elytropappus rhinocerotis*
Senecionine	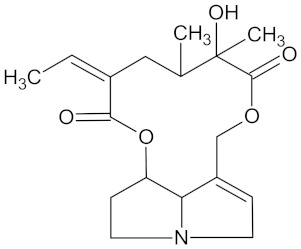	HSD11B1 (−10.3) and PPARD (−9.4)	Regulation of insulin secretion and lipid metabolism	*Senecio serratuloides*
Valerenic acid	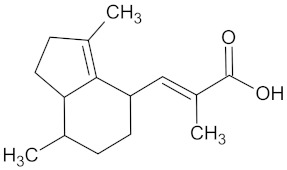	AKR1B1 (−9.0)	Regulation of glucose metabolism	*Valeriana capensis*
Vinburnine	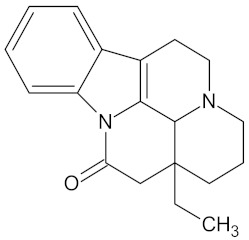	AKR1B1 (−9.6), HSD11B1 (−9.1), PPARD (−9.3), PPARG (−9.4), RBP4 (−10.7), and RXRA (−9.3)	Regulation of insulin secretion, glucose metabolism and lipid metabolism	*Voacanga africana*
Voaphylline	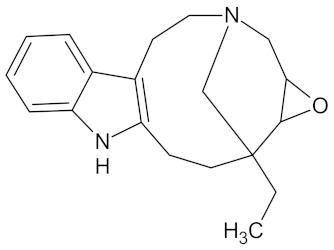	AMY2A (−9.0), DPP4 (−9.6), GCK (−9.1), HSD11B1 (−9.3), PPARD (−9.1), PPARG (−9.8), and RBP4 (−9.2)	Regulation of insulin secretion, glucose metabolism, and lipid metabolism	*Voacanga africana*
Withasomnine	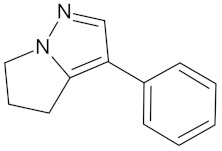	FFAR1 (−9.1)	Regulation of insulin secretion	*Voacanga africana*

## Supplementary Materials

The following are available online at https://www.mdpi.com/1420-3049/24/10/2002/s1: Table S1—SMILES notations of all compounds evaluated in the study. Table S2—Assigned numerical identity of predicted active compounds, their plant sources and predicted targets. Figure S1—Individual predicted active compound–protein target networks. Table S3—Plants having scientific anti-diabetic evidence and evidence of traditional use only identified by virtual screening and their predicted bioactive compounds. Figure S2—Dendrograms of hierarchical clustering analysis.
