# Supplementary material for: Exploring African Medicinal Plants for Potential Anti-Diabetic Compounds with the DIA-DB Inverse Virtual Screening Web Server
Source: Molecules. 2019 May 24;24(10):2002. doi: 10.3390/molecules24102002 (PMC6571761; doi:10.3390/molecules24102002)
Supplement: Supplementary file 1 [file molecules-24-02002-s001.pdf]

Supplementary Materials

Exploring African medicinal plants for potential anti-diabetic compounds with the DIA-DB inverse virtual screening web server.

Andreia S.P. Pereira[1], Helena den Haan[2], Jorge Peña-García[2], Marién M. Moreno[2], Horacio Pérez-Sánchez[2,*], Zeno Apostolides[1,*]

[1] Department of Biochemistry, Genetics and Microbiology, University of Pretoria, Pretoria, South Africa; aspdpereira@gmail.com (A.S.P.P.); zeno.apostolides@up.ac.za (Z.A.)

[2] Structural Bioinformatics and High Performance Computing Research Group (BIO-HPC), Universidad Católica de Murcia, Spain; hden@alu.ucam.edu (H.d.H.); Jorge.dlpg@gmail.com (J.P.-G.); memoreno@ucam.edu (M.M.M.); hperez@ucam.edu (H.P.-S.)

* Correspondence: hperez@ucam.edu; Tel: +34-968278819 (H.P.-S.); zeno.apostolides@up.ac.za; Tel: +27-12-420-2486 (Z.A.)

Table of contents	Pages
**Table S1**. SMILES notations of all compounds evaluated in the study	2-32
**Table S2.** Assigned numerical identity of the predicted active compounds, their plant sources and predicted targets.	33-52
**Figure S1.** Individual predicted active compound-protein target networks	53-54
**Table S3.** Plants having scientific anti-diabetic evidence and evidence of traditional use only identified by virtual screening and their predicted bioactive compounds.	55-58
**Figure S2.** Dendrograms of hierarchical clustering analysis	59-62

Table S1. SMILES notations of all compounds evaluated in the study

Compound name	SMILES
(+)-Toddanol	C=C(C)[C@@H](Cc1c(cc2c(ccc(=O)o2)c1OC)OC)O
[7]-Paradol	CCCCCCCCC(=O)CCc1ccc(c(c1)OC)O
1,10,5-Germacrodien-4-ol	CC(C)[C@@H]1CC/C(=C\CC[C@@](C)(/C=C\1)O)/C
1,2-Epoxyscilliroscidin	CC(=O)OC1C[C@@]2(C(CC[C@]3(C)C[C@H](C[C@]23O)c2ccc(=O)oc2)[C@]2(C)C1=C[C@H]([C@H]1[C@@H]2O1)O)O
1,3,6,8-Tetrahydroxy-2,5-dimethoxyxanthone	COC1=C(C2C(C=C1O)Oc1c(c(cc(c1OC)O)O)C2=O)O
1,5-Dicaffeoylquinic acid	C1C(C(C(CC1(C(=O)O)OC(=O)C=CC2=CC(=C(C=C2)O)O)OC(=O)C=CC3=CC(=C(C=C3)O)O)O)O
1,5-Dihydroxy-3-methylanthrquinone	Cc1cc2c(c(c1)O)C(=O)c1ccc(cc1C2=O)O
1,8-Cineole	CC1(C)C2CC[C@@](C)(CC2)O1
10-Deoxyeucommiol	OCC[C@@H]1C(CO)=C(C)C[C@H]1O
10-Hydroxyakagerine	C/C=C(/C=O)\[C@@H]1C[C@H]2c3c(CCN2C)c2cc(ccc2n3[C@H](C1)O)O
12B-Hydroxyscillirosidin	CC(=O)O[C@@H]1C[C@@]2([C@H](CC(=O)[C@]3(C)[C@H](CC[C@]23O)C2=COC(=O)OC2)[C@@]2(C)CC[C@@H](C=C12)O)O
12-Hydroxydaphnetoxin	C=C(C)[C@]12[C@@H](C(C)[C@]34C5C=C(C)C(=O)[C@]5([C@@H]([C@]5(CO)[C@H]([C@H]3[C@H]1OC(c1ccccc1)(O2)O4)O5)O)O)O
12-O-Palmitoyl-13-O-acetyl-16-hydroxyphorbol	CCCCCCCCCCCCCCCC(=O)O[C@@H]1[C@@H](C)[C@]2([C@@H](CC=C(C[C@]3([C@H]2C=C(C)C3=O)O)CO)[C@@H]2[C@@](C)(CO)[C@]12OC(=O)C)O
15-Acetoxy-14-hydroxycostundide	C=C1[C@@H]2CC/C(=C\CC/C(=C\[C@H]2OC1=O)/COC(=O)C)/CO
15B-Hydroxygomphoside	C[C@@H]1C[C@H]([C@@]2(C[C@@H]3C[C@@]4(C)C(CC[C@@H]5C4CC[C@]4(C)[C@H](C[C@H]([C@]54O)O)C4=CC(=O)OC4)C[C@H]3C[C@@H]2O1)O)O
15-Oxo-kaurenoic acid	C=C1[C@@H]2CC[C@H]3[C@]4(C)CCC[C@](C)([C@H]4CC[C@]3(C2)C1=O)C(=O)O
16B-Formyloxybovogenin A	C[C@@]12CCC3C(CC[C@H]4C[C@H](CC[C@]34CO)O)[C@]2(C[C@@H]([C@@H]1c1ccc(=O)oc1)OC=O)O
19-Deoxyuscharin	C[C@@H]1CC2([C@]3([C@@H](O1)O[C@@H]1C[C@@H]4CC[C@@H]5C(CC[C@]6(C)C(CC[C@]56O)C5=CC(=O)OC5)[C@@]4(C)C[C@H]1O3)O)N=CCS2
19-Hydroxyglaucolide A	C=C(CO)C(=O)OC1CC(C)(OC(C)=O)C(=O)CCC3(C)OC3C2OC(=O)C(COC(C)=O)=C12
1B,3B-Dihydroxy-5,16-pregnadien-20-one	CC(=O)C1=CCC2C3CC=C4C[C@H](C[C@H]([C@]4(C)C3CC[C@]12C)O)O
1-Hydroxydimethyl anthraquinone	Cc1cc2c(c(c1C)O)C(=O)c1ccccc1C2=O
2-(1-Hydroxyethyl)naphtho[2,3-b]furan-4,9-dione	C=C(C)c2cc3C(=O)c1ccccc1C(=O)c3o2
24-Ethylcholest-5-en-3B-ol	CC(=CCC[C@@H](C)[C@H]1CC[C@@]2(C)[C@@H]3CC[C@H]4C(C)(C)[C@H](CC[C@@]54C[C@]35CC[C@]12C)O)C
2-Hydroxy-4-methoxybenzaldehyde	COC1=CC(=C(C=C1)C=O)O
2-Hydroxygenistein	C1=CC(=C(C=C1O)O)C2=COC3=CC(=CC(=C3C2=O)O)O
2-Methoxyfuranodiene	CC1=CCC2=C(CC(=CC(C1)OC)C)OC=C2C

2-Methyl-6,3-methyl-2-butenylbenzo-1,4-quinone	CC(=CCC1=CC(=O)C=C(C)C1=O)C
2-Undecanone	CCCCCCCCCC(=O)C
2α,3α,23-Trihydroxyurs-12-en-28-oic acid	C[C@@H]1CC[C@@]2(CC[C@]3(C)C(=CC[C@@H]4[C@@]5(C)C[C@@H](O)[C@@H](O)[C@@](C)(CO)[C@@H]5CC[C@@]34C)[C@@H]2[C@H]1C)C(O)=O
2α,3α-Dihydroxyurs-12-en-28-oic acid	C[C@H]1[C@@H]([C@@H]2[C@@](CC[C@@]3(C2=CC[C@H]4[C@]3(CC[C@@H]5[C@@]4(C[C@H]([C@H]([C@]5(C)C)O)O)C)C)C)(CC1)C(=O)O)C
2α,3β-Dihydroxyurs-12-en-18-oic acid	C[C@H]1[C@@H]([C@@H]2[C@@](CC[C@@]3(C2=CC[C@H]4[C@]3(CC[C@@H]5[C@@]4(C[C@H]([C@H]([C@]5(C)C)O)O)C)C)C)(CC1)C(=O)O)C
2α,3β-Dihydroxyurs-12-en-28-oic acid	CC1CCC2(CCC3(C(=CCC4C3(CCC5C4(CC(C(C5(C)C)O)O)C)C)C2C1C)C)C(=O)OC6C(C(C(C(O6)CO)O)O)O
2α-Hydroxyursolic acid	C[C@@H]1CC[C@@]2(CC[C@]3(C)C(=CC[C@@H]4[C@@]5(C)C[C@H]([C@@H](C(C)(C)[C@@H]5CC[C@@]34C)O)O)[C@@H]2[C@H]1C)C(=O)O
3-(2'-hydroxyethyl)-5-(2''-hydroxypropyl)-dihydrofuran-2(3H)-one	OC(C)CC1CC(CCO)C(=O)O1
3,3,4,5,5-Pentahydroxystilbene	C(=C\c1cc(c(c(c1)O)O)O)/c1cc(cc(c1)O)O
3,3,4-Dihydroxyphenyl-1,2-propane diol	c1cc(c(cc1CC(CO)O)O)O
3,8,11-Heptadecadienylcatechol	CCCCC/C=C\C/C=C\CCCCCCCc1cccc(c1O)O
3,9-Dihydroeucomnalin	Oc1ccc(cc1)CC2COc3cc(O)c(OC)c(O)c3C2=O
3-Acetoxybrachylaenolide	C=C1[C@@H]2CC[C@@]3(C)C=C[C@H](C(=C)C3[C@H]2OC1=O)OC(=O)C
3-Acetylnerbowdine	CC(=O)O[C@@H]1C[C@@H]2[C@@]3(CCN2Cc2c3cc3c(c2OC)OCO3)[C@@H](C1)O
3B,24-Dihydroxyurs-12-en-28-oic acid	C[C@@H]1CC[C@@]2(CC[C@@]3(C(=CC[C@H]4[C@]3(CC[C@@H]5[C@@]4(CC[C@@H]([C@]5(C)CO)O)C)C)[C@@H]2[C@H]1C)C)C(=O)O
3B-Hydroxynorerythrosuamine	O=C(OCCNC)\C=C3/CCC2C([C@@H](O)C(=O)C1[C@]2(C)CC[C@H](O)[C@]1(C)C(=O)OC)[C@H]3C
3B-Hydroxynorerythrosuamine-β-D-glucopyranosyl	O=C(OCCNC)\C=C1/CCC3C([C@H]1C)[C@@H](O)C(=O)C4[C@](C)([C@@H](OC2OC(CO)C(O)C(O)C2O)CC[C@]34C)C(=O)OC
3-Coumaric acid	c1cc(ccc1/C=C/C(=O)O)O
3-Epioleanolic acid	CC1(C)CCC2(CC[C@]3(C)C(=CCC4[C@@]5(C)CC[C@H](C(C)(C)C5CC[C@@]34C)O)C2C1)C(=O)O
3-Hydroxy-4-methoxy-3-methyloxidole	CC1(c2c(cccc2OC)NC1=O)O
3-Hydroxyheterodendrin	CC(C)([C@@H](C#N)OC1C(C(C(C(CO)O1)O)O)O)O
3-Oxo-20,24-dammaradien-26-ol	C/C(=C\CCC(=C)[C@H]1CC[C@]2(C)[C@@H]1CCC1[C@@]3(C)CCC(=O)C(C)(C)C3CC[C@@]21C)/CO
3-O-α-D-galactopyranosyl-L-arabinose	C(C1[C@H](C([C@H]([C@H](O1)O[C@H]1[C@H](COC([C@H]1O)O)O)O)O)O)O
3-O-β-D-Glucopyranosoyl rosmarinic acid	c1cc(c(cc1/C=C/C(=O)O[C@@H](Cc1ccc(c(c1)OCC(C(C(C(C=O)O)O)O)O)O)C(=O)O)O)O
3-O-β-L-arabinopyranosyl-L-arabinose	O[C@H]2CO[C@H](O)[C@@H](O)C2O[C@H]1OC[C@H](O)C(O)[C@@H]1O
4,5-Hydroxy-5,16-nonadecenyl-2-cyclohexen-1-one	CC/C=C/CCCCCCCCCCCCCCC[C@@H]1CC(=O)C=C[C@@H]1O

4,5α-Epoxy-6α-hydroxy-1(10)- E-11(13)-germacradien-12,8α-olide	C/C/1=C/CC[C@@]2(C)[C@@H]([C@H]([C@@H]3C(=C)C(=O)O[C@H]3C1)O)O2
411-Eudesmonediol	CC(C)([C@@H]1CC[C@@]2(C)CCC[C@](C)(C2C1)O)O
4-Deoxy-13-O-phenylacetyl-12-O-tiloylphorbol	C/C=C(/C)\C(=O)OC1[C@@H](C)[C@@]2([C@@H]3C=C(C)C(=O)C3CC(=C[C@H]2[C@@H]2C(C)(C)[C@]12OC(=O)CC1CCCCC1)CO)O
4-Hydroxyacetophenone	CC(=O)c1ccc(cc1)O
4-Hydroxymethylanthrixianone	C=C1C[C@]23C=CC4=C(CO)C(=O)CC[C@@]4(C)[C@@H]3CC[C@H]1C2
4-Hydroxyphenyl-N-methylaziridine	CN1CC1c1ccc(cc1)O
4-Methoxy-1-Methyl-2,1H-quinolinone	Cn1c2cccc(c2ccc1=O)OC
4-Methoxy-1-methyl-2-quinolone	Cn1c2ccccc2c(cc1=O)OC
4-O-Ethylgallic acid	C(CO)c1c(cc(cc1O)C(=O)O)O
4-O-Methylpyridoxine	Cc1c(c(COC)c(cn1)CO)O
4α,18-Epoxyafricanin	C[C@@H]1CC(=O)[C@]2(COC(=O)C)[C@H](CCC(=O)[C@@]32CO3)[C@]21C[C@@H](c1ccoc1)OC2=O
5,6,7-Trimethoxycoumarin	COc1cc2c(ccc(=O)o2)c(c1OC)OC
5,7,4-Trihydroxy-3,6-dimethylmethoxyflavone	COc1c(cc2c(c1O)c(=O)c(c(c1cccc(c1)O)o2)OC)O
5,7,8-Trimethoxycoumarin	COc1cc(c(c2c1ccc(=O)o2)OC)OC
5,7-Dihydroxy-6-methoxy-3(4 hydroxybenzyl)chroman-4-one	c1cc(c(cc1CC1COc2cc(c(c(c2C1=O)O)O)O)O)O
5-Caffeoylquinic acid	C1C(C(C(CC1(C(=O)O)O)OC(=O)C=CC2=CC(=C(C=C2)O)O)O)O
5-Chloropropacin	CC1C(OC2=C(O1)C3=C(C=CC(=O)O3)C(=C2OC)Cl)C4=CC(=C(C=C4)O)OC
5-Hydroxyaloin A	C1=CC(=C2C(=C1O)C(C3=CC(=CC(=C3C2=O)O)CO)C4C(C(C(C(O4)CO)O)O)O)O
5-Hydroxyhexamethoxyflavanol	COc1cc(cc(c1OC)OC)c1c(c(=O)c2c(c(c(c(c2o1)OC)OC)OC)O)O
5-Hydroxylindole-3-acetic acid	c1cc2c(cc1O)c(CC(=O)O)c[nH]2
5-Hydroxytryptophan	c1cc2c(cc1O)c(CC(C(=O)O)N)c[nH]2
5-Methoxysuberenon	CC(=O)/C=C/c1c(cc2OC(=O)C=Cc2c1OC)OC
5-O-Acetyl-4-O-methylpyridoxine	Cc1c(c(COC)c(cn1)COC(=O)C)O
5α-Hydroxy-10B-hydroprintzionic acid	C[C@@H]1CC[C@]2(CC(=CCC[C@@H]2[C@@]1(C)CCc1ccoc1)C(=O)O)O
6-(2-Hydroxy-3-methoxy-3-methylbutyl)-5,7-dimethoxycoumarin	CC(C)(OC)C(O)Cc1c(cc2OC(=O)C=Cc2c1OC)OC
6-(3-Chloro-2-hydroxy-3-methylbutyl)-5,7-dimethoxycoumarin	CC(C)(Cl)C(O)Cc1c(cc2OC(=O)C=Cc2c1OC)OC
6,7-Dihydro-6,7-ocimenediol	CC(C)(O)C(O)C\C=C(/C)C=C
6-Formyllimettin	COc1cc2OC(=O)C=Cc2c(OC)c1C=O
6-Gingerol	CCCCC[C@@H](CC(=O)CCc1ccc(c(c1)OC)O)O

6-Hydroxy-5,7-dimethoxycoumarin-8-sulfate	COc1c2ccc(=O)oc2c(c(c1O)OC)S
6-Methyldiosgenin	C[C@H]6C2C(CC3C4CC(C)=C1C[C@@H](O)CC[C@]1(C)C4CC[C@]23C)O[C@@]56CCC(C)CO5
6-Paradol	CCCCCCCC(=O)CCC1=CC(=C(C=C1)O)OC
6-Shogaol	CCCCCC=CC(=O)CCC1=CC(=C(C=C1)O)OC
7-Hydroxy-10-deoxyeucommiol	CC1=C(CO)[C@@H](CCO)[C@H](O)[C@@H]1O
7-Hydroxy-4-6-dimethoxypropacin	COc1cc(cc(OC)c1O)[C@H]2Oc3c(O[C@@H]2C)c4OC(=O)C=Cc4cc3OC
7-Hydroxyeucommic acid	OCC1=C(CO)[C@@H](CC(=O)O)[C@H](O)[C@@H]1O
7-hydroxyviteoid II	O=C1OCC[C@@H]2C1=C(CO)[C@@H](O)[C@H]2O
7-Methyljuglone	Cc1cc2c(c(c1)O)C(=O)C=C(C2=O)O
8-(3,3-Dimethylallyl)-6,7-dimethoxycoumarin	CC(=CCc1c2c(ccc(=O)o2)cc(c1OC)OC)C
8,(14),15-Isopyrimadine-7,18-diol	C=C[C@@]1(C)CCC2C(=C1)[C@@H](CC1[C@@](C)(CCC[C@]21C)CO)O
8-Formyllimmetin	COc2cc(OC)c(C=O)c1OC(=O)C=Cc12
8-Hydroxypinoresinol	COC1=C(C=CC(=C1)C2C3COC(C3(CO2)O)C4=CC(=C(C=C4)O)OC)O
9-Geranyl-a-terpineol	CC(=CCC/C(=C/CCC(C)(C1CC=C(C)CC1)O)/C)C
9Z-Octadec- 9-enamide	CCCCCCCC/C=C\CCCCCCCC(=O)N
Abrine	CN[C@@H](Cc1c[nH]c2ccccc12)C(=O)O
Acacic acid	CC1(C)C[C@H]2C3=CCC4[C@@]5(C)CC[C@@H](C(C)(C)C5CC[C@@]4(C)[C@]3(C)C[C@H]([C@]2(C[C@@H]1O)C(=O)O)O)O
Acalyphin	CN1C(=O)C=C(C(C#N)(C1O)OC1C(C(C(C(CO)O1)O)O)O)OC
Acetylcaranine	CC(=O)OC1CC=C2CCN3Cc4cc5c(cc4[C@H]1[C@@H]23)OCO5
Acolongifloroside K	CC1C(C(C(C(O1)OC2CC(C3(C4C(CCC3(C2)O)C5(CCC(C5(CC4O)C)C6=CC(=O)OC6)O)CO)O)O)O)O
Acorenone	CC(C)[C@@H]1CC[C@H](C)C21CC=C(C)C(=O)C2
Acovenoside A	CC1C(C(C(C(O1)OC2CC3CCC4C(C3(C(C2)O)C)CCC5(C4(CCC5C6=CC(=O)OC6)O)C)O)OC)O
Adiantone	CC1(C)CCC[C@@H]2C3CC[C@@H]4[C@@]5(C)CC[C@H](COC)[C@@H]5CC[C@@]4(C)[C@@H]3CCC12
Africanol	CC1CCC2C1(CC(CC3C2(C3)C)(C)C)O
Afromosin	COC1=CC=C(C=C1)C2=COC3=CC(=C(C=C3C2=O)OC)O
Afroside	C[C@@H]1C[C@H]([C@]2([C@@H](O1)O[C@@H]1C[C@@H]3CCC4C(CCC5[C@H](C[C@H]([C@]45O)O)C4=CC(=O)OC4)C3C[C@H]1O2)O)O.CC.CC
Agapanthagenin	CC1CCC2(C(C)C3C(CC4C5CC[C@]6(C[C@H]([C@@H](C[C@]6(C)C5CC[C@]34C)O)O)O)O2)OC1
Ajmalicine	CC1[C@H]2CN3CCc4c5ccccc5[nH]c4[C@@H]3C[C@@H]2C(=CO1)C(=O)OC
Ajmaline	CCC1C2CC3C4C5(CC(C2C5O)N3C1O)C6=CC=CC=C6N4C
Ajugol	CC1(CC(C2C1C(OC=C2)OC3C(C(C(C(O3)CO)O)O)O)O)O
Akuammine	CC=C1CN2CCC34C5=C(C=CC(=C5)O)N(C36C2CC1C4(CO6)C(=O)OC)C

Allantoin	C(N)ON[C@@H]1C(=O)NC(=O)N1
Alliin	C=CCS(=O)CC(C(=O)O)N
Allopurinol	c1c2c(ncnc2O)[nH]n1
Allouzarin	CC12CCC(CC1CCC3C2CCC4(C)C(CCC34O)C5=CC(=O)OC5)OC6OC(CO)C(O)C(O)C6OC7OC(CO)C(O)C(O)C7O
Alloxysmalorin	CC12CCC(CC1=CCC3C2CCC4(C)C(CCC34O)C5=CC(=O)OC5)OC6OC(CO)C(O)C(O)C6OC7OC(CO)C(O)C(O)C7O
Aloe-emodin anthrone	c1cc2c(c(c1)O)C(=O)c1c(cc(cc1O)CO)C2=O
Aloenin	Cc1cc(cc(c1c1cc(cc(=O)o1)OC)OOCC(C(C(C(C=O)O)O)O)O)O
Aloeresin A	CC1=CC(=C(C2=C1C(=O)C=C(O2)CC(=O)C)C3C(C(C(C(O3)CO)O)O)OC(=O)C=CC4=CC=C(C=C4)O)O
Aloeresin B	CC1=CC(=C(C2=C1C(=O)C=C(O2)CC(=O)C)C3C(C(C(C(O3)CO)O)O)O)O
Aloesin	Cc1cc(c(c2c1C(=O)CC(CC(=O)C)O2)OCC(C(C(C(C=O)O)O)O)O)O
Aloin A	c1cc2c(c(c1)O)C(=O)c1c(cc(cc1O)CO)[C@H]2OCC(C(C(C(CO)O)O)O)O
Aloinoside A	C[C@H]1[C@@H]([C@H]([C@H]([C@H](OCc2cc3[C@H](c4cccc(c4C(=O)c3c(c2)O)O)[C@H]2[C@@H]([C@H]([C@@H]([C@@H](CO)O2)O)O)O)O1)O)O)O
Aloinoside B	CC1C(C(C(C(OCc2cc3C(c4cccc(c4C(=O)c3c(c2)O)O)C2C(C(C(C(CO)O2)O)O)O)O1)O)O)O
Ambelline	CO[C@H]1C=CC23c4cc5c(c(c4CN(C[C@@H]3O)[C@@H]2C1)OC)OCO5
Amentoflavone	c1cc(ccc1c1cc(=O)c2c(cc(c(c3cc(ccc3O)c3cc(=O)c4c(cc(cc4o3)O)O)c2o1)O)O)O
Amritoside	C1=C2C3=C(C(=C1O)O)OC(=O)C4=CC(=C(C(=C43)OC2=O)O)OC5C(C(C(C(O5)COC6C(C(C(C(O6)CO)O)O)O)O)O)O
Amygladin	c1ccc(cc1)C(C#N)OC1C(C(C(C(COC2C(C(C(C(CO)O2)O)O)O)O1)O)O)O
Anabasine	c1cc(cnc1)[C@@H]1CCCN1
Anemonin	C1=CC2(CC3(C=CC(=O)O3)C2)OC1=O
Anethole	C/C=C/c1ccc(cc1)OC
Angustine	C=CC1=C2C=C3C4=C(CCN3C(=O)C2=CN=C1)C5=CC=CC=C5N4
Angustoline	CC(C1=C2C=C3C4=C(CCN3C(=O)C2=CN=C1)C5=CC=CC=C5N4)O
Anolignan B	C=C(Cc1ccc(cc1)O)C(=C)Cc1ccc(cc1)O
Antidesmone	Cc1nc(O)c2c(c1OC)[C@H](CCC2=O)CCCCCCCC
Aphloiol	c1c2c(cc(c1O)O)oc1cc(c(c(c1c2=O)O)C1C(C(C(C(CO)O1)O)O)O)O
Apigenin	c1cc(ccc1c1cc(=O)c2c(cc(cc2o1)O)O)O
Apigenin dimethylether	COc1ccc(cc1)C1CC(=O)c2c(cc(cc2O1)OC)O
Arachidic acid	CCCCCCCCCCCCCCCCCCCC(=O)O
Arbutin	c1cc(ccc1O)OCC(C(C(C(C=O)O)O)O)O
Arcinine	CC1C2CN3CCC4=C(C3CC2C(=CO1)C(=O)OC)NC5=C4C=C(C=C5)OC
Arjunglucoside	CC1(CCC2(CCC3(C(=CCC4C3(CCC5C4(CC(C(C5(C)CO)O)O)C)C)C2C1O)C)C(=O)OC6C(C(C(C(O6)CO)O)O)O)C

Arjunolic acid	CC1(C)CC[C@@]2(CC[C@]3(C)C(=CCC4[C@@]5(C)C[C@H]([C@@H]([C@@](C)(CO)C5CC[C@@]34C)O)O)[C@]2(C)C1)C(=O)O
Arnottianamide	CN(C=O)c1c(ccc2cc3c(cc12)OCO3)c1ccc(c(c1O)OC)OC
Arteglasin A	CC1=C2C[C@@H]3[C@](C)(C2[C@@H]2[C@H](C(=C)C(=O)O2)[C@H](C1)OC(=O)C)O3
Artemisyl acetate	CC(=CC(C(C)(C)C=C)OC(=O)C)C
Arvenin I	CC(=O)OC(C)(C)/C=C/C(=O)[C@@](C)([C@H]1[C@@H](C[C@@]2(C)[C@@H]3CC=C4[C@@H](C[C@@H](C(=O)C4(C)C)O[C@H]4[C@@H]([C@H]([C@@H]([C@@H](CO)O4)O)O)O)[C@]3(C)C(=O)C[C@]12C)O)O
Arvenin II	CC(=O)OC(C)(C)CCC(=O)[C@@](C)([C@H]1[C@@H](C[C@@]2(C)[C@@H]3CC=C4[C@@H](C[C@@H](C(=O)C4(C)C)O[C@H]4[C@@H]([C@H]([C@@H]([C@@H](CO)O4)O)O)O)[C@]3(C)C(=O)C[C@]12C)O)O
Ascaricin	C=CCc1cc2c(cc1OC)OCO2
Ascaridole	CC(C)C12C=CC(C)(CC1)OO2
Asiatic acid	C[C@@H]1CCC2(CC[C@]3(C)C(=CCC4[C@@]5(C)C[C@H]([C@@H]([C@@](C)(CO)C5CC[C@@]34C)O)O)[C@@H]2[C@H]1C)C(=O)O
Asiaticoside	CC1CCC2(CCC3(C(=CCC4C3(CCC5C4(CC(C(C5(C)CO)O)O)C)C)C2C1C)C)C(=O)OC6C(C(C(C(O6)COC7C(C(C(C(O7)CO)OC8C(C(C(C(O8)C)O)O)O)O)O)O)O)O
Aspalathin	c1cc(c(cc1CCC1c2c(cc(c(c2O[H-]O1)OCC(C(C(C(CO)O)O)O)O)O)O)O)O
Aspirin	CC(=O)Oc1ccccc1C(=O)O
Atractyloside	CC(C)CC(=O)OC1C(C(C(OC1OC2CC(C3CCC45CC(CCC4C3(C2)C)C(=C)C5O)C(=O)O)CO)OS(=O)(=O)O)OS(=O)(=O)O
Atraric acid	Cc1cc(c(C)c(c1C(=O)OC)O)O
Atropine	CN1[C@H]2CCC1CC(C2)OC(=O)[C@H](CO)c1ccccc1
Autumnariniol	Cc2cc(O)c(OC)c3OC(=O)c1c(cccc1O)c23
Autumnariol	Cc1cc(cc2c1c1cccc(c1c(=O)o2)O)O
Avenasterol	C/C=C(/CC[C@@H](C)[C@H]1CC[C@H]2C3=CC[C@H]4C[C@H](CC[C@]4(C)C3CC[C@]12C)O) \ C(C)C
Avicine	C[n+]1cc2cc3c(cc2c2ccc4cc5c(cc4c12)OCO5)OCO3
Aviprin	CC(C)(C[C@@H](O)Oc1c2ccc(=O)oc2cc2c1cco2)O
Balagyptin	CC1C(C(C(C(O1)OCC2C(C(C(C(O2)OC3CCC4(C5CCC6(C(C5CC=C4C3)CC(C6C(C)O)O)C)C)OC7C(C(C(C(O7)C)O)O)O)O)O)O)O)O
Balanitin 4	CC1CCC2(C(C3C(O2)CC4C3(CCC5C4CC=C6C5(CCC(C6)OC7C(C(C(C(O7)CO)OC8C(C(C(C(O8)CO)O)OC9C(C(C(C(O9)CO)O)O)O)O)O)O)OC2C(C(C(C(O2)C)O)O)O)C)C)C)OC1
Balanitin 6	CC1CCC2(C(C3C(O2)CC4C3(CCC5C4CC=C6C5(CCC(C6)OC7C(C(C(C(O7)CO)OC8C(C(C(C(O8)CO)O)O)O)O)OC9C(C(C(C(O9)C)O)O)O)C)C)C)OC1
Balanitin 7	CC1CCC2(C(C3C(O2)CC4C3(CCC5C4CC=C6C5(CCC(C6)OC7C(C(C(C(O7)CO)OC8C(C(C(C(O8)CO)O)OC9C(C(C(CO9)O)O)O)O)O)OC2C(C(C(C(O2)C)O)O)O)C)C)C)OC1

Balanitine 1	OC%10C(O)C(O)C(C)OC%10OC9C(O)C(O)C(CO)OC9OC2C(O)C(OC1OC(C)C(O)C(O)C1O)C(OC2CO)OC7CC8=CCC3C(CCC4(C)C6C(CC34)OC5(CCC(C)CO5)C6C)C8(C)CC7
balanitine 2	OC9C(O)C(O)C(C)OC9OC8C(O)C(O)C(CO)OC8OC1C(O)C(O)C(OC1CO)OC6CC7=CCC2C(CCC3(C)C5C(CC23)OC4(CCC(C)CO4)C5C)C7(C)CC6
balanitine 3	CC%10C2C(CC3C4CC=C1CC(CCC1(C)C4CCC23C)OC8OC(CO)C(O)C(OC6OC(COC5OCC(O)C(O)C5O)C(O)C(O)C6O)C8OC7OC(C)C(O)C(O)C7O)OC9%10CCC(C)CO9
Balanitoside	CC1C2C(CC3C2(CCC4C3CC=C5C4(CCC(C5)OC6C(C(C(C(O6)CO)OC7C(C(C(C(O7)CO)O)O)O)O)OC8C(C(C(C(O8)C)O)O)O)C)C)OC1(CCC(C)COC9C(C(C(C(O9)CO)O)O)O)O
Ballotenol	C[C@@H]1C(=O)[C@H](C2C(C)(C)CCC[C@]2(C)[C@]1(CCc1ccoc1)O)O
Barringenol A	CC1(C)C[C@H]2C3=CCC4[C@@]5(C)CC[C@@H](C(C)(C)C5CC[C@@]4(C)[C@]3(C)[C@H]([C@H]([C@@]2(CO)[C@H](C1)O)O)O)O
Bauerenol	CC1CCC2(CCC3(C4=CCC5C(C(CCC5(C4CCC3(C2C1C)C)C)O)(C)C)C)C
Behenic acid	CCCCCCCCCCCCCCCCCCCCCC(=O)O
Benzo[c]phenanthridine	c1ccc2c(c1)ccc1c3ccccc3cnc21
Benzoic acid	C1=CC=C(C=C1)C(=O)O
Berberine	COc1ccc2cc3c4cc5c(cc4CC[n+]3cc2c1OC)OCO5
Bergapten	COc1c2ccc(=O)oc2cc2c1cco2
Berulide	CCCCC/C=C/C/C=C/CCCCCCCC(=O)OCC(COC(=O)CCCCCCC/C=C/C/C=C/CCCCC)OC(=O)CCCCCCCCCCCCCCC
Betain	C[N+](C)(C)CC(=O)[O-]
Betulinic acid	C=C(C)C1CC[C@@]2(CC[C@]3(C)[C@H](CCC4[C@@]5(C)CC[C@@H](C(C)(C)[C@@H]5CC[C@@]34C)O)[C@@H]12)C(=O)O
Biochanin A	COC1=CC=C(C=C1)C2=COC3=CC(=CC(=C3C2=O)O)O
Bisabolene	CC(=CCC/C(=C/1\CC=C(C)CC1)/C)C
Biscryptolepine	CN2c1ccccc1C(=C3N=C4C=CC=CC4=C23)C7=C6N=C5CC=CCC5=C6N(C)c8ccccc78
Borneol	CC1(C)C2CC[C@]1(C)[C@@H](C2)O
Bovogenin A	C[C@@]12CCC3[C@@H](CC[C@H]4C[C@H](CC[C@]34CO)O)[C@]2(CC[C@@H]1c1ccc(=O)oc1)O
Bovoside A	CC1C(C(C(C(O1)O[C@H]1CC[C@@]2(CO)[C@@H](CCC3C2CC[C@]2(C)[C@H](CC[C@]32O)c2ccc(=O)oc2)C1)O)OC)O
Brassinolide	CC(C)C(C)C(C(C(C)C1CCC2C1(CCC3C2COC(=O)C4C3(CC(C(C4)O)O)C)C)O)O
Brunfelsamidine	c1c[nH]cc1C(=N)N
Bulbispermine	C1=CC23c4cc5c(cc4CN(CC3O)[C@H]2C[C@H]1O)OCO5
Buphanamine	COC1=C2CN3CCC4(C3CC=CC4O)C2=CC5=C1OCO5
Buphanidrine	CO[C@H]1C=C[C@@]23CCN(Cc4c2cc2c(c4OC)OCO2)[C@@H]3C1
Buphanisin	COC1CC2C3(CCN2CC4=CC5=C(C=C43)OCO5)C=C1

Butyric acid	CCCC(=O)O
Caespitin	CC(=CCc1c(cc(c(C(=O)CCC(C)C)c1O)O)O)C
Caffeic acid	c1cc(c(cc1/C=C/C(=O)O)O)O
Caffeine	Cn1cnc2c1c(=O)n(C)c(=O)n2C
Cajanol	COC1=CC(=C2C(=C1)OCC(C2=O)C3=C(C=C(C=C3)O)OC)O
Cajaquinone	CC1=C(C=C2C(=C1)C(=O)C3=CC(=CC(=C3C2=O)OC)O)O
Calcium oxalate	C(=O)(C(=O)[O-])[O-].[Ca+2]
Calycosin	COC1=C(C=C(C=C1)C2=COC3=C(C2=O)C=CC(=C3)O)O
Campesterol	CC(C)[C@H](C)CC[C@@H](C)[C@H]1CC[C@H]2[C@@H]3CC=C4C[C@H](CC[C@]4(C)[C@H]3CC[C@]12C)O
Camphene	C=C1C2CCC(C2)C1(C)C
Camphor	CC1(C)C2CCC1(C)C(=O)C2
Canavanine	CC(CCONC(=N)N)N
Cannabidiol	CCCCCC1=CC(=C(C(=C1)O)C2C=C(CCC2C(=C)C)C)O
Capsaicin	CC(C)/C=C/CCCCC(=O)NCc1ccc(c(c1)OC)O
Caranine	C1CN2CC3=CC4=C(C=C3C5C2C1=CCC5O)OCO4
Carapanaubine	CC1C2CN3CCC4(C3CC2C(=CO1)C(=O)OC)C5=CC(=C(C=C5NC4=O)OC)OC
Carboxyparquin	CC(C)CC(=O)OC1C(OC2C[C@@]3(C)C4CC[C@@H]5C[C@@]4(CC[C@@H]3C(C2)(C(=O)O)C(=O)O)C(C5=C)O)OC(CO)C(C1(CO)O)OC1C(C(=O)C(=O)O1)OCO
Cardanol	CCCCCC/C=C\CCCCCCCc1cccc(c1)O
Carene	CC1=CCC2C(C1)C2(C)C
Carnasol	CC(C)c1cc2[C@@H]3CC4C(C)(C)CCC[C@@]4(c2c(c1O)O)C(=O)O3
Carpusin	COC1=C2C(=CC(=C1)O)OC(C2=O)(CC3=CC=C(C=C3)O)O
Carvone	C=C(C)C1CC=C(C)C(=O)C1
Caryophyllene	C/C/1=C/CCC(=C)[C@@H]2CC(C)(C)[C@H]2CC1
Cassaine	C[C@H]1/C(=C/C(=O)OCCNC)/CC[C@H]2[C@H]1C(=O)C[C@H]1C(C)(C)[C@H](CC[C@]21C)O
Castalagin	C1C2C(C3C4C(C5=C(C(=C(C(=C5C(=O)O4)C6=C(C(=C(C(=C6C(=O)O3)C7=C(C(=C(C=C7C(=O)O2)O)O)O)O)O)O)O)O)O)O)OC(=O)C8=CC(=C(C(=C8C9=C(C(=C(C=C9C(=O)O1)O)O)O)O)O)O
Catechin	c1cc(c(cc1C1=C(Cc2c(cc(cc2O1)O)O)O)O)O
Catharanthine	CCC1=CC2CN3CCc4c5ccccc5[nH]c4[C@@]2(COC)C13
Cathinone	C[C@@H](C(=O)c1ccccc1)N
Celastrin	CC1CCC(C2(C13C(C(C(C2OC(=O)C4=CC=CC=C4)OC(=O)C)C(O3)(C)C)OC(=O)C)COC(=O)C)OC(=O)C
Centelloside	CC1(CCC2(CCC3(C(=CCC4C3(CC(C5C4(CC(C(C5(C)CO)O)O)C)O)C)C2C1)C)C(=O)OC6C(C(C(C(O6)COC7C(C(C(C(O7)CO)O)O)O)O)O)O)C

Chelerythrine	C[n+]1cc2c(ccc(c2OC)OC)c2ccc3cc4c(cc3c12)OCO4
Chironioside	O=C1OCCC2(O)C(C=C)C(OC=C12)OC4OC(COC3OCC(O)C(O)C3O)C(O)C(O)C4O
Chlereythrine-Ψ-cyanide	COc2ccc1c3ccc4cc5OCOc5cc4c3N(C)C(C#N)c1c2OC
Chlorogenic acid	c1cc(c(cc1/C=C/C(=O)OC1CC(CC(C1O)O)(C(=O)O)O)O)O
Christyoside	CC1C(C(C(C(O1)O[C@H]1CC[C@@]2(CO)[C@@H](CCC3C2CC[C@]2(C)[C@H](CC[C@]32O)C2=CC(=O)OC2)C1)O)O C)O
Chrysanthenyl acetate	CC1=CCC2C(C1C2(C)C)OC(=O)C
Chrysophonein	Cc1cc2c(c(c1)OCC(C(C(C(C=O)O)O)O)O)C(=O)c1c(cccc1O)C2=O
Chrysophonol	Cc1cc2c(c(c1)O)C(=O)c1c(cccc1O)C2=O
Cineole	CC1(C)C2CCC(C)(CC2)O1
Cinnamaldehyde	C1=CC=C(C=C1)C=CC=O
cis-Allocimene	CC=C(C)C=CC=C(C)C
Cissacapine	CN1CCc2cc(c3c4c2[C@H]1Cc1ccc2c(c1)COc1c(cc5CCN(C)[C@H](Cc6ccc(c(c6)CO3)O4)c5c1O2)OC)OC
Cissampreine	CN1CCc2cc(c(c3c2[C@H]1Cc1ccc(cc1)COc1c(cc2CCN=C(Cc4ccc(cc4)O3)c2c1O)OC)OC)OC
Citral	CC(=CCCC(=CC=O)C)C
Citric acid	C(C(=O)O)C(CC(=O)O)(C(=O)O)O
Citronellol	CC(=CCC[C@@H](C)CCO)C
Cliviamartine	CCOC(=O)C1=C(N=C(C(=C1)C(=O)OC2CC3CCN(C3C4C2OC(=O)C5=CC6=C(C=C45)OCO6)C)C)C
Cliviasine	CN4CCC5CC(O)C3OC(=O)c2cc1OCOc1cc2C3C45
Clividine	CN1CCC2C1C3c4cc5c(cc4C(=O)OC3C(C2)O)OCO5
Clivimine	CC1=C(C=C(C(=N1)C)C(=O)OC2CC3CCN(C3C4C2OC(=O)C5=CC6=C(C=C45)OCO6)C)C(=O)OC7CC8CCN(C8C9C7 OC(=O)C1=CC2=C(C=C91)OCO2)C
Clivonine	CN1CCC2C1C3C(C(C2)O)OC(=O)C4=CC5=C(C=C34)OCO5
Cnicin	C/C/1=C/CC/C(=C\[C@@H]2C(C(=C)C(=O)O2)[C@H](C1)OOCC(=C)C(CO)O)/CO
Coccinine	CO[C@@H]1C=C2[C@H]3CN(Cc4cc5c(cc34)OCO5)[C@]2(C[C@@H]1O)N
Colchicine	CC(=O)N[C@H]1CCc2cc(c(c(c2c2ccc(c(=O)cc12)OC)OC)OC)OC
Colosolic acid	CC1CCC2(CCC3(C)C(=CCC4C5(C)CC(C(C(C)(C)C5CCC34C)O)O)C2C1C)C(=O)O
Combretastatin A	COc1ccc(/C=C\c2cc(c(c(c2)OC)OC)OC)cc1O
Combretin A	O=C(O)[C@@]1(C)C2CCC4[C@@]3(C[C@]23CC[C@@H]1O)CC[C@]5(C)C(C[C@H](O)[C@@]45C)C(C)CCC(=C)C(C)C
Combretin B	CC(C)C(=C)CCC(C)C2C[C@H](O)[C@@]3(C)C4CCC6[C@@](C)([C@@H](OC1OCC(O)C(O)C1O)CC[C@@]65CC45CC[C@]23C)C(=O)O
Coniceine	CCCC1=NCCCC1
Coniine	CCC[C@H]1CCCCN1

Conyscabraic-acid	C[C@@H]1CC[C@H]2C[C@@]3(C=C[C@@H]3[C@@H]2[C@@]1(C)CCc1ccoc1)C(=O)O
Cornustannin A	c1c(cc(c(c1O)O)O)C(=O)OC1[C@@H]2C(COC(=O)c3cc(c(c(c3c3c(cc(c(c3O)O)O)C(=O)O2)O)O)O)O[C@H](C1OC(=O)c1cc(c(c(c1)O)O)O)OC(=O)c1cc(c(c(c1)O)O)O
Corsolic acid	C[C@@H]1CC[C@@]2(CC[C@]3(C)C(=CC[C@@H]4[C@@]5(C)C[C@H]([C@@H](C(C)(C)[C@@H]5CC[C@@]34C)O)O)[C@@H]2[C@H]1C)C(=O)O
Cotyledoside	CC1C([C@]2([C@H]([C@@H](O1)O[C@@H]1C[C@@H]3C[C@H]4[C@@]5(C(CC[C@]6(C)[C@H](CC[C@]56O)c5ccc(=O)oc5)[C@@]3(C)C[C@H]1O2)O4)O)OC)O
Coumurrayin	CC(=CCc1c(cc(c2ccc(=O)oc12)OC)OC)C
Crinamine	CO[C@H]1C=C[C@]23c4cc5c(cc4CN(CC3O)C2C1)OCO5
Crinasiadine	C1OC2=C(O1)C=C3C(=C2)C4=CC=CC=C4NC3=O
Crinasiatine	C1C(OC2=C(O1)C=C3C4=CC=CC=C4NC(=O)C3=C2)CC5=CC=C(C=C5)O
Crinine	C1CN2CC3=CC4=C(C=C3C15C2CC(C=C5)O)OCO4
Crotofoline A	C=C1CCC2C(=C(C)C(=O)O2)[C@@H]2[C@@H]1C1=C(C[C@@]2(C)O)C[C@](C)(C1=O)O
Crotonin	C[C@@H]1CC(=O)C[C@H]2[C@@H]1CC[C@@H](C)[C@@]12CC(c2ccoc2)OC1=O
Cryptogenin	CC(CCC(=O)C(C)C1C(=O)CC2C1(CCC3C2CC=C4C3(CCC(C4)O)C)C)CO
cryptoheptine	OC=1C=C4C(N=C2C=CC=CC=12)c3ccccc3N4C
Cryptolepine	CN1C2=CC=CC=C2C=C3C1=C4C=CC=CC4=N3
Cryptomisrine	C1=CC=C2C(=C1)C3=NC4=CC=CC=C4C(=C3N2)C(=O)C5=C6C(=NC7=CC=CC=C75)C8=CC=CC=C8N6
Cryptoquindoline	CN7C1=C8C=CC=CC8=NC1=C(n4c2ccccc2c3nc5ccccc5cc34)c6ccccc67
Cryptospirolepine	CN2c1ccccc1C57C(On3c5c2c4ccccc34)n9c6c(N(C)c8ccccc8C=C67)c%10ccccc9%10
Cucurbitacin B	CC(=O)OC(C)(C)/C=C/C(=O)[C@@](C)(C1[C@@H](C[C@@]2(C)C3CC=C4C(C[C@@H](C(=O)C4(C)C)O)[C@]3(C)C(=O)C[C@]12C)O)O
Cuminic aldehyde	CC(C)c1ccc(cc1)C=O
Cuminol	CC(C)c1ccc(cc1)CO
Curcanoleic acid	C(=C(\CC(=O)O)/C(=O)O)\C(=O)O
Curcumin	COc1cc(/C=C/C(=O)CC(=O)/C=C/c2ccc(c(c2)OC)O)ccc1O
Curcuson A	CC1CC2=C(C1=O)C3C(C=C(C2=O)C)C(CCC3=C)C(=C)C
Curcuson C	C=C(C)[C@@H]1CCC(=C)C2C1C=CCC1=C2C(=O)C(C)(C1)O
Cyanidin	c1cc(c(cc1c1c(cc2c(cc(cc2[o+]1)O)O)O)O)O
Cycasin	C/[N+](=N\COC1C(C(C(C(CO)O1)O)O)O)/[O-]
Cyclamin	CC1(C2CCC3(C(C2(CCC1OC4C(C(C(CO4)OC5C(C(C(C(O5)CO)O)OC6C(C(C(C(O6)CO)O)O)O)OC7C(C(C(CO7)O)O)O)O)OC8C(C(C(C(O8)CO)O)O)O)C)CCC91C3(CC(C2(C9CC(CC2)(C)C=O)CO1)O)C)C)C
Cycloartenol	CC(CCC=C(C)C)C1CCC2(C1(CCC34C2CCC5C3(C4)CCC(C5(C)C)O)C)C
Cymene	CC(C)c1ccc(C)cc1

Cynafoside B	CC(=O)[C@H]1CC[C@@]2(C3CC[C@H]4C[C@H](CC[C@]4(C)C3[C@@H]([C@H]([C@]12C)OC(=O)C1CCCCC1)OC(=O)C)OC1CC(C(C(C)O1)OC1CC(C(C(C)O1)OC1CC(C(C(C)O1)OC1C(C(C(C(CO)O1)O)O)O)OC)OC)O)O
Cynafoside H	CC(=O)C4CCC5(O)C2CCC1CC(CCC1(C)C2C(OC(C)=O)C(OC(=O)c3ccccc3)C45C)OC%10OC(C)C(OC9OC(C)C(OC8OC(C)C(OC7OC(CO)C(OC6OC(CO)C(O)C(O)C6O)C(O)C7O)C(OC)C8)C(OC)C9)C(OC)C%10
Cytisine	c1cc2[C@H]3C[C@@H](CNC3)Cn2c(=O)c1
D-Amphetamine	C[C@@H](Cc1ccccc1)N
Daucosterol	CC[C@H](CC[C@@H](C)[C@H]1CC[C@H]2[C@@H]3CC=C4C[C@H](CC[C@]4(C)[C@H]3CC[C@]12C)O[C@H]1[C@@H]([C@H]([C@@H]([C@@H](CO)O1)O)O)O)C(C)C
Deacetyldesformoakuammiline	O=C(OC)C5C2CC3C4=Nc1ccccc1C45CCN3C/C2=C\C
Deguelin	CC1(C)C=Cc2c(ccc3C(=O)[C@H]4c5cc(c(cc5OC[C@H]4Oc23)OC)OC)O1
Dehydrofalcarinol	C=CCCCCC/C=C/C/C=C/C=C/C(C=C)O
Delphinidin	c1c(cc(c(c1O)O)O)c1c(cc2c(cc(cc2[o+]1)O)O)O
Deltonin	C[C@@H]1CC[C@@]2([C@@H](C)[C@H]3[C@H](C[C@H]4[C@@H]5CC=C6C[C@H](CC[C@]6(C)[C@H]5CC[C@]34C)O[C@H]3[C@@H]([C@H]([C@@H]([C@@H](CO)O3)O[C@H]3[C@@H]([C@H]([C@@H]([C@@H](CO)O3)O)O)O)O)O[C@H]3[C@@H]([C@@H]([C@H]([C@H](C)O3)O)O)O)O2)OC1
Deltoside	C[C@H](CC[C@@]1(O)O[C@H]2C[C@H]3[C@@H]4CC=C5C[C@H](CC[C@]5(C)[C@H]4CC[C@]3(C)[C@H]2[C@@H]1C)O[C@@H]1O[C@H](CO)[C@@H](O[C@@H]2O[C@H](CO)[C@@H](O)[C@H](O)[C@H]2O)[C@H](O)[C@H]1O[C@@H]1O[C@@H](C)[C@H](O)[C@@H](O)[C@H]1O)CO[C@@H]1O[C@H](CO)[C@@H](O)[C@H](O)[C@H]1O
Deoxyloganin	CC1CCC2C1C(OC=C2C(=O)OC)OC3C(C(C(C(O3)CO)O)O)O
Des-N-methylchelerythrine	COC1=C(C2=CN=C3C(=C2C=C1)C=CC4=CC5=C(C=C43)OCO5)OC
Dhurrin	c1cc(ccc1[C@@H](C#N)OC1C(C(C(C(CO)O1)O)O)O)O
Dicoumarol	c1ccc2c(c1)c(c(Cc1c(c3ccccc3oc1=O)O)c(=O)o2)O
Dicrotaline	C[C@@]1(CC(=O)OCC2=CCN3CC[C@H]([C@@H]23)OC(=O)C1)O
Digallic acid	c1c(cc(c(c1O)O)OC(=O)c1cc(c(c(c1)O)O)O)C(=O)O
Digitoxin	CC1C(C(CC(O1)OC1C(C)OC(CC1O)OC1C(C)OC(CC1O)O[C@H]1CC[C@@]2(C)[C@H](CCC3C2CC[C@]2(C)[C@H](CC[C@]32O)C2=CC(=O)OC2)C1)O)O
Dihydrosanguinarine	CN1Cc2c(ccc3c2OCO3)c2ccc3cc4c(cc3c12)OCO4
Dioscin	C[C@@H]1CC[C@@]2([C@@H](C)[C@H]3[C@H](C[C@H]4[C@@H]5CC=C6C[C@H](CC[C@]6(C)[C@H]5CC[C@]34C)O[C@H]3[C@@H]([C@H]([C@@H]([C@@H](CO)O3)O[C@H]3[C@@H]([C@@H]([C@H]([C@H](C)O3)O)O)O)O)O[C@H]3[C@@H]([C@@H]([C@H]([C@H](C)O3)O)O)O)O2)OC1
Dioscorine	CC1=CC(=O)O[C@@]2(C1)C[C@H]1CC[C@@H]2CN1C
Diosgenin	C[C@@H]1CC[C@@]2([C@@H](C)C3C(CC4C5CC=C6CC(CC[C@]6(C)C5CC[C@]34C)O)O2)OC1
Diosmetin	COC1=C(C=C(C=C1)C2=CC(=O)C3=C(C=C(C=C3O2)O)O)O

Diosmin	C[C@@H]1[C@H]([C@@H]([C@@H]([C@@H](OCC(C(C(C(COc2ccc3c(=O)cc(c4ccc(c(c4)O)OC)oc3c2)O)O)O)O)O1)O)O)O
Diosphenol	CC(C)C1=C(C(=O)C(C)CC1)O
Diospyrin	Cc1cc2c(c(c1)O)C(=O)C=C(c1c(C)cc3C(=O)C=CC(=O)c3c1O)C2=O
Docosanol	CCCCCCCCCCCCCCCCCCCCCCO
Docosyl ferulate	CCCCCCCCCCCCCCCCCCCCCCOC(=O)/C=C/c1ccc(c(c1)OC)O
Dodonic acid	C[C@@H]1C[C@H]([C@]2(C)C(=CCC[C@@H]2[C@@]1(C)CCc1ccoc1)C(=O)O)O
Dregeana 4	CC(CC)C(OC(C)=O)C(=O)O[C@@H]3CC4[C@@]5(C)C(CC(=O)O[C@](C)(CO)C5C[C@@H](OC(=O)C(O)C(C)C)[C@@]4(C)C2=CC[C@@H](c1ccoc1)[C@@]23C)OC(C)=O
Dregeanin	CCC(C)C(C(=O)O[C@H]1[C@@H](C(C(=C)[C@@]23[C@@H](C[C@@H](c4ccoc4)[C@]12C)O3)[C@]1(C)C2CC(=O)O[C@]2(C)COC(=O)C[C@@H]1OC(=O)C)OC(=O)C)OC(=O)C
Durantoside I	C[C@@]1([C@H](C[C@@]2(C(=CO[C@H](C12)OC1C(C(C(C(CO)O1)O)O)O)C(=O)OC)O)OC(=O)/C=C/c1ccccc1)O
Durantoside II	CC1(C(CC2(C1C(OC=C2C(=O)OC)OC3C(C(C(C(O3)CO)O)O)O)O)OC(=O)C=CC4=CC=C(C=C4)OC)O
Durantoside III	COc1ccc(cc1OC)/C=C/C(=O)OC4CC3(O)C(=COC(OC2OC(CO)C(O)C(O)C2O)C3C4(C)O)C(=O)OC
Eburnamonine	CC[C@]12CCCN3CCc4c5ccccc5n(C(=O)C1)c4[C@H]23
Ekebergin	CC(C)CC(=O)O[C@H]1[C@H](C(C)(C)[C@H](CCC(=O)OC)[C@@]2(C)[C@H]3CC[C@@]4(C)[C@H](c5ccoc5)OC(=O)[C@H]([C@@]4(C3=C)O[C@@H]12)OC(=O)C)O
Elaeocyanidin	CC1(C)c2c(cc(c(c2O)OC)O)C2C(Cc3c(cc(cc3O2)O)O)O1
Elemanolide	CC(=C)C1C2C(C(CC1(C)C=C)OC(=O)C)C(=C)C(=O)O2
Ellagic acid	c1c2c3c4c(cc(c(c4oc2=O)O)O)c(=O)oc3c(c1O)O
Embelin	CCCCCCCCCCCC1=C(C(=O)C=C(C1=O)O)O
Ent-16-Kauren-18-oic-acid	C=C1C[C@]23CC[C@@H]4[C@](C)(CCC[C@@]4(C)[C@@H]3CC[C@H]1C2)CC(=O)O
Ent-16-Kauren-19-oic-acid	C=C1C[C@]23CCC4[C@@](C)(CCC[C@@]4(C)C(=O)O)C3CC[C@H]1C2
Entagenic-acid	CC1(C)CC[C@]2([C@@H](C1)C1=CCC3[C@@]4(C)CC[C@@H](C(C)(C)C4CC[C@@]3(C)[C@]1(C)[C@H]([C@H]2O)O)O)C(=O)O
Epicatechin	c1cc(c(cc1[C@@H]1[C@@H](Cc2c(cc(cc2O1)O)O)O)O)O
Epifriedelinol	CC3(C)C[C@H]4[C@]5(C)CC[C@]2(C)[C@H](CC[C@@]1(C)[C@H]2CC[C@H](O)[C@@H]1C)[C@@]5(C)CC[C@@]4(C)CC3
Epigallocatechin	c1c(cc(c(c1O)O)O)C1C(Cc2c(cc(cc2O1)O)O)O
Epigallocatechin gallate	c1c(cc(c(c1O)O)O)C1C(Cc2c(cc(cc2O1)O)O)OC(=O)c1cc(c(c(c1)O)O)O
Epimaslinic acid	CC1(C)CC[C@@]2(CC[C@]3(C)C(=CC[C@@H]4[C@@]5(C)C[C@H]([C@H](C(C)(C)[C@@H]5CC[C@@]34C)O)O)[C@@H]2C1)C(=O)O
Erectene	CC/C=C\C/C=C/CCCCCCCCCCCCCCC
Ergine	[CH][C@@]12Cc3c[nH]c4cccc(C1=C[C@H](CN2C)C(=O)N)c34

Ergoline	c1cc2[C@H]3CCCN[C@@H]3Cc3c[nH]c(c1)c23
Eroidictyol	C1C(OC2=CC(=CC(=C2C1=O)O)O)C3=CC(=C(C=C3)O)O
Erysotrine	COC1C=CC2=CCN3CCc4cc(c(cc4[C@]23C1)OC)OC
Erysovine	CO[C@H]1C=CC2=CCN3CCc4cc(c(cc4[C@]23C1)OC)OC
Erythraline	CO[C@H]1C=CC2=CCN3CCc4cc5c(cc4[C@]23C1)OCO5
Erythrophleine	C[C@H]1/C(=C/C(=O)OCCNC)/CCC2C1[C@H](CC1[C@]2(C)CCC[C@]1(C)C(=O)OC)O
Erythrophyllic-acid	CC(=CCCC(CO)[C@H]1CC[C@@]2(C)C3CCC4C(C)(C)C(=O)C=C[C@]54C[C@@]35C=C[C@]12C)C
Ethyl octanoate	CCOCCCCCCC(=O)C
Eucomnalin	COc1c(cc2c(C(=O)/C(=C/c3ccc(cc3)O)/CO2)c1O)O
Eucosterol	CCC(=O)[C@@H]1C[C@@H](C)[C@]2(CC(=O)[C@@]3(C)C4=C(CC[C@]23C)[C@@]2(C)CC[C@@H]([C@](C)(CO)C2CC4)O)O1
Eugenol	C=CCc1ccc(c(c1)OC)O
Falcarinol	CCCCCCC/C=C\C/C=C/C=C/[C@@H](C=C)O
Febuxostat	CC(C)COc1ccc(cc1C#N)c1nc(C)c(C(=O)O)s1
Fenchone	CC1(C)[C@@H]2CC[C@@](C)(C2)C1=O
Ferullic-acid	COc1cc(ccc1O)/C=C/C(=O)O
Flemichapparin C	COC1=CC2=C(C=C1)C3=C(C4=CC5=C(C=C4O3)OCO5)C(=O)O2
Formononetin	COC1=CC=C(C=C1)C2=COC3=C(C2=O)C=CC(=C3)O
Friedelin	C[C@H]1C(=O)CCC2[C@]1(C)CCC1[C@@]2(C)CC[C@@]2(C)[C@@H]3CC(C)(C)CC[C@]3(C)CC[C@]12C
Fructose	C([C@@H]1[C@@H](C([C@@](CO)(O)O1)O)O)O
Frutinone A	c1ccc2c(c1)c(=O)c1c(c3ccccc3oc1=O)o2
Fujikinetin	COC1=C(C=C2C(=C1)C(=O)C(=CO2)C3=CC4=C(C=C3)OCO4)O
Fumaric acid	C(=C\C(=O)O)/C(=O)O
Furanodiene	CC=1Cc2occ(C)c2CC=C(C)CCC=1C
Furanoeudesma-1-3-diene	CC2=CC=CC1Cc3occ(C)c3CC12
Gaboroquinone A	CC(=O)C1=C(C=C(C(=C1OC)C2=C3C(=C(C=C2CO)O)C(=O)C4=C(C3=O)C=CC=C4O)O)O
Gaboroquinone B	CC(=O)C1=C(C=C(C(=C1O)C2=C3C(=C(C=C2CO)O)C(=O)C4=C(C3=O)C=CC=C4O)O)OC
Galathamine	CN1CCC23C=CC(CC2OC4=C(C=CC(=C34)C1)OC)O
Gallic acid	c1c(cc(c(c1O)O)O)C(=O)O
Gallocatechin	c1c(cc(c(c1O)O)O)[C@@H]1[C@H](Cc2c(cc(cc2O1)O)O)O
Garcifuran B	COC1=CC(=CC(=C1O)O)C2=CC3=C(C=C2)OC=C3
Garcinia biflavonoid 1	C1=CC(=CC=C1C2C(C(=O)C3=C(C=C(C=C3O2)O)O)C4=C(C=C(C5=C4OC(C(C5=O)O)C6=CC=C(C=C6)O)O)O)O
Garcinia biflavonoid 2	C1=CC(=CC=C1C2C(C(=O)C3=C(C=C(C=C3O2)O)O)C4=C(C=C(C5=C4OC(C(C5=O)O)C6=CC(=C(C=C6)O)O)O)O)O

Geissoschizine	CC=C1CN2CCC3=C(C2CC1C(C=O)C(=O)OC)NC4=CC=CC=C34
Geissoschizol	CC=C1CN2CCC3=C(C2CC1CCO)NC4=CC=CC=C34
Gelsemicine	CC[C@@H]1[C@@H]2C[C@@H]3[C@]4(C[C@@H]([C@H]2CO3)N1)c1ccc(cc1N(C4=O)OC)OC
Genistein	c1cc(cc(c1)O)c1coc2cc(cc(c2c1=O)O)O
Gentiopicroside	C=C[C@@H]1C2=CCOC(=O)C2=CO[C@H]1OCC(C(C(C(CO)O)O)O)O
Geraniin	c1c(cc(c(c1O)O)O)OC(=O)[C@H]1C2C3[C@H](C(COC(=O)c4cc(c(c(c4c4c(cc(c(c4O)O)O)C(=O)O3)O)O)O)O1)OC(=O)C1=CC(=O)[C@]3(C(C1c1c(cc(c(c1O3)O)O)C(=O)O2)(O)O)O
Geraniol	CC(=CCC/C(=C/CO)/C)C
Germacrene B	CC(=C1CC/C(=C/CC/C(=C/C1)/C)/C)C
Germacrene D	CC(C)C1/C=C\C(=C)CC/C=C(/C)\CC1
Gingerdione	CCCCCC(=O)CC(=O)CCC1=CC(=C(C=C1)O)OC
Gitogenin	C[C@@H]1CC[C@@]2([C@@H](C)[C@H]3[C@H](C[C@H]4[C@@H]5CC[C@H]6C[C@H]([C@@H](C[C@]6(C)[C@H]5CC[C@]34C)O)O)O2)OC1
Glaucolide A	CC(=O)OC1=C2[C@@H](C[C@@](C)(C(=O)CC[C@@]3(C)[C@@H]([C@@H]2OC1=O)O3)OC(=O)C)O
Glaziovine	CN1CCc2cc(c(c3c2[C@@H]1CC13C=CC(=O)C=C1)O)OC
Glucose	C(C1[C@H]([C@@H](C([C@H](O)O1)O)O)O)O
Glycyrrhizin	CC1(C)C2CC[C@]3(C)C(C(=O)C=C4[C@@H]5C[C@](C)(CC[C@]5(C)CC[C@@]34C)C(=O)O)[C@@]2(C)CC[C@@H]1OC1C(C(C(C(C(=O)O)O1)O)O)OC1C(C(C(C(C(=O)O)O1)O)O)O
Gnidicin	CC1C(C2(C3C4C1(C5C=C(C(=O)C5(C(C6(C4O6)CO)O)O)C)OC(O3)(O2)C7=CC=CC=C7)C(=C)C)OC(=O)C=CC8=CC=CC=C8
Gnidilatin	CCCCCCCCCC12OC3C4C5C(O5)(C(C6(C(C4(O1)C(C(C3(O2)C(=C)C)OC(=O)C7=CC=CC=C7)C)C=C(C6=O)C)O)O)CO
Gniditrin	CCCC=CC=CC=CC(=O)OC1C(C23C4C=C(C(=O)C4(C(C5(C(C2C6C1(OC(O6)(O3)C7=CC=CC=C7)C(=C)C)O5)CO)O)O)C)C
Gomphoside	O=C1C=C(CO1)[C@H]7CC[C@@]3(O)C7CCC2C4C[C@H]5O[C@@]6(O)[C@H](O)C[C@@H](C)O[C@H]6O[C@@H]5C[C@@H]4CCC23
Gossypetin	C1=CC(=C(C=C1C2=C(C(=O)C3=C(O2)C(=C(C=C3O)O)O)O)O)O
Grayanotoxin I	CC(=O)O[C@@H]1[C@H]2CCC3[C@](C)([C@@H]4C[C@@H](C(C)(C)[C@]4([C@@H](C[C@@]13CC2(C)O)O)O)O)O
Grevillol	CCCCCCCCCCCCCc1cc(cc(c1)O)O
Guaijaverin	Oc1ccc(cc1O)C=3Oc4cc(O)cc(O)c4C(=O)C=3OC2OC[C@H](O)[C@H](O)[C@H]2O
Harman	Cc1c2c(ccn1)c1ccccc1[nH]2
Harpagide	C[C@@]1(C[C@H]([C@@]2(C=CO[C@H]([C@H]12)OCC(C(C(C(C=O)O)O)O)O)O)O)O
Harpagoside	C[C@@]1(C[C@H]([C@@]2(C=CO[C@H]([C@H]12)OCC(C(C(C(C=O)O)O)O)O)O)O)OC(=O)/C=C\c1ccccc1
Harunganin	CC1=C(C2=CC3=C(C(=CC(=O)C3(CC=C(C)C)CC=C(C)C)O)C(=C2C(=C1)O)O)CC=C(C)C

Harunganol B	C/C(C)=C\Cc2c1Cc3c(C(=O)c1c(O)c(C\C=C(/C)C)c2C)c(O)cc(O)c3C\C=C(/C)C
Harunmadagascarin A	CC1=CC2=CC3=C(C(=C2C(=C1)O)O)C(=O)C4=C(C3(CC=C(C)C)CC=C(C)C)OC(C=C4)(C)C
Harunmadagascarin B	C/C(C)=C\CC2(C\C=C(/C)C)C=4OC(C)(C)C=CC=4C(=O)c1c(O)c3c(cc12)c(C\C=C(/C)C)c(C)cc3O
Hautriwaic acid	C[C@@H]1CC[C@@]2(CO)C(=CCC[C@@H]2[C@@]1(C)CCc1ccoc1)C(=O)O
Heamanthamine	C1=CC23c4cc5c(cc4CN(C[C@H]3O)[C@H]2C[C@@H]1ON)OCO5
Heamanthidine	C1=CC23c4cc5c(cc4C(N(C[C@H]3O)[C@H]2C[C@@H]1ON)O)OCO5
Hederagenin	CC1(C)CC[C@@]2(CC[C@]3(C)C(=CCC4[C@@]5(C)CC[C@@H]([C@@](C)(CO)C5CC[C@@]34C)O)C2C1)C(=O)O
Helichrysetin	COc1cc(cc(c1C(=O)/C=C/C1C=CC(C=C1)O)O)O
Helinudichromene quinone	CC(=CCCC1(C)C=Cc2c(c(=O)c(c(C(=O)C(C)C)o2)OC)O1)C
Heliosupine	C/C=C(/C)\C(=O)O[C@H]1CCN2CC=C(COC(=O)[C@@]([C@@H](C)O)(C(C)(C)O)O)C12
hellebrigenin	CC12CCC3C(C1(CCC2C4=COC(=O)C=C4)O)CCC5(C3(CCC(C5)O)C=O)O
Hellebrigenin-3-acetate	CC(=O)OC1CCC2(C3CCC4(C(CCC4(C3CCC2(C1)O)O)C5=COC(=O)C=C5)C)C=O
Henningsiine	C[C@@H]1[C@@H]2CN3CC[C@@]45c6ccccc6N(C(=O)CO)[C@H]5C(=CO1)[C@H]2C[C@H]34
Hesperidin	COc1ccc(cc1O)[C@@H]1CC(=O)c2c(cc(cc2O1)OCC(C(C(C(C=O)O)O)O)O)O
Hesperitin	COC1=C(C=C(C=C1)C2CC(=O)C3=C(C=C(C=C3O2)O)O)O
Hibiscetin	C1=C(C=C(C(=C1O)O)O)C2=C(C(=O)C3=C(O2)C(=C(C=C3O)O)O)O
Hibiscin	C1C(C(C(C(O1)OC2C(C(C(OC2OC3=C([O+]=C4C=C(C=C(C4=C3)O)O)C5=CC(=C(C(=C5)O)O)O)CO)O)O)O)O)O
Hibiscitrin	C1=C(C=C(C(=C1O)O)O)C2=C(C(=O)C3=C(O2)C(=C(C=C3O)O)O)OC4C(C(C(C(O4)CO)O)O)O
Hibiscus acid	C(C(=O)O)C(C(C(=O)O)O)(C(=O)O)O
Hippeastrine	CN1CCC2=C[C@@H]([C@@H]3[C@@H](c4cc5c(cc4C(=O)O3)OCO5)[C@H]12)O
Hircinol	COc1cc(cc2CCc3cccc(c3c12)O)O
Hispanolone	C[C@@H]1C(=O)CC2C(C)(C)CCC[C@]2(C)[C@]1(CCc1ccoc1)O
Histamine	C(CN)c1c[nH]cn1
Hordenine	CN(C)CCc1ccc(cc1)O
Humulene	C/C/1=C\CC/C(=C/CC(C)(C)/C=C/C1)/C
Hydroxyvernolide	C=C1C2C(CC34C(O3)CCC(=CC2OC1=O)COC4O)OC(=O)C(=C)CO
Hyoscine	CN1[C@@H]2CC(CC1[C@@H]1[C@H]2O1)OC(=O)[C@H](CO)c1ccccc1
Hyoscyamine	CN1C2CCC1CC(C2)OC(=O)[C@H](CO)c1ccccc1
Hyperforin	CC(=CCCC1(C)C(CC=C(C)C)CC2(CC=C(C)C)C(=O)C(=C(C1(C(=O)C(C)C)C2=O)O)CC=C(C)C)C
Hypericin	CC1=CC(=C2C3=C1C4=C5C(=C(C=C4C)O)C(=O)C6=C(C=C(C7=C6C5=C3C8=C7C(=CC(=C8C2=O)O)O)O)O)O
Hyperin	C1=CC(=C(C=C1C2=C(C(=O)C3=C(C=C(C=C3O2)O)O)OC4C(C(C(C(O4)CO)O)O)O)O)[O-]
Hyperoside	c1cc(c(cc1c1c(c(=O)c2c(cc(cc2o1)O)O)OCC(C(C(C(C=O)O)O)O)O)O)O
hypoxoside	C1=CC(=C(C=C1C=CCC#CC2=CC(=C(C=C2)OC3C(C(C(C(O3)CO)O)O)O)O)OC4C(C(C(C(O4)CO)O)O)O

Ibogaine	CCC1CC2CC3C1N(C2)CCC4=C3NC5=C4C=C(C=C5)OC
Ibogamine	CC[C@H]1C[C@H]2C[C@@H]3[C@H]1N(C2)CCC4=C3NC5=CC=CC=C45
Iboxygaine	C[C@@H]([C@@H]1C[C@@H]2C[C@H]3c4c(CCN(C2)[C@@H]13)c1cc(ccc1[nH]4)OC)O
Ibozol	CC(C)[C@]1(CCC2=C(C1)[C@H](CC1C(C)(C)CCC[C@]21C)O)O
Icterogenin	CC=C(C)C(=O)OC1CC(CC2C1(CCC3(C2=CCC4C3(CCC5C4(CCC(=O)C5(C)CO)C)C)C)C(=O)O)(C)C
Ilexoside A	C[C@@H]1CC[C@@]2(CC[C@]3(C)C(=CCC4[C@@]5(C)CC[C@@H](C(C)(C)C5CC[C@@]34C)O[C@H]3[C@@H]([C@H]([C@@H](CO)O3)O)O)C2=C1C)C(=O)O
Indole-3-acetyl aspartic acid	c1ccc2c(c1)c(CC(=N[C@@H](CC(=O)O)C(=O)O)O)c[nH]2
Ingenol	CC1=CC23[C@H](C)CC4C(C(C=C(CO)[C@H]([C@@]3([C@H]1O)O)O)C2=O)C4(C)C
Insularine	CN1CCc2cc(c3c4c2[C@H]1Cc1ccc(cc1)Oc1c2c(CCN(C)[C@@H]2Cc2ccc(c(c2)CO3)O4)cc(c1OC)OC)OC
Integerrimine	CC=C1CC(C(C(=O)OCC2=CCN3C2C(CC3)OC1=O)(C)O)C
Integriquinoline	Oc1ccc2c(c1)C(=CC(=O)N2C)OC
Ipsdienone	C=CC(=C)CC(=O)C=C(C)C
Isocryptolepine	CN1C=C2C3=CC=CC=C3N=C2C4=CC=CC=C41
Isoeugenitol	Cc1cc(=O)c2c(cc(c(C)c2o1)O)O
Isofucosterol	C/C=C(/CC[C@@H](C)[C@H]1CC[C@H]2[C@@H]3CC=C4C[C@H](CC[C@]4(C)[C@H]3CC[C@]12C)O) \ C(C)C
Isofuranogermacrene	CC1=COC2=C1CC(C(C2)(C)C=C)C(=C)C
Isokigelinol	Oc3cc4C(=O)C2=CC1=C(C)CCC1=C(C)C2(C)C(=O)c4cc3
Isomethone	CC(C)[C@H]1CC[C@@H](C)C(C)C1=O
Isoorientin	c1cc(c(cc1c1cc(=O)c2c(cc(c(c2O)[C@H]2[C@@H]([C@H]([C@@H]([C@@H](CO)O2)O)O)O)O)o1)O)O
Isopimpinellin	COc1c2ccc(=O)oc2c(c2c1cco2)OC
Isopinnatal	CC12CCC3C1C=C4C(=O)C5=C(C=CC(=C5)O)C(=O)C4(C3(C)C=O)O2
Isopolygodial	CC1(C)CCC[C@]2(C)[C@H](C=O)C(=CC[C@@H]12)C=O
Isoquercetin	C1=CC(=C(C=C1C2=C(C(=O)C3=C(C=C(C=C3O2)O)O)OC4C(C(C(C(O4)CO)O)O)O)O)O
Isoreserpiline	C[C@H]1[C@@H]2CN3CCc4c5cc(c(cc5[nH]c4[C@@H]3C[C@@H]2C(=CO1)C(=O)OC)OC)OC
Isoreserpine	COC1C(CC2CN3CCC4=C(C3CC2C1C(=O)OC)NC5=C4C=CC(=C5)OC)OC(=O)C6=CC(=C(C(=C6)OC)OC)OC
Isorhamnetin	COC1=C(C=CC(=C1)C2=C(C(=O)C3=C(C=C(C=C3O2)O)O)O)O
Isosakuranetin	COc1ccc(cc1)[C@@H]1CC(=O)c2c(cc(cc2O1)O)O
Isovanillin	COC1=C(C=C(C=C1)C=O)O
Isovitexin	C1=CC(=CC=C1C2=CC(=O)C3=C(C(=C(C=C3O2)O)C4C(C(C(C(O4)CO)O)O)O)O)O
Ivangustin	CC1=C2CC3C(=C)C(=O)O[C@@H]3C[C@@]2(C)[C@@H](CC1)O
Jiofuran	C1=C2[C@H]([C@@H]([C@@H](C2=CO1)O)O)CCO
Jioglutolide	CC1(CC(C2C1COC(=O)C2)O)O

Kaempferol	c1cc(ccc1c1c(c(=O)c2c(cc(cc2o1)O)O)O)O
kaempferol-3-glucoside	C1=CC(=CC=C1C2=C(C(=O)C3=C(O2)C=C(C=C3[O-])O)OC4C(C(C(C(O4)CO)O)O)O)O
kaempferol-3-o-(6'-malonyl-glucoside)	C1=CC(=CC=C1C2=C(C(=O)C3=C(C=C(C=C3O2)O)O)OC4C(C(C(C(O4)COC(=O)CC(=O)O)O)O)O)O
kaempferol-3-O-rhamnodiglucoside	OC6C(O)C(O)C(CO)OC6OC1C(O)C(OC(C)C1O)OCC5OC(OC=2C(=O)c4c(O)cc(O)cc4OC=2c3ccc(O)cc3)C(O)C(O)C5O
Kauran-16α-ol	CC4(C)CCCC1(C)C4CCC32CC(CCC12)C(C)(O)C3
Kigelin	C[C@@H]1Cc2cc(c(c(c2C(=O)O1)O)OC)OC
Kigelinol	CC1=C2C=C3C(=O)C4=C(C=C(C=C4)O)C(=O)C3(C(=C2CC1)C)O
Kigelinone	CC(C1=CC2=C(O1)C(=O)C3=C(C2=O)C=CC=C3O)O
Knipholone	CC(=O)c1c(cc(c(c2c(cc(c3c2C(=O)c2cccc(c2C3=O)O)O)CO)c1O)O)CO
Kokusaginine	COC1=C(C=C2C(=C1)C(=C3C=COC3=N2)OC)OC
Kolaflavanone	COC1=C(C=C(C=C1)C2C(C(=O)C3=C(O2)C(=C(C=C3O)O)C4C(OC5=CC(=CC(=C5C4=O)O)O)C6=CC=C(C=C6)O)O)O
Kolanone	CC(=CCCC(=CCC1(C(=O)C(=C(C(=C(C2=CC=CC=C2)O)C1=O)O)CC=C(C)C)CC=C(C)C)C)C
Kraussianin	CCCCCCC(=O)O[C@H]1[C@@H](C)[C@@H]2[C@H](C)CCCCCC[C@H](C34[C@@H](C)[C@@]56[C@@H](C7[C@](CO)([C@H]([C@]1([C@@H]25)O)O)O7)[C@@H]1C3(C(=C)C)OC4(O1)O6)O
Lactucin	CC1=C2C(=O)C=C(CO)[C@@H]2[C@@H]2C(C(=C)C(=O)O2)[C@H](C1)O
Lanceotoxin A	C[C@@H]([C@@H]([C@H]([C@H](C(=O)O[C@H]1CC[C@]2(CO)C3CC[C@]4(C)[C@H](CC[C@@]4(C3CC[C@@]2(C1)OC(=O)C)O)c1ccc(=O)oc1)O)O)O)O
Lanceotoxin B	CC1C(C(C(C(O1)O[C@H]1CC[C@]2(CO)C3CC[C@]4(C)[C@H](CC[C@@]4(C3CC[C@@]2(C1)OC(=O)C)O)c1ccc(=O)oc1)O)O)O
Lantadene A	C/C=C(/C)\C(=O)O[C@@H]1CC(C)(C)C[C@H]2C3=CC[C@@H]4[C@@]5(C)CCC(=O)C(C)(C)[C@@H]5CC[C@@]4(C)[C@]3(C)CC[C@@]12C(=O)O
Lantadene B	CC(=CC(=O)OC1CC(CC2C1(CCC3(C2=CCC4C3(CCC5C4(CCC(=O)C5(C)C)C)C)C)C(=O)O)(C)C)C
Lapachol	CC(=CCC1=C(C(=O)c2ccccc2C1=O)O)C
Lasiosperman	CC(CCCc1ccoc1)Cc1cc(C)co1
Lauric acid	CCCCCCCCCCCC(=O)O
L-Djenkolic acid	C([C@@H](C(=O)O)N)SCSCC(C(=O)O)N
Lemmatoxin	CC1(CCC2(CCC3(C(=CCC4C3(CCC5C4(CCC(C5(C)C)OC6C(C(C(C(O6)CO)OC7C(C(C(C(O7)CO)O)O)O)OC8C(C(C(C(O8)CO)O)O)O)O)C)C)C2C1)C)C(=O)O)C
Leucocyanidin	C1=CC(=C(C=C1C2C(C(C3=C(C=C(C=C3O2)O)O)O)O)O)O
Leurosidine	CCC1(CC2CC(C3=C(CCN(C2)C1)C4=CC=CC=C4N3)(C5=C(C=C6C(=C5)C78CCN9C7C(C=CC9)(C(C(C8N6C)(C(=O)OC)O)OC(=O)C)CC)OC)C(=O)OC)O
Leurosine	CCC12CN3CCC4=C(C(CC(C3)C1O2)(C5=C(C=C6C(=C5)C78CCN9C7C(C=CC9)(C(C(C8N6C)(C(=O)OC)O)OC(=O)C)CC)OC)C(=O)OC)NC1=CC=CC=C41

Lignoceric acid	CCCCCCCCCCCCCCCCCCCCCCCC(=O)O
Ligustroside	C/C=C/1\[C@H](CC(=O)OCCc2ccc(cc2)O)C(=CO[C@H]1OC1C(C(C(C(CO)O1)O)O)O)C(=O)OC
Limonene	C=C(C)C1CC=C(C)CC1
Linalool	C=C[C@](C)(CCC=C(C)C)O
Linamarin	CC(C)(C#N)OC1C(C(C(C(CO)O1)O)O)O
Lindestrene	C=C1CC=CC2(C)Cc3occ(C)c3CC12
Liquiritin	c1cc(ccc1[C@@H]1CC(=O)c2ccc(cc2O1)O)OCC(C(C(C(C=O)O)O)O)O
Lithospermic acid	C1=CC(=C(C=C1CC(C(=O)O)OC(=O)C=CC2=C3C(C(OC3=C(C=C2)O)C4=CC(=C(C=C4)O)O)C(=O)O)O)O
L-Mimosine	c1cn(C[C@@H](C(=O)O)N)cc(c1=O)O
Lochnerine	CC=C1CN2C3CC1C(C2CC4=C3NC5=C4C=C(C=C5)OC)CO
Loganin	CC1C(CC2C1C(OC=C2C(=O)OC)OC3C(C(C(C(O3)CO)O)O)O)O
Longistylin A	CC(=CCC1=C(C=C(C=C1OC)C=CC2=CC=CC=C2)O)C
Longistylin C	CC(=CCC1=C(C=C(C=C1C=CC2=CC=CC=C2)O)OC)C
L-Quebrachitol	COC1C(C(C(C(C1O)O)O)O)O
Lupanine	C1CCN2C[C@@H]3C[C@H](CN4[C@@H]3CCCC4=O)[C@@H]2C1
Lupeol	CC(=C)C1CCC2(C1C3CCC4C5(CCC(C(C5CCC4(C3(CC2)C)C)(C)C)O)C)C
Luteolin	C1=CC(=C(C=C1C2=CC(=O)C3=C(C=C(C=C3O2)O)O)O)O
Luteolin-4-rutinoside	Oc1cc(O)cc2OC(CC(=O)c12)c5ccc(OC4O[C@H](COC3C[C@H](C)[C@@H](O)[C@H](O)[C@H]3O)[C@@H](O)[C@H](O)[C@H]4O)c(O)c5
Luteolin-7-glucoside	C1=CC(=C(C=C1C2=CC(=O)C3=C(C=C(C=C3O2)OC4C(C(C(C(O4)CO)O)O)O)O)O)O
Luteolin-7-rutinoside	CC1C(C(C(C(O1)OCC2C(C(C(C(O2)OC3=CC(=C4C(=C3)OC(=CC4=O)C5=CC(=C(C=C5)O)O)O)O)O)O)O)O)O
Lycorine	C1CN2Cc3cc4c(cc3[C@H]3[C@H]2C1=C[C@@H]([C@H]3O)O)OCO4
Macowine	COc1cc2c(cc1O)CN1CC[C@@]32C=C[C@@H](C[C@@H]13)O
Macrozamin	C/[N+](=N\CO[C@H]1C([C@H]([C@@H](C(CO[C@H]2[C@@H](C([C@@H](CO2)O)O)O)O1)O)O)O)/[O-]
Madecassic acid	C[C@@H]1CCC2(CC[C@]3(C)C(=CCC4[C@@]5(C)C[C@H]([C@@H]([C@@](C)(CO)C5[C@@H](C[C@@]34C)O)O)O)[C@@H]2[C@H]1C)C(=O)O
Maleic acid	C(=C\C(=O)O)\C(=O)O
Malic acid	C(C(C(=O)O)O)C(=O)O
Malinic acid	C(=C(\CC(=O)O)/C(=O)O)\C(=O)O
Malvidin	COC1=CC(=CC(=C1O)OC)C2=C(C=C3C(=CC(=CC3=[O+]2)O)O)O
Mangiferin	c1c2c(cc(c1O)O)oc1cc(c(c(c1c2=O)O)O)OCC(C(C(C(CO)O)O)O)O)O
Mannitol	C(C(C(C(C(CO)O)O)O)O)O
Marasmicin	CSCSS(=O)CSC

Marrubiin	C[C@@H]1C[C@@H]2[C@H]3[C@](C)(CCC[C@]3(C)[C@]1(CCC1C=COC1)O)C(=O)O2
Maslinic acid	CC1(C)CC[C@@]2(CC[C@]3(C)C(=CC[C@@H]4[C@@]5(C)C[C@H]([C@@H](C(C)(C)[C@@H]5CC[C@@]34C)O)O)[C@@H]2C1)C(=O)O
Medicagol	C1OC2=C(O1)C=C3C(=C2)C4=C(O3)C5=C(C=C(C=C5)O)OC4=O
Melianol	CC1(C)[C@@H]2CC=C3[C@H](CC[C@@]4(C)[C@@H](CC[C@]34C)[C@@H]3C[C@H]([C@H]4C(C)(C)O4)O[C@H]3O)[C@@]2(C)CC[C@@H]1O
Melianthugenin	CC(=O)O[C@H]1C[C@]2(CO)C3CC[C@]4(C)C[C@H](C[C@@]4([C@@H]3CC[C@@]2(C[C@H]1OC(=O)C)OC(=O)C)O)c1ccc(=O)oc1
Melianthusigenin	CC(=O)OCC12C7CC6CC1(CCC3C2CCC4(C)C(CCC34O)c5ccc(=O)oc5)OC(C)(O6)O7
Meliatoxin A1	CCC(C)C(=O)OC1C2(C)C3CC(C4(C)C(C(=O)CC5(C)C(CC6C45O6)c4ccoc4)C3(CO1)C(C(C2OC(=O)C)OC(=O)C)O)O
Meliatoxin A2	CC(C)C(=O)OC1C2(C)C3CC(C4(C)C(C(=O)CC5(C)C(CC6C45O6)c4ccoc4)C3(CO1)C(C(C2OC(=O)C)OC(=O)C)O)O
Meliatoxin B1	CCC(C)C(=O)OC1C2(C)C3CC(C4(C)C5CC(=O)C(c6ccoc6)C5(C)CC(=O)C4C3(CO1)C(C(C2OC(=O)C)OC(=O)C)O)O
Meliatoxin B2	CC(C)C(=O)OC1C2(C)C3CC(C4(C)C5CC(=O)C(c6ccoc6)C5(C)CC(=O)C4C3(CO1)C(C(C2OC(=O)C)OC(=O)C)O)O
Melissic acid	CCCCCCCCCCCCCCCCCCCCCCCCCCCCCC(=O)O
Mellitoxin	C=C(C)C1([C@@H]2C(=O)O[C@H]1[C@H]([C@@]1(C)[C@@]3(CO3)[C@H]3[C@@H]([C@@]21O)O3)O)O
Menthol	CC(C)[C@@H]1CC[C@@H](C)C[C@H]1O
Menthone	CC(C)[C@@H]1CC[C@@H](C)CC1=O
Mesembranol	CN1CCC2(CCC(CC12)O)c1ccc(c(c1)OC)OC
Mesembrenone	CN1CCC2(C=CC(=O)CC12)c1ccc(c(c1)OC)OC
Mesembrine	CN1CCC2(CCC(=O)CC12)c1ccc(c(c1)OC)OC
Mesquitol	c1cc(c(cc1[C@H]1[C@@H](Cc2ccc(c(c2O1)O)O)O)O)O
Methyl salicylate	COC(=O)c1ccccc1O
Methylasparagine	CNC(=O)CC(C(=O)O)N
Methylazoxymethanol	C/[N+](=N/CO)/[O-]
Methylnonyl ketone	CCCCCCCCCCOC
Monofluoroacetic acid	C(C(=O)O)F
Montanine	CO[C@H]1C=C2[C@H]3CN(Cc4cc5c(cc34)OCO5)[C@H]2C[C@@H]1O
Moringine	C1=CC=C(C=C1)CN
Moringyne	CC1=CC=CC(C)=C1C(=O)OC1OC(CO)C(O)C(O)C1O
Mucronine D	CCC(C)C1C(=O)N/C=C/c2ccc(cc2)OC2CCN(C2C(=O)N1)C(=O)C(CC(C)C)NC(=O)C(Cc1ccccc1)N(C)C
Mukaadial	CC1(C)CCC[C@@]2(C)[C@H]1[C@H](C=C(C=O)[C@@]2(C=O)O)O
Muzigadial	CC1CCC2(C(C1=C)CC=C(C2(C=O)O)C=O)C
Myrcene	C=CC(=C)CCC=C(C)C

Myricetin	c1cc(c(c(c1c1coc2cc(cc(c2c1=O)O)O)O)O)O
Myristic acid	CCCCCCCCCCCCCC(=O)O
Naringenin	c1cc(ccc1C1CC(=O)c2c(cc(cc2O1)O)O)O
Nauclefidine	O=CC3=CC=C2c4nc1ccccc1c4CCN2C3=O
Naucletine	CC(=O)C1=C2C=C3C4=C(CCN3C(=O)C2=CN=C1)C5=CC=CC=C5N4
Neocryptolepine	CN1C2=CC=CC=C2C=C3C1=NC4=CC=CC=C43
Neral	CC(=CCC/C(=C\C=O)/C)C
N-Hentriacontane	CCCCCCCCCCCCCCCCCCCCCCCCCCCCCCC
Niazimicin	CCOC(=S)NCC1=CC=C(C=C1)OC2C(C(C(C(O2)C)O)O)O
Niazinin A	CC1C(C(C(C(O1)OC2=CC=C(C=C2)CNC(=S)OC)O)O)O
Niazinin B	COC(=S)NCC1=CC=C(OC2OC(C)C(O)C(O)C2O)C=C1
Nicotine	CN1CCC[C@H]1c1cccnc1
N-Methylconiine	CCC[C@H]1CCCCN1C
N-methylflindersine	CC1(C)C=Cc2c(c3ccccc3n(C)c2=O)O1
N-Nonacosane	CCCCCCCCCCCCCCCCCCCCCCCCCCCCC
Norajmaline	CCC1C2CC3C4C5(CC(C2C5O)N3C1O)C6=CC=CC=C6N4
Norharman	c1ccc2c(c1)c1ccncc1[nH]2
Norhyoscine	c1ccc(cc1)[C@@H](CO)C(=O)OC1CC2C3C(C(C1)N2)O3
Nothofagin	c1cc(ccc1CCC1c2c(cc(cc2O[H-]O1)O)O)O
N-trans-Feruloyltyramine	COc1cc(ccc1O)/C=C/C(=O)NCCc1ccc(cc1)O
N-trans-Tetracosyl ferulate	Oc1ccc(cc1OC)/C=C/C(=O)OCCCCCCCCCCCCCCCCCCCCCCCC
Nudicauline	CCN1C[C@@]2(CCC(C34[C@@H]5C[C@@H]6[C@H](C[C@@]([C@H]5[C@H]6OC(=O)C)(C([C@@H]([C@H]23)OC)[C@H]14)O)OC)OC)COC(=O)c1ccccc1N1C(=O)C[C@H](C)C1=O
Nymania 1	CC(CC)C(O)C(=O)O[C@H]2[C@H](OC=O)C(C(=C)[C@@]3(O)C(=O)C[C@@H](c1ccoc1)[C@]23C)[C@]4(C)[C@@H](CC(=O)OC)[C@]5(C)COC(O)(C[C@@H]4OC(C)=O)O5
Obebioside	CC1C(C(C(C(O1)O[C@H]1CC[C@@]2(C)[C@H](CCC3C2CC[C@]2(C)[C@@H](C4=CC(=O)OC4)[C@H](C[C@]32O)OC(=O)CC(=O)C(=O)O)C1)O)OC1C(C(C(C(CO)O1)O)O)O)O
Ocubullenone	C=CCC12CC34C(=CC(=C)C23CC1c1cc(c2c(c1)OCO2)OC)OCO4
Oleacein	C/C=C(/C=O)\C(CC=O)CC(=O)OCCc1ccc(c(c1)O)O
Oleandrin	CC1C(C(CC(O1)O[C@H]1CC[C@@]2(C)[C@H](CCC3C2CC[C@]2(C)[C@@H](C4=CC(=O)OC4)[C@H](C[C@]32O)OC(=O)C)C1)OC)O
Oleanoglycotoxin	CC1(C)CC[C@@]2(CC[C@]3(C)C(=CCC4[C@@]5(C)CC[C@@H](C(C)(C)C5CC[C@@]34C)OC3C(C(C(C(CO)O3)O)OC3C(C(C(C(CO)O3)O)O)O)OC3C(C(C(C(CO)O3)O)O)O)C2C1)C(=O)O
Oleanolic acid	CC1(C)CCC2(CC[C@]3(C)C(=CCC4[C@@]5(C)CC[C@@H](C(C)(C)[C@@H]5CC[C@@]34C)O)[C@@H]2C1)C(=O)O

Oleanolic-3-acetate	CC(=O)OC1CCC2(C)C(CCC3(C)C2CC=C2C4CC(C)(C)CCC4(CCC32C)C(=O)O)C1(C)C
Oleuropein	C/C=C/1\[C@H](CC(=O)OCCc2ccc(c(c2)O)O)C(=CC[C@H]1OCC(C(C(C(C=O)O)O)O)O)C(=O)OC
Olivil	COC1=C(C=CC(=C1)CC2(COC(C2CO)C3=CC(=C(C=C3)O)OC)O)O
Orbicuside A	C[C@H]1C[C@H]2[C@H]3[C@@H](O1)O[C@@H]1CC4C[C@H]5[C@@]6(C(CC(=O)[C@]7(C)[C@H](CC[C@]67O)c6ccc(=O)oc6)[C@@]4(C)C[C@]1(O2)O3)O5
Orientin	c1cc(c(cc1c1cc(=O)c2c(cc(c(c2o1)[C@H]1[C@@H]([C@H]([C@@H]([C@@H](CO)O1)O)O)O)O)O)O)O
Osmitopsin	CC1=C2[C@@H]3[C@H](CC[C@@H](C)[C@@]2(C)CC1)C(=C)C(=O)O3
Ouabain	C[C@@]12C[C@H](C3[C@@H](CC[C@@]4(C[C@H](CC([C@]34CO)O)OCC(C(C(C(C=O)O)O)O)O)O)[C@]2(CC[C@@H]1C1=CC(=O)OC1)O)O
Ouratea-proanthocyanidin A	COc1c(cc(cc1O)[C@@H]1[C@@H](Cc2c(cc(c(C3c4c(cc(cc4O[C@H](c4ccc(cc4)O)[C@@H]3O)O)O)c2O1)O)O)O)O
Oxalic acid	C(=O)(C(=O)O)O
Oxyavicine	Cn1c2c(ccc3cc4c(cc23)OCO4)c2cc3c(cc2c1=O)OCO3
Oxychelerythrine	Cn1c2c(ccc3cc4c(cc23)OCO4)c2ccc(c(c2c1=O)OC)OC
Oxypurinol	c1c2c([nH]c(=O)[nH]c2=O)[nH]n1
P57	C/C=C(\C)/C(=O)O[C@H]1CC2C(CC=C3C[C@H](CC[C@]23C)O[C@H]2C[C@@H]([C@@H]([C@@H](C)O2)O[C@H]2C[C@@H]([C@@H]([C@@H](C)O2)O[C@H]2[C@@H]([C@H]([C@@H]([C@@H](C)O2)O)OC)O)OC)OC)[C@]2(CC[C@H](C(=O)C)[C@@]12C)O
Palmitic acid	CCCCCCCCCCCCCCCC(=O)O
Palustrine	CC[C@@H]([C@@H]1CC=C[C@@H]2CC(=O)NCCCCNCCCN12)O
Parquin	CC(C)CC(=O)OC1C(OC2C[C@H]([C@H]3CC[C@@]45C[C@@H](CCC4[C@]3(C)C2)C(=C)C5O)C(=O)O)OC(CO)C(C1(CO)O)OC1C(C(=O)C(=O)O1)OCO
p-Coumaric acid	C1=CC(=CC=C1C=CC(=O)O)O
p-Cymene	CC(C)c1ccc(C)cc1
Peddiea Factor A1	CCC/C=C/C=C/C=C/C12O[C@@H]3[C@@H]4[C@H]5[C@@](CO)([C@H]([C@]6(C(C=C(C)C6=O)[C@]4(C(C)[C@H]([C@]3(C(=C)C)O1)O)O2)O)O)O5
Pelletierine	CC(=O)C[C@H]1CCCCN1
Pentacosane	CCCCCCCCCCCCCCCCCCCCCCCCC
Pentalongin	c1ccc2c(c1)C(=O)C1=C(COC=C1)C2=O
Pentatriacontane	CCCCCCCCCCCCCCCCCCCCCCCCCCCCCCCCCCC
Perforatin A	Cc1cc(=O)c2c(cc3c(C=CC(C)(C)O3)c2o1)OC
Persin	CCCCC/C=C\C/C=C\CCCCCCCC(=O)C[C@H](COC(=O)C)O
Pescaproside A	CCCCCCCCCCCC(=O)OC1C(C(C(OC1OC2C(OC(C(C2O)O)OC3C(C(C(OC3OC(CCCCC)CCCCCCCCCC(=O)OC)C)O)O)C)C)OC4C(C(C(C(O4)C)O)O)O)OC5C(C(C(C(O5)C)O)O)O

Pescaproside B	CCCCCCCCCCCC(=O)O[C@@H]1[C@@H]([C@H]([C@H](C)O[C@H]1O[C@H]1[C@H](C)O[C@H]([C@@H]([C@@H]1O)O)O[C@@H]1[C@H]([C@H]([C@@H](C)O[C@H]1O[C@@H](CCCCC)CCCCCCCCCC(=O)OC)O)O)O[C@H]1[C@@H]([C@@H]([C@H]([C@H](C)O1)O)O)O)O[C@H]1[C@@H]([C@@H]([C@H]([C@H](C)O1)O)O)O
Pescaproside E	CCCCCCCCCCCC(=O)O[C@@H]1[C@@H]([C@H]([C@H](C)O[C@H]1O[C@H]1[C@H](C)O[C@H]([C@@H]([C@@H]1O)O)O[C@@H]1[C@H]([C@H]([C@@H](C)O[C@H]1O[C@@H](CCCCC)CCCCCCCCCC(=O)OC)O)O)O[C@H]1[C@@H]([C@@H]([C@H]([C@H](C)O1)O)O)O)O[C@H]1[C@@H]([C@@H]([C@H]([C@H](C)O1)O)O)O
Petunidine	COC1=CC(=CC(=C1O)O)C2=C(C=C3C(=CC(=CC3=[O+]2)O)O)O
Phytic acid	C1(C(C(C(C(C1OP(=O)(O)O)OP(=O)(O)O)OP(=O)(O)O)OP(=O)(O)O)OP(=O)(O)O)OP(=O)(O)O
Picrinine	CC=C1CN2C3CC1C(C45C3(NC6=CC=CC=C64)OC2C5)C(=O)OC
Pinitol	C[C@@H]1C([C@@H](C(C([C@@H]1O)O)O)O)O
Pinocembrine	c1ccc(cc1)C1CC(=O)c2c(cc(cc2O1)O)O
Pinoresinol	COC1=C(C=CC(=C1)C2C3COC(C3CO2)C4=CC(=C(C=C4)O)OC)O
Pinostrobin	COC1=CC(=C2C(=O)CC(OC2=C1)C3=CC=CC=C3)O
Platyphylline	C/C=C\1/C[C@@H](C)C[C@](C)(C(=O)OCC2CCN3CC[C@H]([C@@H]23)OC1=O)O
Plumbagin	CC1=CC(=O)c2c(cccc2O)C1=O
Polygodial	CC1(C)CCC[C@]2(C)[C@@H](C=O)C(=CC[C@@H]12)C=O
Potassium nitrate	[K]N(=O)([O-])[O-]
Potassium nitrite	[K]N(=O)=O
Powelline	COC1=C2CN3CCC4(C3CC(C=C4)O)C2=CC5=C1OCO5
Pratorimine	COc1cc2c(cc1O)c1cccc3CCn(c13)c2=O
Pratorinine	Oc3cc4c2cccc1ccn(c12)C(=O)c4cc3OC
Premarrubin	C[C@@H]1C[C@@H]2[C@H]3[C@](C)(CCC[C@]3(C)[C@]31CCC1(C=COC1)O3)C(=O)O2
Presenegenin	CC1(C)CC[C@@]2(CCC3(CO)C(=CCC4[C@@]5(C)C[C@@H]([C@@H]([C@](C)(C5CC[C@@]34C)C(=O)O)O)O)[C@@H]2C1)C(=O)O
Primin	CCCCCC1=CC(=O)C=C(C1=O)OC
Proacacipetalin	C=C(C)[C@@H](C#N)OC1C(C(C(C(CO)O1)O)O)O
Procumbide	C[C@@]12[C@H]3[C@@H](OC=C[C@]3([C@H]([C@@H]1O2)O)O)OCC(C(C(C(C=O)O)O)O)O
Procyanidin C1	c1cc(c(cc1[C@@H]1C(Cc2c(cc(c(C3c4c(cc(c(C5c6c(cc(cc6O[C@H](c6ccc(c(c6)O)O)C5O)O)O)c4O[C@H](c4ccc(c(c4)O)O)C3O)O)O)c2O1)O)O)O)O)O
Propacin	Oc1ccc(cc1OC)[C@H]2Oc3c(O[C@@H]2C)c4OC(=O)C=Cc4cc3OC
Proscillardin A	CC1C(C(C(C(O1)O[C@H]1CC[C@@]2(C)C(=C1)CCC1C2CC[C@]2(C)[C@H](CC[C@]12O)c1ccc(=O)oc1)O)O)O
Protoanemonin	C=C1C=CC(=O)O1
Protocatechuic acid	c1cc(c(cc1C(=O)O)O)O
Protopine	CN1CCC2=CC3=C(C=C2C(=O)CC4=C(C1)C5=C(C=C4)OCO5)OCO3

Prunasin	c1ccc(cc1)[C@H](C#N)OC1C(C(C(C(CO)O1)O)O)O
Pseudobaptigen	Oc3cc4OC=C(c1ccc2OCOc2c1)C(=O)c4cc3
Psoralen	c1cc(=O)oc2cc3c(cco3)cc12
Ptaquiloside	C[C@@H]1C[C@]2(C=C(C)C3(CC3)[C@](C)([C@@H]2C1=O)O)OC1[C@H]([C@H](C(C(CO)O1)O)O)O
Ptearoxylone	Cc1cc(=O)c2c(cc3c(C=CC(=O)CO3)c2O)o1
pterygospermin	C1=CC=C(C=C1)CN2C(=S)OC23C=CC4(C=C3)N(C(=S)O4)CC5=CC=CC=C5
Pulegone	CC(=C1CC[C@@H](C)CC1=O)C
Punicalagin	C1C2C(C3C(C(O2)O)OC(=O)C4=CC(=C(C(=C4C5=C(C(=C(C=C5C(=O)O3)O)O)O)O)O)O)OC(=O)C6=CC(=C(C(=C6C7=C(C(=C8C9=C7C(=O)OC2=C(C(=C(C3=C(C(=C(C=C3C(=O)O1)O)O)O)C(=C92)C(=O)O8)O)O)O)O)O)O)O
Punicalin	C1C2C(C(C(C(O2)O)O)O)OC(=O)C3=CC(=C(C(=C3C4=C(C(=C5C6=C4C(=O)OC7=C(C(=C(C8=C(C(=C(C=C8C(=O)O1)O)O)O)C(=C67)C(=O)O5)O)O)O)O)O)O)O
Punicic acid	CCCC/C=C\C=C\C=C/CCCCCCCC(=O)O
Pyrethrin I	C=C/C=C\CC1=C(C)[C@H](CC1=O)OC(=O)[C@@H]1[C@@H](C=C(C)C)C1(C)C
Pyrethrin II	C=C/C=C\CC1=C(C)[C@H](CC1=O)OC(=O)[C@@H]1[C@@H](/C=C(\C)/C(=O)OC)C1(C)C
Pyridoxine	Cc1c(c(CO)c(cn1)CO)O
Pyrogallol	c1cc(c(c(c1)O)O)O
Quercetin	c1cc(c(cc1c1c(c(=O)c2c(cc(cc2o1)O)O)O)O)O
Quercetin-3,7-diglucoside	C1=CC(=C(C=C1C2=C(C(=O)C3=C(C=C(C=C3O2)OC4C(C(C(C(O4)CO)O)O)O)O)OC5C(C(C(C(O5)CO)O)O)O)O)O
Quercetin-3-glucoside	C1=CC(=C(C=C1C2=C(C(=O)C3=C(C=C(C=C3O2)O)O)OC4C(C(C(C(O4)CO)O)O)O)O)[O-]
Quercetin-3-O-(6'-malonyl-glucoside)	C1=CC(=C(C=C1C2=C(C(=O)C3=C(C=C(C=C3O2)O)O)OC4C(C(C(C(O4)COC(=O)CC(=O)O)O)O)O)O)O
Quercetin-7 O-β-D-xylopyranoside	c1cc(c(cc1c1c(c(=O)c2c(cc(cc2o1)OCC(C(C(C(C=O)O)O)O)O)O)O)O)O
Quercitrin	CC1C(C(C(C(O1)OC2=C(OC3=CC(=CC(=C3C2=O)O)O)C4=CC(=C(C=C4)O)O)O)O)O
Quindoline	C1=CC=C2C(=C1)C=C3C(=N2)C4=CC=CC=C4N3
Ranunculin	C1=CC(=O)O[C@H]1COC1C(C(C(C(CO)O1)O)O)O
Rauvoxin	O=C(OC)C3=CO[C@@H](C)[C@@H]4[C@H]6N5CCC1(c2cc(OC)c(OC)cc2NC1=O)C56C[C@H]34
Rauvoxinin	O=C(OC)C3=CC(C)[C@@H](C)[C@@H]4CN5CCC1(c2cc(OC)c(OC)cc2NC1=O)C5C[C@H]34
Reserpic acid	COC1C(CC2CN3CCC4=C(C3CC2C1C(=O)O)NC5=C4C=CC(=C5)OC)O.Cl
Reserpiline	CC1C2CN3CCC4=C(C3CC2C(=CO1)C(=O)OC)NC5=CC(=C(C=C45)OC)OC
Reserpine	COc1cc(cc(c1OC)OC)C(=O)O[C@@H]1C[C@@H]2CN3CCc4c5ccccc5[nH]c4[C@H]3C[C@@H]2[C@@H]([C@H]1OC)C(=O)OC
Reservatrol	C(=C\c1cc(cc(c1)O)O)/c1ccc(cc1)O
Resveratrol-3-O-β-rutinoside	Oc1ccc(cc1)/C=C/c2cc(cc(O)c2)O[C@@H]4O[C@H](COC3O[C@@H](C)[C@H](O)[C@@H](O)[C@H]3O)[C@@H](O)[C@H](O)[C@H]4O

Retrorsine	C/C=C\1/C[C@@H](C)[C@](CO)(C(=O)OCC2=CCN3CC[C@H]([C@@H]23)OC1=O)O
Rhinocerotinoic acid	C/C(=C\C(=O)O)/CCC1=C(C)C(=O)C[C@H]2C(C)(C)CCC[C@]12C
Ricinine	Cn1ccc(c(C#N)c1=O)OC
Ricinoleic acid	CCCCCCC(C/C=C\CCCCCCCC(=O)O)O
Rohituka 3	CCC(C)C(C(=O)OC1C2C(C(=C)C3(C1(C(CC3=O)C4=COC=C4)C)O)C5(C6CC(=O)OCC6(OC(=O)CC5O2)C)C)O
Rooperol	C(/C=C/c1ccc(c(c1)O)O)C#Cc1ccc(c(c1)O)O
Rosmarinic acid	c1cc(c(cc1/C=C/C(=O)O[C@@H](Cc1ccc(c(c1)O)O)C(=O)O)O)O
Rotenone	C=C(C)[C@H]1Cc2c(ccc3C(=O)[C@H]4c5cc(c(cc5OC[C@H]4Oc23)OC)OC)O1
Rubiadin	Cc1c(cc2c(c1O)C(=O)c1ccccc1C2=O)O
Rubiadin xyloglucoside	O=C4c3cc(OC2OC(CO)C(O)C(O)C2OC1OCC(O)C(O)C1O)c(C)c(O)c3C(=O)c5ccccc45
Ruscogenin-(25S)-form	C[C@@H]1CC[C@@]2([C@H](C)[C@H]3[C@H](CC4C5CC=C6C[C@H](C[C@H]([C@]6(C)C5CC[C@]34C)O)O)O2)OC1
Rutaretin	CC(C)(C1CC2=C(O1)C(=C3C(=C2)C=CC(=O)O3)O)O
Rutin	CC1C(C(C(C(O1)OCC2C(C(C(C(O2)OC3=C(OC4=CC(=CC(=C4C3=O)O)O)C5=CC(=C(C=C5)O)O)O)O)O)O)O)O
Sabinene	CC(C)[C@@]12CCC(=C)[C@H]2C1
Sakurasosaponin	O[C@H]%11[C@@H](O)[C@@H](O)C(C)OC%11O[C@H]%10[C@@H](O)[C@@H](O)C(C)OC%10O[C@H]9[C@@H](O)[C@@H](O)C(CO)OC9O[C@@H]2[C@H](OC1OC(CO)[C@@H](O)[C@H](O)[C@@H]1O)C(OC(C(=O)O)[C@H]2O)O[C@H]8CC[C@@]7(C)C(CC[C@]6(C)C7CC[C@]35OCC4(CC[C@@](C)(C)CC34)[C@H](O)C[C@]56C)[C@]8(C)C
Salicin	c1ccc(c(c1)CO)OCC(C(C(C(C=O)O)O)O)O
Salicortin	c1ccc(c(c1)COC(=O)C1(C=CCC1=O)O)OCC(C(C(C(C=O)O)O)O)O
Salicylic acid	c1ccc(c(c1)C(=O)O)O
Salutarisolide	CC1CC[C@@]2(C)C(CC=C3C(=O)OC[C@]23O)C1=C
Sanguinarine	C[n+]1cc2c(ccc3c2OCO3)c2ccc3cc4c(cc3c12)OCO4
Sanjoinine A	CC(C)CC1C(=O)N/C=C\c2ccc(cc2)OC(C(C)C)C(C(=O)N1)NC(=O)C(Cc1ccccc1)N(C)C
Santin	COc1ccc(cc1)c1c(c(=O)c2cc(c(cc2o1)CO)OC)OC
Saponin C	CC1CC[C@@]2([C@@H](C)C3C(CC4C5CC=C6C[C@H](C[C@H]([C@]6(C)C5CC[C@]34C)OC3C(C(C(C(OC(=O)C)C)O3)O)O)OC3C(C(C(C(C)O3)O)O)O)O)O2)OC1
sarcovimiside B	C[C@H]1[C@@H]([C@@H](C[C@@H](O1)O[C@@H]1[C@@H](C)O[C@H](C[C@@H]1OC)O[C@@H]1[C@@H](C)O[C@H](C[C@@H]1O)O[C@H]1CC[C@]2(C)[C@@H](C[C@H]([C@@]3(C)C(=O)CC[C@]3([C@H](C)OC(=O)c3ccccc3)O)OC(=O)c3ccccc3)C(=O)C=C[C@@]2(C1)O)OC)O
Sauveoline	CN1CCC2=CC(=C(C3=C2C1CC4=C3C(=C(C=C4)O)O)OC)OC
Sceleratine	CC1C(C(C(=O)OC2CCN3C2C(=CC3)COC(=O)C1(C)O)(CO)O)C
Scillaren A	CC1[C@@H](C([C@@H]([C@@H](O1)OC1CC[C@@]2(C)C(=C1)CCC1C2CC[C@]2(C)[C@H](CC[C@]12O)c1ccc(=O)oc1)O)O)O[C@H]1C([C@H]([C@H](C(CO)O1)O)O)O
Scillarenin	C[C@]12CC[C@@H](C=C1CCC1C2CC[C@]2(C)[C@H](CC[C@]12O)c1ccc(=O)oc1)O

Scopolamine	CN1C2CC(CC1C1C2O1)OC(=O)[C@H](CO)c1ccccc1
Scopoletin	COc1cc2ccc(=O)oc2cc1O
secologanin	COC(=O)C1=CO[C@H]([C@@H]([C@@H]1CC=O)C=C)O[C@H]2[C@@H]([C@H]([C@@H]([C@H](O2)CO)O)O)O
Securinine	C1CCN2[C@H]3C=CCC4=CC(=O)O[C@]4(C3)[C@H]2C1
Senecionine	C/C=C\1/C[C@@H](C)C[C@](C)(C(=O)OCC2=CCN3CC[C@H]([C@@H]23)OC1=O)O
Sennoside A	c1cc2C(c3cc(ccc3C(=O)c2c(c1)OCC(C(C(C(C=O)O)O)O)O)C(=O)O)C1c2cccc(c2C(=O)c2c1cc(cc2O)C(=O)O)OCC(C(C(C(C=O)O)O)O)O
Sericic acid	CC1(C)CC[C@@]2(CC[C@]3(C)C(=CCC4[C@@]5(C)C[C@H]([C@@H]([C@](C)(CO)C5CC[C@@]34C)O)O)[C@@H]2C1)C(=O)O
Sericoside	CC1(CCC2(CCC3(C(=CCC4C3(CCC5C4(CC(C(C5(C)CO)O)O)C)C)C2C1O)C)C(=O)OC6C(C(C(C(O6)CO)O)O)O)C
Serpenticine	O=C(OC)C3=CO[C@H](C)[C@H]4C[n+]5ccc1c(nc2cc(OC)ccc12)c5C[C@H]34
serpentine	CC1C2CN3C=CC4=C5C=CC=CC5=NC4=C3CC2C(=CO1)C(=O)OC
Sesbanimide A	C=C1CO[C@]([C@@H]1C)([C@@H]1[C@@H]([C@H](C2CC(=O)NC(=O)C2)OCO1)O)O
Sesbanimide B	C=C1COC(C1C)(C1C(C(C2CC(=NC(=O)C2)O)OCO1)O)O
Sesbanimide C	C=C1COC(C1C)(C(C(CC1CC(=NC(=O)C1)O)O)OC)O
Siphonochilone	CC1C=CC(=O)C2(C)Cc3c(CC12)c(C)co3
Sitosterol-3-glucoside	CC[C@H](CC[C@@H](C)[C@H]1CC[C@H]2[C@@H]3CC=C4C[C@H](CC[C@]4(C)[C@H]3CC[C@]12C)O[C@H]1[C@@H]([C@H]([C@@H]([C@@H](CO)O1)O)O)O)C(C)C
Sivasinolide	CC1=CC[C@H]([C@]2(C)C[C@H]3[C@@H](C(=C)C(=O)O3)[C@@H](C12)O)O
Skimmianine	COc1ccc2c(c1OC)nc1c(cco1)c2OC
Sodium ent knipholone 6-O-sulfate	CC(=O)c1c(cc(c(c2c(cc(c3c2C(=O)c2cccc(c2C3=O)O)O)CO)c1OS(O)(O)O[Na])O)C=O
Solanine	C[C@H]1CC[C@@H]2C(C)C3C(CC4C5CC=C6C[C@H](CC[C@]6(C)C5CC[C@]34C)OC3C(C(C(C(CO)O3)O)OC3C(C(C(C(CO)O3)O)O)O)OC3C(C(C(C(C)O3)O)O)O)N2C1
Solanocapsine	C[C@@H]1C[C@]2([C@@H]([C@@H](C)C3C(CC4C5CC[C@H]6C[C@H](CC[C@]6(C)C5CC[C@]34C)N)O2)NC1)O
Solasodine	C[C@@H]1CC[C@@]2([C@@H](C)[C@H]3[C@H](CC4C5CC=C6C[C@H](CC[C@]6(C)C5CC[C@]34C)O)O2)NC1
Solasonine	CC1CCC2(C(C3C(O2)CC4C3(CCC5C4CC=C6C5(CCC(C6)OC7C(C(C(C(O7)CO)O)OC8C(C(C(C(O8)CO)O)O)O)OC9C(C(C(C(O9)C)O)O)O)C)C)C)NC1
Sophoracoumestan B	COC1=C(C=CC2=C1OC(=O)C3=C2OC4=CC5=C(C=C43)OCO5)O
Sophoricoside	C1=CC(=CC=C1C2=COC3=CC(=CC(=C3C2=O)O)O)OC4C(C(C(C(O4)CO)O)O)O
Sparteine	C=C1CCC[C@@H]2[C@H]3C[C@H](CN12)[C@@H]1CCCCN1C3
Spathulenol	C=C1CC[C@@H]2[C@H]([C@H]3[C@H]1CC[C@]3(C)O)C2(C)C
Stachenol	CC1(C)C2CC[C@]34C=C[C@@](C)(CC[C@H]4[C@]2(C)CC[C@@H]1O)C3
Stachenone	CC1(C)C2CC[C@]34C=C[C@@](C)(CC[C@H]4[C@]2(C)CCC1=O)C3
Stachydrine	C[N+]1(C)CCC[C@@H]1C(=O)O

Stearic acid	Cn1c(cnc(c1=O)NC(=N)N)CO
Sterol	C1CC2CCC3C4CCC(CC4CCC3C2C1)O
Stigmast-4-en-3-one	CCC(CCC(C)C1CCC2C1(CCC3C2CCC4=CC(=O)CCC34C)C)C(C)C
Stigmasterol	CCC(C=CC(C)C1CCC2C1(CCC3C2CC=C4C3(CCC(C4)O)C)C)C(C)C
Stizolamine	CN1C(=CN=C(C1=O)N=C(N)N)CO
Stoloniferin III	CCCCCCCCCC(=O)O[C@@H]1[C@@H]([C@H]([C@H](C)O[C@H]1O[C@H]1[C@H](C)O[C@@H]2[C@@H]([C@@H]1OC(=O)CCCCCCCCC[C@H](CCCCC)O[C@H]1[C@@H]([C@H]([C@H]([C@@H](C)O1)O)O)O2)O)O[C@H]1[C@@H]([C@@H]([C@H]([C@H](C)O1)OC(=O)[C@@H](C)CC)O)O)O[C@H]1[C@@H]([C@@H]([C@H]([C@H](C)O1)O)O)O
Stricosamine	C=CC1C2CC3C4=C(CCN3C(=O)C2=COC1OC5C(C(C(C(O5)CO)O)O)O)C6=CC=CC=C6N4
Strictosidine	COC(=O)C1=COC(C(C1CC2C3=C(CCN2)C4=CC=CC=C4N3)C=C)OC5C(C(C(C(O5)CO)O)O)O
Strogoside	CC1C(C(C(C(O1)OC2CC(C34C5C(CCC3(C2)O)C6(CCC(C6(CC5OC4=O)C)C7=CC(=O)OC7)O)O)O)O)O
Strophanthidin	C[C@@]12CCC3C(CC[C@@]4(C[C@H](CC[C@]34CO)O)O)[C@]2(CC[C@@H]1C1=CC(=O)OC1)O
Strychnine	c1ccc2c(c1)[C@]13CCN4CC5=CCO[C@H]6CC(=O)N2[C@H]3[C@H]6[C@H]5C[C@@H]14
Succinic acid	C(CC(=O)O)C(=O)O
Sucrose	C(C1[C@H](C([C@H]([C@H](O1)O[C@]1(CO)C([C@H]([C@@H](CO)O1)O)O)O)O)O)O
Sutherlandioside B	C[C@H](CCC(C(C)(C)OCC(C(C(C(C=O)O)O)O)O)O)[C@H]1CC[C@@]2(C)C3C(CC4C(C)(C)[C@@H](CC(=O)[C@]54C[C@@]35CC[C@]12C)O)O
Sweroside	C=CC1C2CCOC(=O)C2=COC1OC3C(C(C(C(O3)CO)O)O)O
Swertiamarine	C=C[C@H]1[C@@H](OC=C2C(=O)OCC[C@@]12O)O[C@H]1[C@@H]([C@H]([C@@H]([C@@H](CO)O1)O)O)O
Symphytine	C/C=C(\C)/C(=O)O[C@@H]1CCN2CC=C([C@H]12)OC(=O)[C@@](C(C)C)([C@H](C)O)O
Synaptoleptis factor K1	CCCCCCCCCCCCC/C=C/C12O[C@@H]3C4[C@H]5[C@@](CO)([C@H]([C@]6([C@@H](C=C(C)C6=O)[C@]4([C@H](C)C[C@]3(C(=C)C)O1)O2)O)O)O5
Synaptoleptis factor K7	C=C(C)[C@]12C[C@@H](C)[C@@]34[C@@H]5[C@H]1OC(/C=C/CCCCCCCCCCCCC1[C@H](C)C(=O)[C@@]([C@@H]([C@]6(CO)[C@H]5O6)O)([C@]31C)O)(O2)O4
Syringic acid	COC1=CC(=CC(=C1O)OC)C(=O)O
Tabersonine	CC[C@]12C=CCN3CC[C@@]4(c5ccccc5NC4=C(C1)C(=O)OC)[C@H]23
Taraxerol	CC1(CCC2(CC=C3C4(CCC5C(C(CCC5(C4CCC3(C2C1)C)C)O)(C)C)C)C)C
Tartaric acid	C(C(C(=O)O)O)(C(=O)O)O
Tatridin A	C/C/1=C/[C@H]([C@@H]2C(=C)C(=O)O[C@H]2/C=C(/C)\[C@H](CC1)O)O
Taxine A	CC1=C2[C@H](C(=O)[C@@]3(C)C/C(=C\[C@@H](C(C[C@@H]1OC(=O)C)C2(C)C)OC(=O)C)/[C@H](C([C@@H]3O)OC(=O)[C@@H]([C@H](C1CCCCC1)N(C)C)O)O)O
Tephrosin	CC1(C)C=Cc2c(ccc3c2O[C@@H]2COc4cc(c(cc4[C@@]2(C3=O)O)OC)OC)O1
Terminoic acid	O=C(O)[C@]4(C)CC[C@]5(C)CC[C@]3(C)C(=CCC2[C@@]3(C)CCC1[C@]2(C)C[C@@H](O)[C@H](O)C1(C)C)C5[C@@H]4O

Terpinen-4-ol	CC(C)C1(CC=C(C)CC1)O
Terpineol	CC1=CCC(CC1)C(C)(C)O
Terpinolene	CC(=C1CC=C(C)CC1)C
Tetracoson-1-ol	CCCCCCCCCCCCCCCCCCCCCCCCO
Tetrahydroalstonine	CC1C2CN3CCC4=C(C3CC2C(=CO1)C(=O)OC)NC5=CC=CC=C45
Tetrahydrocannabinol	CCCCCc1cc(c2C3C=C(C)CC[C@H]3C(C)(C)Oc2c1)O
Tetrahydroxyflavanone	c1cc2C(=O)C[C@@H](c3cc(c(cc3O)O)O)Oc2cc1O
Tetraphylline	C1=C[C@@](C[C@@H]1O)(C#N)OC1C(C(C(C(CO)O1)O)O)O
Teutrifidin	C[C@@H]1[C@H](C(=O)[C@]2(COC(=O)C)[C@H](CCC(=O)[C@@]32CO3)[C@]21C[C@@H](c1ccoc1)OC2=O)O
Thesinine	C1C[C@@H]2[C@@H](CCN2C1)COC(=O)/C=C/c1ccc(cc1)O
Thesiuside	CC(=O)O[C@@]12CCC3C(CC[C@]4(C)[C@H](CC[C@]34O)c3ccc(=O)oc3)[C@]2(CC[C@@H](C1)OC1C(C(C(C(CO)O1)O)O)O)CO
Thevetin A	C[C@H]1[C@@H]([C@H]([C@@H]([C@@H](O1)O[C@H]1CC[C@@]2(C=O)[C@H](CC[C@@H]3[C@@H]2CC[C@]2(C)[C@H](CC[C@]32O)C2=CC(=O)OC2)C1)O)OC)O[C@H]1[C@@H]([C@H]([C@@H]([C@@H](CO[C@H]2[C@@H]([C@H]([C@@H]([C@@H](CO)O2)O)O)O)O1)O)O)O
Thevetin B	CC1C(C(C(C(O1)O[C@H]1CC[C@@]2(C)[C@H](CCC3C2CC[C@]2(C)[C@H](CC[C@]32O)C2=CC(=O)OC2)C1)O)OC)OC1C(C(C(C(COC2C(C(C(C(CO)O2)O)O)O)O1)O)O)O
Thiamine	CC1=C(SC=[N+]1CC2=CN=C(N=C2N)C)CCO
Thrichilin A	CCC(C)C(=O)OC1C2(C)C3C[C@H]([C@]4(C)C(C(=O)[C@H]([C@@]5(C)[C@@H](C[C@@H]6[C@]45O6)c4ccoc4)O)[C@@]3(CO1)[C@H]([C@H]([C@H]2OC(=O)C)OC(=O)C)O)O
Toddaculin	CC(=CCc1c(cc2c(ccc(=O)o2)c1OC)OC)C
Toddalenol	CC(C)(O)/C=C/c1c(cc2OC(=O)C=Cc2c1OC)OC
Toddalenone	CC(=O)/C=C/c1c(cc(c2ccc(=O)oc12)OC)OC
Toddalolactone	CC(C)(C(Cc1c(cc2c(ccc(=O)o2)c1OC)OC)O)O
Toddalosin	CC1=C[C@H](c2c(cc(c3ccc(=O)oc23)OC)OC)[C@@H]([C@H](c2c(cc(c3ccc(=O)oc23)OC)OC)O)C(C)(C)C1
Toddasin	CC1(CCC=CC1c1c(cc(c2ccc(=O)oc12)OC)OC)/C=C/c1c(cc(c2ccc(=O)oc12)OC)OC
Tortuosamine	CNCC[C@@]1(CCc2c(cccn2)C1)c1ccc(c(c1)OC)OC
Trachelogenin	COC1=C(C=C(C=C1)CC2COC(=O)C2(CC3=CC(=C(C=C3)O)OC)O)OC
Trans-Pinocarveol	C=C1C2CC(CC1O)C2(C)C
Trans-p-Mentha-1-7-8-dien-2-ol	C=C(C)[C@@H]1CCC(=C)[C@H](C1)O
TR-B	CCC(C)C(O)C(=O)OC2C(OC=O)C(C(=C)C3(O)C(=O)CC(c1ccoc1)C23C)C4(C)C(CC(=O)OC5(C)COC(=O)CC45)OC(C)=O
Trehalose	C([C@@H]1[C@H]([C@@H]([C@H]([C@H](O1)O[C@@H]1[C@@H]([C@H]([C@@H]([C@@H](CO)O1)O)O)O)O)O)O)O
Triacontane	CCCCCCCCCCCCCCCCCCCCCCCCCCCCCC

Trimethylamine	CN(C)C
Tutin	C=C(C)[C@H]1[C@@H]2C(=O)O[C@H]1[C@H]([C@@]1(C)[C@@]3(CO3)[C@H]3[C@@H]([C@@]21O)O3)O
Tyledoside C	C[C@@H]1CC([C@]2([C@@H](O1)O[C@@H]1CC3C[C@H]4[C@@]5(C([C@@H](C(=O)[C@]6(C)[C@H](CC[C@]56O)c5ccc(=O)oc5O)[C@@]3(C)C[C@H]1O2)O4)O)OC(=O)C
Typhaphtalide	c1ccc(cc1)CC1c2cccc(c2C(=O)O1)O
Typharin	c1ccc(cc1)/C=C/C1Cc2cccc(c2C(=O)O1)O
Typhasterol	CC[C@H](C)[C@H]([C@@H]([C@@H](C)[C@H]1CCC2[C@]1(C)CCC1[C@@]3(C)CC[C@H](C[C@@H]3C(=O)C[C@@]21C)O)O)O
Tyramine	c1cc(ccc1CCN)O
Ulopterol	CC(C)([C@@H](Cc1cc2ccc(=O)oc2cc1OC)O)O
Umckalin	COc1c2ccc(=O)oc2cc(c1OC)O
Umtatin	C=C(C)C1Cc2c(cc3c(c(=O)cc(CO)o3)c2O)O1
Umuravumbolide	CCCCC(/C=C\C1CC=CC(=O)O1)OC(=O)C
Undulatine	COC1CC2C3(CCN2CC4=C(C5=C(C=C43)OCO5)OC)C6C1O6
Uric acid	c12c([nH]c(=O)[nH]2)[nH]c(=O)[nH]c1=O
Ursolic acid	C[C@@H]1CC[C@@]2(CC[C@]3(C)C(=CCC4[C@@]5(C)CC[C@@H](C(C)(C)C5CC[C@@]34C)O)C2[C@H]1C)C(=O)O
Urushiol component	CCCCCC/C=C\CCCCCCCc1cccc(c1O)O
Urushiol III	CCC/C=C/C/C=C/CCCCCCCc1cccc(c1O)O
Uzarigenin	C[C@]12CC[C@@H](C[C@@H]1CC[C@@H]1[C@@H]2CC[C@]2(C)[C@H](CC[C@]12O)C1=CC(=O)OC1)O
Uzarin	C[C@]12CCC(C[C@@H]1CCC1C2CC[C@]2(C)[C@H](CC[C@]12O)C1=CC(=O)OC1)OCC(C(C(C(COOCC(C(C(C(C=O)O)O)O)O)O)O)O)O
Valeranone	CC(C)[C@@H]1CC[C@]2(C)CCCC(=O)[C@]2(C)C1
Valerenic acid	C[C@@H]1CC[C@@H](/C=C(\C)/C(=O)O)C2=C(C)CC[C@H]12
Valtrate	CC(C)CC(=O)O[C@H]1C=C2C(=CO[C@H]([C@@H]2[C@]21CO2)OC(=O)CC(C)C)COC(=O)C
Vanillic acid	COC1=C(C=CC(=C1)C(=O)O)O
Vellosimine	CC=C1CN2C3CC1C(C2CC4=C3NC5=CC=CC=C45)C=O
Venusol	c1cc(ccc1/C=C\1/C(=O)OC2C(C(C(CO)OC2O1)O)O)O
vermeeric acid	C(=C(\CC(=O)O)/C(=O)O)\C(=O)O
Vermeerin	C[C@@H]1C[C@H]2[C@H](C[C@]3(C)COC(=O)C[C@@H]13)C(=C)C(=O)O2
verminoside	C1=COC(C2C1C(C3C2(O3)CO)OC(=O)C=CC4=CC(=C(C=C4)O)O)OC5C(C(C(C(O5)CO)O)O)O
Vernodalin	C=CC12CC(C3C(C1C(=C)C(=O)OC2)OC(=O)C3=C)OC(=O)C(=C)CO
Vernodalol	COC(=O)C(=C)C1C(CC2(COC(=O)C(=C)C2C1O)C=C)OC(=O)C(=C)CO
Vernolepin	C=CC12CC(C3C(C1C(=C)C(=O)OC2)OC(=O)C3=C)O

Vernolide	CC(=C)C(=O)OC1CC23C(O2)CCC(=CC4C1C(=C)C(=O)O4)COC3O
Vernomydin	CC(C)C(=O)O[C@H]2C[C@]14O[C@@H]4CCC(COC1O)=C[C@H]3OC(=O)C(=C)[C@H]23
Vernonioside A1	CC(C)[C@]1(O[C@H]1C)[C@H]2OC(=O)C([C@@H2]2)C3C(O)C[C@H]4C5=CC[C@H]6CC(CC[C@]6(C)C5=CC[C@]34C)O[C@@H]7O[C@H](CO)[C@@H](O)[C@H](O)[C@H]7O
Vernonioside A3	CC(C)[C@]1(O[C@H]1C)[C@H]2OC(=O)C([C@@H2]2)C3C(=O)C[C@H]4C5=CC[C@H]6CC(CC[C@]6(C)C5=CC[C@]34C)O[C@@H]7O[C@H](CO)[C@@H](O)[C@H](O)[C@H]7O
Vernonioside B1	CC(C)[C@]1(O[C@H]1C)[C@H]2OC(=O)C([C@@H]2O)C3CC[C@H]4C5=CC[C@H]6CC(CC[C@]6(C)C5=CC[C@]34C)O[C@@H]7O[C@H](CO)[C@@H](O)[C@H](O)[C@H]7O
Vernonioside B2	OC7C(O)C(O)C(CO)O[C@H]7OC1CC[C@]2(C)C3=CC[C@]4(C)C([C@H](O)C[C@H]4C3=CC[C@H]2C1)[C@@H]6[C@H](O)O[C@H]5[C@@H]6O[C@](C)(OC)[C@]5(O)C(C)C
Vernonioside B3	CC(C)[C@]1(O[C@H]1C)[C@H]2OC(=O)[C@H]([C@@H2]2)[C@H]3C(OC(C)=O)C[C@H]4C5=CC[C@H]6CC(CC[C@]6(C)C5=CC[C@]34C)O[C@@H]7O[C@H](CO)[C@@H](O)[C@H](O)[C@H]7O
Vinblastine	CC[C@@]1(C[C@@H]2C[C@](c3cc4c(cc3OC)N(C)[C@@H]3[C@@]54CCN4CC=C[C@](CC)([C@@H]54)[C@H]([C@]3(C(=O)OC)O)OC(=O)C)(c3c(CCN(C2)C1)c1ccccc1[nH]3)C(=O)OC)O
Vinburnine	CCC12CCCN3C1C4=C(CC3)C5=CC=CC=C5N4C(=O)C2
Vincamine	CC[C@]12CCCN3CCc4c5ccccc5n(c4[C@H]23)[C@](C1)(C(=O)OC)O
Vincristine	CC[C@@]1(C[C@@H]2C[C@](c3cc4c(cc3OC)N(CO)[C@@H]3[C@@]54CCN4CC=C[C@](CC)(C54)[C@H]([C@]3(C(=O)OC)O)OC(=O)C)(c3c(CCN(C2)C1)c1ccccc1[nH]3)C(=O)OC)O
Vindoline	CCC12C=CCN3C1C4(CC3)C(C(C2OC(=O)C)(C(=O)OC)O)N(C5=C4C=CC(=C5)OC)C
Viscolin	COc1cc(c(c(c1CCCc1ccc(c(c1)OC)O)OC)OC)O
Viscumside A	COc1cc(ccc1O)[C@@H]1CC(=O)c2c(cc(cc2O1)OCC(C(C(C(C=O)O)O)O)O)O
Vitexin	C1=CC(=CC=C1C2=CC(=O)C3=C(O2)C(=C(C=C3O)O)C4C(C(C(C(O4)CO)O)O)O)O
Voacamidine	CCC1CC2CC3(C1N(C2)CCC4=C3NC5=C4C(=C(C=C5)OC)C6CC7C(C(CC8=C6NC9=CC=CC=C89)N(CC7=CC)C)C(=O)OC)C(=O)OC
Voacamine	CCC1CC2CC3(C1N(C2)CCC4=C3NC5=CC(=C(C=C45)OC)C6CC7C(C(CC8=C6NC9=CC=CC=C89)N(CC7=CC)C)C(=O)OC)C(=O)OC
Voacangine	CCC1CC2CC3(C1N(C2)CCC4=C3NC5=C4C=C(C=C5)OC)C(=O)OC
Voacorine	CC=C1CN(C2CC3=C(C(CC1C2C(=O)OC)C4=C(C=C5C6=C(C7(CC8CC(C7N(C8)CC6)C(C)O)C(=O)OC)NC5=C4)OC)NC9=CC=CC=C39)C
Voacristine	C[C@@H]([C@H]1C[C@@H]2C[C@]3(c4c(CCN(C2)[C@@H]13)c1cc(ccc1[nH]4)OC)C(=O)OC)O
Voaphylline	CC[C@]12CCc3c(CCN(C[C@@H]4[C@H]2O4)C1)c1ccccc1[nH]3
Vobtusine	COC1=CC=CC2=C1N3CC4(CC5C3(C26CCN7C6C8(C5)CCOC8CC7)O)CN9CCC12C9C3(C4OCC3)CC(=C1NC1=CC=CC=C21)C(=O)OC
Vomifoliol	OC1(CCC(C)O)C(C)=CC(=O)CC1(C)C
Warburganal	CC1(C)CCC[C@@]2(C)C1CC=C(CO)[C@@]2(CO)O

Warfarin	CC(=O)CC(C1CCCCC1)c1c(c2ccccc2oc1=O)O
Wighteone	CC(=CCc1c(cc2c(c1O)c(=O)c(co2)c1ccc(cc1)O)O)C
Withaferin A	CC1=C(CO)C(=O)OC[C@H]1[C@@H](C)C1CCC2C3C[C@@H]4[C@@]5([C@H](C=CC(=O)[C@]5(C)C3CC[C@]12C)O)O4
Withasomnine	c1ccc(cc1)c1cc2CCCn2n1
Xanthine	c1[nH]c2c(n1)[nH]c(=O)[nH]c2=O
Xanthinin	CC1CC2C(CC=C1C(CC(=O)C)OC(=O)C)C(=C)C(=O)O2
Xanthotoxin	COc1c2c(cco2)cc2ccc(=O)oc12
Xylopic acid	CC(=O)OC1C(=C)C2CCC3C1(C2)CCC4C3(CCCC4(C)C(=O)O)C
Xysmalorin	C[C@]12CCC(CC1=CCC1C2CC[C@]2(C)[C@H](CC[C@]12O)C1=CC(=O)OC1)OCC(C(C(C(COOCC(C(C(C(C=O)O)O)O)O)O)O)O)O
Yamogenin	CC1CCC2(C(C3C(O2)CC4C3(CCC5C4CC=C6C5(CCC(C6)O)C)C)C)OC1
Yohimbine	COC(=O)C1C(CCC2C1CC3C4=C(CCN3C2)C5=CC=CC=C5N4)O
Yomogi alcohol	CC(C)(C=C)C=CC(C)(C)O
α-Amyrin	CC1CCC2(CCC3(C(=CCC4C3(CCC5C4(CCC(C5(C)C)O)C)C)C2C1C)C)C
α-Amyrin acetate	CC1CCC2(C)CCC3(C)C(=CCC4C5(C)CCC(C(C)(C)C5CCC34C)OC(=O)C)C2C1C
α-cadinol	CC1=CC2C(CCC(C2CC1)(C)O)C(C)C
α-caryophyllene	C/C/1=C\CC/C(=C/CC(C)(C)/C=C/C1)/C
α-Fenchol	CC1(C)[C@@H]2CC[C@@](C)(C2)C1O
α-Humulene	C/C/1=C/CC/C(=C\CC(C)(C)/C=C\C1)/C
α-Linolenic acid	CC/C=C\C/C=C\C/C=C\CCCCCCCC(=O)O
α-phellandrene	CC(C)C1C=CC(=CC1)C
α-pinene	CC1=CCC2CC1C2(C)C
α-spinasterol	CCC(C=CC(C)C1CCC2C1(CCC3C2=CCC4C3(CCC(C4)O)C)C)C(C)C
α-Terpinene	CC1=CCC(=CC1)C(C)C
α-Terpineol	CC1C=CC(CC1)C(C)(C)O
α-Thujone	CC(C)[C@]12CC2[C@@H](C)C(=O)C1
α-Zingiberene	CC(=CCC[C@H](C)[C@@H]1C=CC(=CC1)C)C
β-Amyrin	CC1(CCC2(CCC3(C(=CCC4C3(CCC5C4(CCC(C5(C)C)O)C)C)C2C1)C)C)C
β-Amyrin acetate	CC(=O)OC1CCC2(C)C(CCC3(C)C2CC=C2C4CC(C)(C)CCC4(C)CCC32C)C1(C)C
β-Asarone	CC=CC1=CC(=C(C=C1OC)OC)OC
β-Caryophyllene	C/C/1=C\CCC(=C)[C@H]2CC(C)(C)[C@@H]2CC1
β-Damascenone	C/C=C/C(=O)C1=C(C)C=CCC1(C)C

β-Glochidone	C=C(C)[C@@H]1CC[C@]2(C)CC[C@]3(C)[C@H](CC[C@@H]4[C@@]5(C)C=CC(=O)C(C)(C)[C@@H]5CC[C@@]34C)[C@@H]12
β-Methylglucoside	CO[C@H]1[C@@H](C([C@@H](C(CO)O1)O)O)O
β-Ocimene	C=C/C(=C\CC=C(C)C)/C
β-Phyllandrene	CC(C)C1C=CC(=C)CC1
β-Pinene	C=C1CCC2CC1C2(C)C
β-Sitosterol	CCC(CCC(C)C1CCC2C3CC=C4C[C@H](CC[C@]4(C)C3CC[C@]12C)O)C(C)C
β-Terpinene	CC(C)C1=CCC(=C)CC1
β-Thujone	CC(C)[C@]12CC2[C@H](C)C(=O)C1
β-Yohimbine	COC(=O)[C@H]1[C@@H](CC[C@@H]2[C@@H]1C[C@H]3c4c(c5ccccc5[nH]4)CCN3C2)O
γ-Diosphenol	CC(C)C1=C(C(=C)C(C)CC1)O
γ-Terpinene	CC1=CCC(=CC1)C(C)C
δ-7-Avenasterol	CC=C(CCC(C)C1CCC2C1(CCC3C2=CCC4C3(CCC(C4)O)C)C)C(C)C
δ-Camphor	CC1(C2CCC1(C(=O)C2)C)C
ε-phytol	CC(C)CCC[C@@H](C)CCC[C@@H](C)CCCC(\C)=C\CO

Table S2. Assigned numerical identity of the predicted active compounds, their plant sources and predicted targets.

Number	Compound	Plant	Predicted targets (docking score in kcal/mol)
1	1,2-Epoxyscillirosidin	*Homeria Pallida*	PPARD (-9.5)
2	1,5-Dicaffeoylquinic acid	*Vernonia amygdalina*	DPP4 (-9.2); GCK (-9.1); PPARD (-9.3); PPARG (-9.1)
3	10-Hydroxyakagerine	*Strychnos spinosa*	PPARD (-9.1)
4	12-B-hydroxyscillirosidin	*Drimia robusta*	DPP4 (-9.0); HSD11B1 (-9.3)
5	12-Hydroxydaphnetoxin	*Gnidia kraussiana*	PPARD (-10.8)
6	15-Oxo-kaurenoic acid	*Xylopia aethiopica*	HSD11B1 (-9.5); PPARD (-9.9); PPARG (-9.4)
7	16-B-Formyloxybovogenin A	*Moraea polystachya*	DPP4 (-9.1); HSD11B1 (-9.3); PPARD (-9.1); PPARG (-9.8)
8	1-Hydroxydimethylanthraquinone	*Danais fragrans*	AKR1B1 (-9.00); PPARD (-9.0); RBP4 (-10.1)
9	2-(1-Hydroxyethyl)naphtho[2,3-b]furan-4,9-dione	*Kigelia africana*	AKR1B1 (-9.6); FFAR1 (-9.2); RBP4 (-9.6)
10	2a,3b-Dihydroxyurs-12-en-18-oic acid	*Prunus africana*	AMY2A (-9.5); PYGL (-9.2)
11	Esculentic acid	*Prunus africana*	AMY2A (-9.0); HSD11B1 (-9.1)
12	3-Epicorosolic acid	*Prunus africana*	AMY2A (-9.6); PYGL (-9.3)
13	2-Hydroxygenistein	*Cajanus cajan*	AKR1B1 (-9.1)
14	3,3,4,5,5-Pentahydroxystilbene	*Schotia Brachypetala*	FFAR1 (-9.4)
15	3,8,11-Heptadecadienyl catechol	*Smodingium argutum*	AKR1B1 (-9.2); RBP4 (-9.0)
16	3-Acetylnerbowdine	*Boophane disticha*	RBP4 (-9.0)
17	3-Epioleanolic acid	*Melianthus Comosus, Prunus africana*	DPP4 (-10.4); HSD11B1 (-10.2)
18	3-Oxo-20,24-dammaradien-26-ol	*Rapanea melanophloeos*	AMY2A (-9.1); HSD11B1 (-9.9); PPARD (-9.9); PPARG (-9.1)
19	3,9-Dihydroeucomnalin	*Eucomnis autumnalis*	AKR1B1 (-9.1)
20	19-Deoxyuscharin	*Asclepias fruticosa*	DPP4 (-9.5); GCK (-9.7); PPARG (-10.4); PDK2 (-9.8)
21	4-Deoxy-13-O-phenylacetyl-12-O-tigloylphorbol	*Synadenium grantii*	PPARD (-10.2)
22	Umtatin	*Ptaeroxylon obliquum*	RBP4 (-9.1)
23	5-Chloropropacin	*Mondia whitei*	HSD11B1 (-9.9); PPARD (-10.0); PPARG (-9.2)
24	6-Methyldiosgenin	*Balanites aegyptiacus*	AMY2A (-9.1); PPARD (-9.5); PPARG (-10.4)
25	7-Hydroxy-4-6-dimethoxypropacin	*Mondia whitei*	HSD11B1 (-9.5); PPARG (-9.1)

26	8,(14),15-Isopimaradiene-7,18-diol	*Tetradenia Riparia*	HSD11B1 (-9.0); PPARD (-9.2)
27	8-Hydroxypinoresinol	*Olea europaea, Strophanthus gratus*	AKR1B1 (-9.1); GCK (-9.1); HSD11B1 (-9.0); PPARG (-9.0)
28	Alpha-Humulene	*Aframomum melegueta, Helichrysum Species*	RBP4 (-9.1)
29	Alpha-linolenic acid	*Adansonia digitata, Cajanus cajan*	AKR1B1 (-9.3); FFAR1 (-9.4); RBP4 (-10.0); RXRA (-9.4)
30	Acetylcaranine	*Amaryllis belladonna, Crinum bulbispermum*	AKR1B1 (-9.5); RBP4 (-10.7)
31	Acolongifloroside K	*Acokanthera oppositifolia, Strophanthus gratus*	AMY2A (-9.3)
32	Acovenoside A	*Acokanthera oppositifolia*	AMY2A (-9.6); DPP4 (-9.8); HSD11B1 (-9.5)
33	Adiantone	*Adiantum species*	GCK (-10.6); NR5A2 (-9.4); PPARD (-10.0); PPARG (-9.1)
34	Afrormosin	*Cyclopia spp*	AKR1B1 (-9.6)
35	Afroside	*Asclepias fruticosa*	DPP4 (-10.2); PPARD (-9.3); PTPN9 (-9.6); PYGL (-9.1)
36	Agapanthagenin	*Agapanthus Africanus*	AMY2A (-9.1); HSD11B1 (-9.3); PPARD (-9.0)
37	Ajmalicine	*Catharanthus roseus, Rauvolfia caffra*	GCK (-9.0); HSD11B1 (-9.6); PPARD (10.1)
38	Ajmaline	*Rauvolfia caffra, Rauvolfia vomitoria*	HSD11B1 (-9.4); PPARD (-9.8)
39	Akuammine	*Catharanthus roseus*	PPARD (-9.0)
40	Allouzarin	*Xysmalobium undulatum*	AMY2A (-9.2); DDP4 (-9.4); PDK2 (-9.3); PPARD (-9.2)
41	Alloxysmalorin	*Xysmalobium undulatum*	AMY2A (-9.5); DPP4 (-9.6); PYGL (-9.1)
42	Aloe-emodin anthrone	*Aloe ferox*	HSD11B1 (-9.1); RBP4 (-9.4)
43	Aloeresin A	*Aloe ferox*	AKR1B1 (-9.3); GCK (-9.8); HSD11B1 (-9.4); PPARD (-10.6); PPARG (-9.1)
44	Aloinoside A	*Aloe ferox*	AMY2A (-9.2); DPP4 (-9.4); HSD11B1 (-10.4)
45	Aloinoside B	*Aloe ferox*	AMY2A (-9.2); DPP4 (-9.4); HSD11B1 (-10.5)
46	Alpha-amyrin	*Artemisia afra, Euphorbia hirta, Impomoea pes-caprae ssp. Brasiliensis*	AMY2A (-9.5); HSD11B1 (-9.6); PTPn9 (-9.0)
47	Alpha-amyrin acetate	*Impomoea pes-caprae ssp. Brasiliensis*	HSD11B1 (-10.8)
48	Alpha-spinasterol	*Ravanela madagascariensis*	AKR1B1 (-9.0); HSD11B1 (-10.5); PPARD (-10.3)

49	Amentoflavone	*Xerophyta retinervis*	AMY2A (-10.4); DPP4 (-10.4); GCK (-10.0); HSD11B1 (-10.5); INSR (-10.0); MGAM (-9.8); NR5A2 (-9.0); PDK2 (-9.3); PPARA (-10.5); PPARD (-11.0); PPARG (-10.8); PYGL (-10.0)
50	Amritoside	*Psidium guajava*	AMY2A (-9.1); DPP4 (-10.4); HSD11B1 (-10.4); PPARD (-10.4); PPARG (-9.9)
51	Amygdalin	*Brabejum Stellatifolium, Prunus africana, Prunus Laurocerasus*	HSD11B1 (-9.3)
52	Angustine	*Nauclea latifolia*	AKR1B1 (-10.5); AMY2A (-9.1); DPP4 (-10.5); GCK (-9.4); HSD11B1 (-9.9); PPARD (-9.7); PPARG (-9.6)
53	Angustoline	*Nauclea latifolia*	AKR1B1 (-10.6); GCK (-10.5); HSD11B1 (-10.1); PPARD (-9.8); PPARG (-10.0)
54	Anolignan B	*Terminalia sericea*	AKR1B1 (-10.3); FFAR1 (-10.5); RBP4 (-9.1); RXR1 (-9.5)
55	Apigenin	*Cajanus cajan*	AKR1B1 (-9.1); HSD11B1 (-9.0); RBP4 (-9.9); RXRA (-9.1)
56	Apigenin dimethylether	*Rhus undulata*	HSD11B1 (-9.0); RBP4 (-10.0)
57	Arjunglucoside	*Terminalia sericea*	AMY2A (-9.3); DPP4 (-9.4)
58	Arjunolic acid	*Syzygium Cordatum*	AMY2A (-9.2)
59	Arnottianamide	*Toddalia asiatica*	HSD11B1 (-9.1)
60	Arvenin I	*Anagallis arvensis*	DPP4 (-9.0)
61	Arvenin II	*Anagallis arvensis*	DPP4 (-9.1)
62	Asiatic acid	*Centella asiatica*	AMY2A (-9.6); DPP4 (-9.4); HSD11B1 (-9.0)
63	Asiaticoside	*Centella asiatica*	AMY2A (-9.3); DPP4 (-9.6); PPARG (-9.9)
64	Aspalathin	*Aspalathus linearis*	AKR1B1 (-9.0); HSD11B1 (-9.2)
65	Autumnariniol	*Eucomnis autumnalis*	RBP4 (-9.0)
66	Autumnariol	*Eucomis autumnalis*	RBP4 (-9.0)
67	Avenasterol	*Adansonia digitata*	AKR1B1 (-10.3); HSD11B1 (-9.9); NR5A2 (-9.0); PPARD (-9.1); PPARG (-9.3); RXRA (-9.4)

68	Avicine	*Toddalia asiatica*	AKR1B1 (-10.6); AMY2A (-9.5); DPP4 (-9.9); GCK (-10.6); HSD11B1 (-10.4); INSR (-9.5); PPARD (-9.3); PPARG (-10.0); RXRA (-9.4)
69	Aviprin	*Ficus Salicifolia*	RBP4 (-9.4)
70	Beta-sitosterol	*Acacia karroo, Adansonia digitata, Centella asiatica, Dodonaea Angustifolia, Euphorbia hirta, Harpagophytum procumbens, Hypoxis hemerocallidea, Kigelia africana, Prunus africana, Ravenala madagascariensis, Syzygium Cordatum*	HSD11B1 (-9.1); PPARD (-10.1)
71	Balagyptin	*Balanites aegyptiacus*	AMY2A (-10.0); DPP4 (-9.9); HSD11B1 (-10.0); PPARG (-11.2)
72	Balanitin 1	*Balanites aegyptiacus*	AMY2A (-9.4); DPP4 (-10.5)
73	Balanitin 2	*Balanites aegyptiacus*	AMY2A (-9.3); DPP4 (-10.2); PPARG (-11.4)
74	Balanitin 3	*Balanites aegyptiacus*	AMY2A (-9.8); DPP4 (-10.0); GCK (-9.1); PPARD (-9.4); PPARG (-10.0); PDK2 (-9.4)
75	Balanitin 4	*Balanites aegyptiacus*	AMY2A (-9.9); DPP4 (-10.2); INSR (-9.8); PYGL (-9.2)
76	Balanitin 6	*Balanites aegyptiacus*	AMY2A (-9.0); DPP4 (-10.3)
77	Balanitin 7	*Balanites aegyptiacus*	DPP4 (-10.2)
78	Balanitoside	*Balanites aegyptiacus*	AMY2A (-9.0); DPP4 (-9.9); INSR (-9.1)
79	Bauerenol	*Adansonia digitata*	AMY2A (-9.3); HSD11B1 (-10.1); PPARD (-9.2)
80	Benzo[c]phenanthridine	*Toddalia asiatica*	AKR1B1 (-10.9); GCK (-9.0); PPARD (-9.2); PPARG (-9.5); RBP4 (-11.0); RXRA (-9.5)
81	Berberine	*Argemone Ochroleuca*	AKR1B1 (-9.1); HSD11B1 (-9.3); PPARD (-9.0)
82	Beta-amyrin	*Artemisia afra, Euphorbia hirta, Impomoea pes-caprae ssp. Brasiliensis*	AMY2A (-9.7); HSD11B1 (-9.7); PTPN9 (-9.0)
83	Beta-amyrin acetate	*Impomoea pes-caprae ssp. Brasiliensis*	AMY2A (-9.8)
84	Beta-glochidone	*Impomoea pes-caprae ssp. Brasiliensis*	AMY2A (-10.8); DPP4 (-9.6); HSD11B1 (-11.2); PTPN9 (-9.0)
85	Betulinic acid	*Adansonia digitata, Cajanus cajan, Harungana madagascariensis*	AMY2A (-9.1); PTPN9 (-9.3)
86	Biochanin A	*Cajanus cajan*	AKR1B1 (-9.2); FFAR1 (-9.2)

87	bisabolene	*Xylopia aethiopica*	AKR1B1 (-9.1)
88	biscryptolepine	*Cryptolepis sanguinolenta*	AMY2A (-10.5); DPP4 (-10.5); FFAR1 (-9.1); GCK (-9.9); INSR (-9.2); PDK2 (-9.7); PPARA (-11.4); PPARD (-9.2); PPARG (-11.5)
89	Bovogenin A	*Bowiea volubilis*	HSD11B1 (-10.2); PPARD (-9.8); PPARG (-10.1)
90	Bovoside A	*Bowiea volubilis*	AMY2A (-9.2); PPARD (-9.7); PPARG (-9.0)
91	brassinolide	*Catharanthus roseus*	AKR1B1 (-9.8); HSD11B1 (-10.2); PPARD (-9.4); PPARG (-9.3)
92	Bulbispermine	*Crinum bulbispermum*	HSD11B1 (-9.0)
93	Buphanisin	*Boophane disticha*	AKR1B1 (-9.4); GCK (-9.3)
94	Cajaquinone	*Cajanus cajan*	RBP4 (-9.4)
95	Calycosin	*Cyclopia spp*	AKR1B1 (-9.4); FFAR1 (-9.0)
96	Campesterol	*Adansonia digitata, Euphorbia Hirta, Prunus africana*	AKR1B1 (-9.5); AMY2A (-9.1); DPP4 (-9.4); FFAR1 (-9.2); HSD11B1 (-11.0); MGAM (-9.0); PPARD (-9.7); PPARG (-9.9)
97	Caranine	*Amaryllis belladonna*	RBP4 (-9.7)
98	Carapanaubine	*Rauvolfia vomitoria*	PPARD (-9.4)
99	Castalagin	*Quercus robur*	AMY2A (-9.0); DPP4 (-10.4); PDK2 (-9.0); PYGL (-9.1)
100	Catechin	*Adansonia digitata, Combretum micranthum, Pelargonium sidoides, Prunus africana, Sclerocarya birrea, Typha capensis*	AKR1B1 (-9.0); HSD11B1 (-9.5); RBP4 (-9.3)
101	Celastrin	*Gunnera perpensa*	PPARD (-9.9)
102	Centelloside	*Centella asiatica*	AMY2A (-9.3); DPP4 (-9.9)
103	Chelerythrine	*Toddalia asiatica*	AKR1B1 (-9.5); DPP4 (-9.0); GCK (-9.1); HSD11B1 (-9.4); PPARG (-9.0)
104	Chironioside	*Chironia baccifera*	HSD11B1 (-9.6)
105	Chelerythrine-psi-cyanide	*Toddalia asiatica*	HSD11B1 (-9.2); PPARD (-9.1)
106	Chlorogenic acid	*Euphorbia hirta, Moringa oleifera, Scabiosa columbaria, Trichilia emetica, Vernonia amygdalina*	AKR1B1 (-9.1)
107	Christyoside	*Strophanthus speciosus*	PPARD (-10.1)
108	Chrysophonein	*Rumex lanceolatus*	PPARG (-9.1)

109	Chrysophonol	*Bulbine frutescens, Harungana madagascariensis, Rumex lanceolatus*	RBP4 (-9.8)
110	Cissacapine	*Cissampelos capensis*	AMY2A (-11.0); DPP4 (-10.1); NR5A2 (-9.1); PYGL (-9.1)
111	Cissampreine	*Cissampelos capensis*	AMY2A (-9.6); DPP4 (-9.6)
112	Cliviamartine	*Clivia miniata*	AKR1B1 (-9.4); AMY2A (-9.1); DPP4 (-10.1); GCK (-10.9); HSD11B1 (-10.1); NR5A2 (--9.3); PPARA (-9.4); PPARD (-11.2); PPARG (-9.8)
113	Cliviasine	*Clivia miniata*	HSD11B1 (-9.2); PPARG (-9.2)
114	Clividine	*Clivia miniata*	HSD11B1 (-9.2); PPARG (-9.0)
115	Clivimine	*Clivia miniata*	AKR1B1 (-11.1); AMY2A (-11.5); DPP4 (-11.7); FFAR1 (-10.0); GCK (-11.5); INSR (-9.6); MGAM (-9.1); NR5A2 (-12.2); PDK2 (-11.5); PPARA (-9.3); PPARD (-13.7); PPARG (-12.9); PYGL (-10.5); RXRA (-10.1)
116	Clivonine	*Clivia miniata*	GCK (-9.0); HSD11B1 (-9.5); RBP4 (-10.1)
117	Combretin A	*Combretum micranthum*	GCK (-9.2); HSD11B1 (-9.6); PPARD (-9.5)
118	Combretin B	*Combretum micranthum*	DPP4 (-9.2)
119	Cornustannin 2	*Curtisia dentata*	AMY2A (-9.9); DPP4 (-10.8); PDK2 (-9.4)
120	Corosolic acid	*Prunus africana*	AMY2A (-9.2); DPP4 (-9.9); HSD11B1 (-9.7); PDK2 (-9.1); PTPN9 (-9.0)
121	Cotyledoside	*Tylecodon wallichii*	AMY2A (-9.4); DPP4 (-9.5); PTPN9 (-9.5)
122	Crinamine	*Crinum bulbispermum, Crinum macowanii*	GCK (-9.4)
123	Crinasiadine	*Crinum bulbispermum*	AKR1B1 (-9.5); RBP4 (-9.9)
124	Crinasiatine	*Crinum bulbispermum*	AKR1B1 (-10.8); AMY2A (-9.0); DPP4 (-9.8); FFAR1 (-9.9); GCK (-10.0); HSD11B1 (-11.0); PPARA (-10.9); PPARD (-10.6); PPARG (-10.9)
125	Crotofolin A	*Croton Gratissimus*	AMY2A (-9.2); HSD11B1 (-9.9); PPARD (-9.3)
126	Crotonin	*Croton Gratissimus*	PPARD (-9.4)
127	Cryptogenin	*Balanites maughami*	AMY2A (-9.0); HSD11B1 (-10.0); PPARD (-9.8); RBP4 (-10.0)

128	Cryptoheptine	*Cryptolepis sanguinolenta*	GCK (-9.3); HSD11B1 (-9.1); RBP4 (-10.3)
129	Cryptolepine	*Cryptolepis sanguinolenta*	PPARG (-9.3); RBP4 (-10.1)
130	Cryptomisrine	*Cryptolepis sanguinolenta*	AKR1B1 (-11.1); AMY2A (-11.3); DPP4 (-11.7); FFAR1 (-9.3); GCK (-13.0); HSD11B1 (-12.8); INSR (10.8); MGAM (-10.0); NR5A2 (-11.5); PDK2 (-10.2); PPARA (-9.5); PPARD (-14.3); PPARG (-11.7); PTPN9 (-10.0); PYGL (-10.5)
131	Cryptoquindoline	*Cryptolepis sanguinolenta*	AKR1B1 (-9.8); AMY2A (-11.4); DPP4 (-11.2); FFAR1 (-9.3); GCK (-11.1); HSD11B1 (-12.0); INSR (-10.6); MGAM (-10.0); NR5A2 (-10.0); PDK2 (-10.1); PPARA (-11.3); PPARD (-11.2); PPARG (-11.9); PYGL (-9.1); RXRA (-9.5)
132	Cryptospirolepine	*Cryptolepis sanguinolenta*	AMY2A (-10.8); DPP4 (-11.8); FFAR1 (-9.5); GCK (-12.4); INSR (-10.9); MGAM (-9.7); PDK2 (-9.6); PPARD (-9.2); PTPN9 (-10.2); PYGL (-10.2)
133	Cucurbitacin B	*Cucumis africanus*	HSD11B1 (-9.0); PPARD (-9.1); PPARG (-9.8)
134	Curcuson A	*Jatropha curcas*	AKR1B1 (-9.6); GCK (-9.7); PPARD (-9.7); RBP4 (-10.0); RXRA (-9.1)
135	Curcuson C	*Jatropha curcas*	AKR1B1 (-9.6); RBP4 (-9.7)
136	Cyanidin	*Rhoicissus tridentata*	AKR1B1 (-9.1); HSD11B1 (-9.5); RBP4 (-9.2)
137	Cyclamin	*Cyclamen Persicum*	AMY2A (-9.0); DPP4 (-10.9)
138	Cycloartenol	*Ravanela madagascariensis*	AMY2A (-9.7); FFAR1 (-9.3); GCK (-9.7); HSD11B1 (-11.1); PPARD (-9.7); PPARG (-9.7)
139	Cynafoside B	*Cynanchum africanum*	PDK2 (-9.4); PPARG (-9.3)
140	Daucosterol	*Prunus africana*	DPP4 (-9.5); HSD11B1 (-10.2); PPARD (-9.8); PPARG (-9.9)
141	Deacetyldesformoakuammilin	*Rauvolfia vomitoria*	HSD11B1 (-9.1); PPARD (-9.0)
142	Deguelin	*Mundulea sericea*	AKR1B1 (-9.5); AMY2A (-9.0); DPP4 (-9.7); HSD11B1 (-10.5); PPARD (-9.6); PPARG (-10.6)
143	Delphinidin	*Hibiscus sabdariffa, Rhoicissus tridentata, Syzygium Cordatum*	AKR1B1 (-9.0); HSD11B1 (-9.6); RBP4 (-9.1)

144	Delta-7-avenasterol	*Ravanela madagascariensis*	HSD11B1 (-10.7); PPARD -10.4)
145	Deltonin	*Dioscorea dregeana*	AMY2A (-9.6); DPP4 (-10.5)
146	Deltoside	*Dioscorea dregeana*	DPP4 (-9.0)
147	Des-N-methylchelerythrine	*Toddalia asiatica*	AKR1B1 (-9.4); HSD11B1 (-9.4); PPARG (-9.4)
148	Dicoumarol	*Melilotus alba*	AKR1B1 (-11.5); DPP4 (-9.4); GCK (-10.1); HSD11B1 (-9.5); PPARD (9.6); PPARG (-10.4); RBP4 (-9.1); RXRA (-9.8)
149	Digallic acid	*Quercus robur*	AKR1B1 (-9.2)
150	Digitoxin	*Digitalis purpurea*	AMY2A (-10.7); DPP4 (-9.7); GCK (-10.3); PPARD (-9.0); PPARG (-9.8); PTPN9 (-9.8); PYGL (-9.0)
151	Dihydrosanguinarine	*Eschscholzia californica*	AKR1B1 (-10.6); DPP4 (-9.2); GCK (-9.5); HSD11B1 (-10.3); INSR (-9.2); PPARD (-9.7); PPARG (-9.8)
152	Dioscin	*Dioscorea dregeana*	AMY2A (-9.9); DPP4 (-10.3)
153	Diosgenin	*Balanites aegyptiavus, Balanites maughamii, Dioscorea dregeana*	AKR1B1 (-9.5); NR5A2 (-9.0); PPARD (-12.0)
154	Diosmetin	*Agathosma betulina*	AKR1B1 (-9.1); HSD11B1 (-9.1); RBP4 (-9.5); RXRA (-9.0)
155	Diosmin	*Agathosma betulina*	GCK (-9.0); HSD11B1 (-9.3); PPARD (-9.2); PPARG (-9.4)
156	Diospyrin	*Euclea undulata*	AKR1B1 (-9.4); AMY2A (-9.7); DPP4 (-10.4); FFAR1 (-9.0); HSD11B1 (-10.5); MR5A2 (-9.2); PPARD (-10.4); PPARG (-10.4)
157	Durantoside I	*Duranta erecta*	PPARD (-9.3)
158	Durantoside II	*Duranta erecta*	DPP4 (-9.4); HSD11B1 (-9.6); MGAM (-9.2); PPARD (-9.1); PPARG (-9.5)
159	Durantoside III	*Duranta erecta*	PPARD (-9.3); PPARG (-9.2)
160	Eburnamonine	*Vinca minor*	AKR1B1 (-9.4); HSD11B1 (-9.2); PPARD (-9.3); RBP4 (-9.4)
161	Elaeocyanidin	*Cassine transvaalensis*	HSD11B1 (-9.3); PPARG (-9.1)
162	Ent-16-Kauren-18-oic-acid	*Aster bakeranus*	AMY2A (-9.0); HSD11B1 (-9.1)

163	Ent-16-Kauren-19-oic-acid	*Aster bakeranus*	HSD11B1 (-9.4); PPARD (-9.4)
164	Epicatechin	*Acacia karroo, Harungana madagascariensis, Prunus africana*	AKR1B1 (-9.2); RBP4 (-9.3)
165	Epifriedelinol	*Syzygium cordatum*	HSD11B1 (-11.4)
166	Epimaslinic acid	*Prunus africana*	AMY2A (-9.7); DPP4 (-9.0)
167	Ergine	*Ipomoea purpurea*	HSD11B1 (-9.2); RBP4 (-9.4)
168	Eriodictyol	*Cyclopia spp*	HSD11B1 (-9.2); RBP4 (-9.5)
169	Erythraline	*Erythrina caffra, Erythrina lysistemon*	AKR1B1 (-9.0); GCK (-9.8); RBP4 (-9.0)
170	Erythrophleine	*Erythrophleum lasianthum*	AKR1B1 (-9.0); HSD11B1 (-9.4)
171	Eucosterol	*Eucomis autumnalis*	DPP4 (-9.3); PPARD (-9.7)
172	Flemichapparin C	*Cyclopia spp*	AKR1B1 (-10.0); DPP4 (-9.1); GCK (-9.2); HSD11B1 (-9.3); PPARD (-9.2); RBP4 (-9.4)
173	Formononetin	*Cyclopia spp*	AKR1B1 (-9.4)
174	Friedelin	*Adansonia digitata, Artemisia afra, Aster bakeranus, Euphorbia hirta, Harungana madagascariensis, Prunus africana, Syzygium Cordatum*	AMY2A (-9.2); DPP4 (-9.8); HSD11B1 (-10.5); NR5A2 (-9.6); PPARD (-9.5); PYGL (-9.0)
175	Frutinone A	*Polygala fruticosa*	AKR1B1 (-9.5); GCK (-9.4); HSD11B1 (-9.1); PPARD (-9.6); PPARG (-9.5); RBP4 (-10.5); RXRA (-9.0)
176	Fujikinetin	*Cyclopia spp*	AKR1B1 (-10.2); FFAR1 (-9.0); PPARG (-9.0); RBP4 (-9.8)
177	Furanoeudesma-1,3-diene	*Commiphora myrrha*	RBP4 (-9.0)
178	Gaboroquinone A	*Bulbine frutescens*	AMY2A (-9.0)
179	Gaboroquinone B	*Bulbine frutescens*	HSD11B1 (-9.1); PPARD (-9.40
180	Galanthamine	*Crinum bulbispermum*	AKR1B1 (-9.1)
181	Gallocatechin	*Pelargonium sidoides*	HSD11B1 (-9.2); RBP4 (-9.0)
182	Garcifuran B	*Garcinia kola*	AKR1B1 (-9.4); RBP4 (-9.6)
183	Garcinia biflavonoid 1	*Garcinia kola*	AKR1B1 (-9.2); AMY2A (-9.2); DPP4 (-9.0); MGAM (-9.1); PPARD (-10.9)
184	Garcinia biflavonoid 2	*Garcinia kola*	AKR1B1 (-9.3); AMY2A (-9.1); DPP4 (-9.1); MGAM (-9.3); PPARD (-10.7); PYGL (-9.1)

185	Geissoschizine	*Rauvolfia vomitoria*	PPARD (-9.1)
186	Geissoschizol	*Rauvolfia vomitoria*	PPARD (-9.1)
187	Gelsemicine	*Gelsemium sempervirens*	HSD11B1 (-9.3); PPARG (-9.2)
188	Genistein	*Cajanus cajan*	AKR1B1 (-9.2); HSD11B1 (-9.0); RBP4 (-9.9)
189	Geraniin	*Geranium Incanum*	AMY2A (-9.0); DPP4 (-10.9); PDK2 (-10.1); PTPN9 (-9.3)
190	Gitogenin	*Tribulus terrestris*	AMY2A (-9.4); HSD11B1 (-9.4)
191	Glycyrrhizin	*Abrus Precatorius, Glycyrrhiza glabra*	AMY2A (-9.6); DPP4 (-10.2); PTPN9 (-9.6); PYGL (-9.4)
192	Gnidicin	*Gnidia kraussiana*	DPP4 (-10.4); FFAR1 (-9.3); GCK (-9.1); MGAM (-9.7); PDK2 (-9.7); PPARD (-12.7); PPARG (-9.3); PYGL (-9.6)
193	Gnidilatin	*Gnidia kraussiana*	DPP4 (-9.4); PPARD (-10.8)
194	Gniditrin	*Gnidia kraussiana*	AMY2A (-9.6); FFAR1 (-9.4); PPARD (-9.5)
195	Gomphoside	*Asclepias fruticosa*	AKR1B1 (-9.8); AMY2A (-10.7); DPP4 (-9.3); GCK (-10.6); KSD11B1 (-11.2); NR5A2 (-10.4); PPARG (-10.3); PTPN9 (-9.8)
196	Gossypetin	*Hibiscus sabdariffa*	HSD11B1 (-9.7); RBP4 (-9.4)
197	Grayanotoxin I	*Rhododendron indicum*	HSD11B1 (-9.4)
198	Guiajaverin	*Psidium guajava*	PPARG (-9.3)
199	Harman	*Tribulus terrestris*	AKR1B1 (-9.1)
200	Harpagoside	*Harpagophytum procumbens*	HSD11B1 (-9.0)
201	Harunganin	*Harungana madagascariensis*	AKR1B1 (-9.7); GCK (-9.1); PPARD (-10.3)
202	Harunganol B	*Harungana madagascariensis*	AKR1B1 (-11.7); DPP4 (-9.1); GCK (-9.5); HSD11B1 (-9.7); PPARD (-9.8); PPARG (-9.1)
203	Harunmadagascarin A	*Harungana madagascariensis*	AKR1B1 (-10.5); AMY2A (-9.4); DPP4 (-10.0); GCK (-9.2); HSD11B1 (-10.8); PPARD (-11.0); PPARG (-9.0)
203	Harunmadagascarin B	*Harungana madagascariensis*	AMY2A (-9.0); DPP4 (-9.0); GCK (-9.3); HSD11B1 (-9.6); PPARD (-11.2); PPARG (-9.5)
205	Hautriwaic acid	*Dodonaea Angustifolia*	AKR1B1 (-9.3)
206	Haemanthamine	*Scadoxus puniceus*	AKR1B1 (-9.5); GCK (-9.2)

207	Haemanthidine	*Scadoxus puniceus*	AKR1B1 (-9.1)
208	Hederagenin	*Hedera helix*	DPP4 (-9.8); PTPN9 (-9.0)
209	Helichrysetin	*Helichrysum Species*	FFAR1 (-9.8)
210	Hellebrigenin	*Kalanchoe lanceolata*	PPARD (-9.3)
211	Hellebrigenin-3-acetate	*Melianthus comosus*	AMY2A (-9.0); GCK (-9.7); HSD11B1 (-9.6); PPARD (-9.3)
212	Henningsiine	*Strychnos henningsii*	AMY2A (-9.1); HSD11B1 (-9.6); PPARD (-10.0); PPARG (-9.0)
213	Hesperitin	*Cyclopia spp*	FFAR1 (-9.0); HSD11B1 (-9.2); RBP4 (-9.3)
214	Hibiscetin	*Hibiscus sabdariffa*	HSD11B1 (-9.7); RBP4 (-9.3)
215	Hibiscin	*Hibiscus sabdariffa*	HSD11B1 (-10.2)
216	Hibiscitrin	*Hibiscus sabdariffa*	HSD11B1 (-9.5); PPARG (-9.0)
217	Hippeastrine	*Clivia miniata, Crinum bulbispermum*	AKR1B1 (-10.9); HSD11B1 (-9.3)
218	Hircinol	*Dioscorea dregeana*	RBP4 (-9.1)
219	Hydroxyvernolide	*Vernonia colorata*	HSD11B1 (-9.1); PPARD (-9.1)
220	Hyoscyamine	*Datura stramonium*	AKR1B1 (-9.0)
221	Hyperforin	*Hypericum perforatum*	PPARD (-11.1)
222	Hypericin	*Harungana madagascariensis, Hypericum aethiopicum, Hypericum perforatum*	AMY2A (-11.1); DPP4 (-10.7); GCK (-9.7); INSR (-10.5); MGAM (-9.8); NR5A2 (-10.0); PPARD (-9.3); PPARG (-9.3); PTPN9 (-9.7)
223	Hyperin	*Hypericum perforatum*	AKR1B1 (-9.0); PPARD (-9.3)
224	Hypoxoside	*Hypoxis hemerocallidea*	DPP4 (-9.1); PPARG (-9.0)
225	Ibogaine	*Voacanga africana*	HD11B1 (-9.1); PPARD (-9.0)
226	Ibogamine	*Voacanga africana*	HSD11B1 (-9.1); PPARD (-9.3); RBP4 (-10.6); RXRA (-9.3)
227	Iboxygaine	*Voacanga africana*	HSD11B1 (-9.4); PPARD (-9.0)
228	Ibozol	*Tetradenia Riparia*	GCK (-9.7)
229	Icterogenin	*Lippia Rhenmannii*	DPP4 (-9.1)
230	Indole-3-acetyl aspartic acid	*Griffonia simplicifolia*	AKR1B1 (-9.0)
231	Ingenol	*Euphorbia hirta, Euphorbia ingens*	PPARD (-9.4)
232	Ingenol triacetate	*Euphorbia hirta*	PPARD (-9.1)

233	Insularine	*Cissampelos capensis*	AMY2A (-9.0); DPP4 (-9.4); MGAM (-9.1); PPARG (-9.1); PYGL (-9.1)
234	Integerrimine	*Lotononis laxa*	HSD11B1 (-9.1); PPARD (-9.3)
235	Isocryptolepine	*Cryptolepis sanguinolenta*	RBP4 (-9.9); RXRA (-9.0)
236	Isofucosterol	*Adansonia digitata*	AKR1B1 (-10.9); HSD11B1 (-10.3); PPARG (-9.0)
237	Isokigelinol	*Kigelia africana*	AMY2A (-9.6); GCK (-9.1); HSD11B1 (-10.1); NR5A2 (-9.0); PPARD (-9.6)
238	Isoorientin	*Aspalathus linearis*	AKR1B1 (-9.8); AMY2A (-9.0); DPP4 (-9.4); HSD11B1 (-9.9); PPARG (-10.2)
239	Isopinnatal	*Kigelia africana*	NR5A2 (-9.3); PPARD (-9.6)
240	Isoquercetin	*Centella asiatica, Impomoea pes-caprae ssp. Brasiliensis, Moringa oleifera*	AKR1B1 (-9.0); PPARD (-9.3)
241	Isoreserpiline	*Rauvolfia vomitoria*	PPARD (-9.1)
242	Isoreserpine	*Rauvolfia vomitoria*	GCK (-9.3)
243	Isorhamnetin	*Cajanus cajan*	HSD11B1 (-9.1); RBP4 (-9.2)
244	Isosakuranetin	*Cyclopia spp*	HSD11B1 (-9.0); RBP4 (-9.8)
245	Isovitexin	*Combretum micranthum*	DPP4 (-9.5); HSD11B1 (-9.6); PPARG (-10.0)
246	Ivangustine	*Eriocephalus africanus*	RBP4 (-9.9)
247	Kaempferol	*Euphorbia hirta, Harpephyllum Caffrum, Moringa oleifera, Rauvolfia vomitoria*	HSD11B1 (-9.0); RBP4 (-9.5)
248	Kaempferol-3-glucoside	*Moringa oleifera*	AKR1B1 (-9.3); HSD11B1 (-9.3); PPARD (-9.6)
249	Kaempferol-3-o-(6'-malonyl-glucoside)	*Moringa oleifera*	DPP4 (-9.1); HSD11B1 (-9.8); PPARD (-9.1); PPARG (-9.0)
250	Kaempferol-3-O-rhamnodiglucoside	*Danais fragrans*	DPP4 (-10.0); HSD11B1 (-9.0); PPARD (-9.4); PPARG (-9.4)
251	Kauran-16a-ol	*Xylopia aethiopica*	HSD11B1 (-9.4)
252	Kigelinol	*Kigelia africana*	HSD11B1 (-9.1); PPARD (-9.4)
253	Kigelinone	*Kigelia africana*	AKR1B1 (-9.1); RBP4 (-9.4)
254	Knipholone	*Bulbine frutescens, Bulbine natalensis*	AKR1B1 (-9.7); HSD11B1 (-9.8); PPARD (-9.2); PPARG (-9.5)
255	Kolaflavanone	*Garcinia kola*	AMY2A (-9.0); DPP4 (-9.2); MGAM (-9.0); PPARD (-10.9)

256	Kolanone	*Garcinia kola*	AKR1B1 (-11.5); GCK (-10.3); HSD11B1 (-9.4); PPARD (-10.0); PPARG (-9.8)
257	Lactucin	*Chicorium intybus*	RBP4 (-9.4)
258	Lanceotoxin A	*Kalanchoe lanceolata*	PPARD (-9.1)
259	Lantadene A	*Lantana camara, Lippia Rehmannii*	AMY2A (-9.0); DPP4 (-9.5); HSD11B1 (-9.6); INSR (-9.0)
260	Lantadene B	*Lantana camara*	PTPN9 (-9.0)
261	Lapachol	*Kigelia africana*	AKR1B1 (-9.2)
262	Lemmatoxin	*Phytolacca dodecandra*	DPP4 (-10.3)
263	Leucocyanidin	*Euphorbia hirta*	RBP4 (-9.2)
264	Leurosidine	*Catharanthus roseus*	DPP4 (-9.0)
265	Leurosine	*Catharanthus roseus*	DPP4 (-9.1)
266	Ligustroside	*Ligustrum lucidum*	HSD11B1 (-9.3); PPARG (-9.4)
267	Liquiritin	*Glycyrrhiza glabra*	FFAR1 (-9.1); HSD11B1 (-9.1); PPARG (-9.0)
268	Lithospermic acid	*Echium vulgare*	AKR1B1 (-9.0); DPP4 (-9.0); GCK (-9.4); HSD11B1 (-9.6); PPARD (-9.8); PPARG (-11.0); PYGL (-9.0)
269	Lochnerine	*Catharanthus roseus*	DPP4 (-9.5); PPARD (-9.0)
270	Longistylin A	*Cajanus cajan*	AKR1B1 (-9.6); DPP4 (-9.0); FFAR1 (-9.8); GCK (-9.0); PPARG (-9.0)
271	Longistylin C	*Cajanus cajan*	AKR1B1 (-10.0); PPARD (-9.3); RBP4 (-9.8); RXRA (-9.5)
272	Lupeol	*Catharanthus roseus*	HSD11B1 (-9.8); PPARD (-9.8)
273	Luteolin	*Kigelia africana, Vernonia amygdalina*	AKR1B1 (-9.2); HSD11B1 (-9.5); RBP4 (-10.0); RXRA (-9.2)
274	Luteolin-4-rutinoside	*Vernonia amygdalina*	AMY2A (-9.4); DPP4 (-9.9); FFAR1 (-9.1); HSD11B1 (-9.8); PPARD (-10.4); PPARG (-10.1)
275	Luteolin-7-glucoside	*Vernonia amygdalina*	DPP4 (-9.3); HSD11B1 (-9.6); PPARD (-9.6); PPARG (-10.0)
276	Luteolin-7-rutinoside	*Vernonia amygdalina*	AKR1B1 (-9.1); AMY2A (-9.7); DPP4 (-10.5); GCK (-9.8); HSD11B1 (-9.8); INSR (-9.4); PPARD (-9.8); PPARG (-11.4); PTPN9 (-9.8); PYGL (-9.4)

277	Lycorine	*Amaryllis belladonna, Clivia miniata, Crinum bulbispermum, Crinum macowanii*	AKR1B1 (-9.0); HSD11B1 (-9.1); RBP4 (-9.7)
278	Madecassic acid	*Centella asiatica*	AMY2A (-9.1); DPP4 (-9.1); HSD11B1 (-9.1)
279	Maslinic acid	*Prunus africana*	AMY2A (-9.7); DPP4 (-9.0)
280	Medicagol	*Cyclopia spp*	AKR1B1 (-9.4); GCK (-9.5); HSD11B1 (-9.4); PPARD (-9.4); PPARG (-9.4); RBP4 (-10.1)
281	Melianol	*Melia azedarach*	AMY2A (-9.3); DPP4 (-9.2); HSD11B1 (-9.0); PTPN9 (-9.1); PYGL (-9.0)
282	Melianthugenin	*Bersama lucens, Melianthus comsus*	AKR1B1 (-9.8); AMY2A (-9.5); DPP4 (-9.1); GCK (-9.0); HSD11B1 (-9.6); PDK2 (-9.1); PPARD (-10.3); PPARG (-10.3)
283	Meliatoxin B1	*Melia azedarach*	AKR1B1 (-10.1); DPP4 (-9.7)
284	Meliatoxin B2	*Melia azedarach*	AKR1B1 (-10.3); DPP4 (-10.0)
285	Mucronine D	*Ziziphus mucronata*	AMY2A (-9.1); DPP4 (-11.4); GCK (-10.3); HSD11B1 (-10.3); NR5A2 (-10.0); PPARD (-10.0); PPARG (-10.3)
286	Naringenin	*Cyclopia spp.*	HSD11B1 (-9.0); RBP4 (-9.8)
287	Nauclefidine	*Nauclea latifolia*	AKR1B1 (-10.1); HSD11B1 (-9.0); RBP4 (-10.0)
288	Naucletine	*Nauclea latifolia*	AKR1B1 (-9.3); GCK (-10.5); HSD11B1 (-10.2); PPARD (-9.9); PPARG (-10.50; PTPN9 (-9.0); PYGL (-9.0)
289	Neocryptolepine	*Cryptolepis sanguinolenta*	AKR1B1 (-9.6); PPARD (-9.1); RBP4 (-10.3); RXRA (-9.5)
290	N-methylflindersine	*Toddalia asiatica*	AKR1B1 (-9.2); RBP4 (-9.5)
291	Norajmaline	*Rauvolfia vomitoria*	HSD11B1 (-9.1); PPARD (-9.6)
292	Nothofagin	*Aspalathus linearis*	AKR1B1 (-9.8); FFAR1 (9.7); RXRA (-9.0)
293	Nudicauline	*Delphinium grandiflorum*	AMY2A (-9.0); DPP4 (-9.8); PPARD (-10.3)
294	Obebioside	*Adenium multiflorum*	DPP4 (-9.8); PTPN9 (-9.2)
295	Ocubullenone	*Ocotea bullata*	AKR1B1 (-9.5)
296	Oleacein	*Olea europaea*	AKR1B1 (-9.1); FFAR1 (-9.1)
297	Oleandrin	*Nerium oleander*	HSD11B1 (-9.9); PPARD (-9.0)
298	Oleanoglycotoxin	*Phytolacca dodecandra*	DPP4 (-9.7)

299	Oleanolic acid	*Ligustrum lucidum, Melianthus comosus, Prunus africana*	DPP4 (-9.6); HSD11B1 (-10.7)
300	Oleanolic-3-acetate	*Prunus africana*	AMY2A (-9.1)
301	Oleuropein	*Ligustrum lucidum, Olea europaea*	HSD11B1 (-9.0)
302	Orbicuside A	*Cotyledon orbiculata*	AMY2A (-10.9); DPP4 (-9.3); PTPN9 (-9.7); PYGL (-9.3)
303	Orientin	*Aspalathus linearis*	HSD11B1 (-9.5)
304	Ouabain	*Acokanthera oppositifolia, Strophanthus gratus*	HSD11B1 (-9.9)
305	Ouratea proanthocyanidin A	*Cassine transvaalensis*	AMY2A (-9.4); PPARD (-9.1)
306	Oxyavicine	*Toddalia asiatica*	AKR1B1 (-10.0); AMY2A (-9.8); DPP4 (-9.5); GCK (-10.1); HSD11B1 (-10.6); INSR (-9.2); PPARD (-9.7); PPARG (-10.3); RXRA (-9.2)
307	Oxychelerythrine	*Toddalia asiatica*	AKR1B1 (-9.3); AMY2A (-9.1); HSD11B1 (-9.9); PPARG (-9.4)
308	P57 compound	*Hoodia gordonii*	DDP4 (-10.2); PPARG (-9.3)
309	Palmitic acid	*Hibiscus sabdariffa*	RBP4 (-9.1)
310	Parquin	*Cestrum laevigatum*	DPP4 (-9.2)
311	Peddiea Factor A1	*Peddiea africana*	PPARD (-9.5); PTPN9 (-9.1)
312	Petunidine	*Catharanthus roseus*	AKR1B1 (-9.0); HSD11B1 (-9.4); RBP4 (-9.0)
313	Picrinine	*Rauvolfia vomitoria*	AMY2A (-9.1); HSD11B1 (-9.4); PPARD (-9.6); PPARG -9.0)
314	Pinocembrin	*Tarchonanthus camphoratus*	FFAR1 (-9.0); RBP4 (-9.6)
315	Pinoresinol	*Strophanthus gratus*	AKR1B1 (-9.2); HSD11B1 (-9.0); PPARG (-9.0)
316	Pinostrobin	*Cajanus cajan*	RBP4 (-9.5)
317	Platyphylline	*Senecio serratuloides*	HSD11B1 (-9.4); PPARD (-9.3)
318	Plumbagin	*Plumbago auriculata*	FFAR1 (-9.1)
319	Pratorimine	*Crinum bulbispermum, Crinum macowanii*	AKR1B1 (-9.0); RBP4 (-10.0)
320	Premarrubiin	*Leonotis leonurus*	HSD11B1 (-9.9); NR5A2 (-9.1); PPARD (-9.7)
321	Presenegenin	*Polygala fruticosa, Securidaca longipedunculata*	HSD11B1 (-10.1)
322	Procyanidin C1	*Sclerocarya birrea*	AMY2A (-10.0); DPP4 (-10.7); GCK (-9.9); MGAM (-9.9); PYGL (-10.8)

323	Propacin	*Mondia whitei*	AMY2A (-9.0); HSD11B1 (-9.7); PPARD (-9.2); PPARG (-9.4)
324	Proscillardin A	*Drimia robusta, Scilla natalensis*	AKR1B1 (-10.0); AMY2A (-9.3); DPP4 (-10.2); HSD11B1 (-10.7); PPARD (-10.0)
325	Protopine	*Argemone ochoroleuca*	HSD11B1 (-10.1); IMSR (-9.1); PPARD (-10.4)
326	Pseudobaptigen	*Cyclopia spp*	AKR1B1 (-10.3); DPP4 (-9.1); FFAR1 (-10.0); HSD11B1 (-9.1); RBP4 (-9.8)
327	Pterygospermin	*Moringa oleifera*	AKR1B1 (-11.9); AMY2A (-9.0); DPP4 (-9.8); GCK (-11.1); HSD11B1 (-9.9); INSR (-9.2); NR5A2 (-9.8); (PPARD (-11.6); PPARG (-9.5); RXRA (-9.0)
328	Punicalagin	*Punica granatum*	DPP4 (-11.3); MGAM (-9.3); PYGL (-10.3)
329	Punicalin	*Punica Granatum*	AMY2A (-10.8); DPP4 (-10.5); PTPN9 (-10.1); PYGL (-10.5)
330	Pyrethrin I	*Chrysanthemum cinerariifolium*	AKR1B1 (-9.7); GCK (-9.5); PPARD (-9.0); PPARG (-9.1); RXRA (-9.3)
331	Quercetin	*Acacia karroo, Aspalathus linearis, Cajanus cajan, Euphorbia hirta, Harungana madagascariensis, Kigelia africana, Moringa oleifera, Pelargonium sidoides, Psidium guajava, Rauvolfia vomitoria*	HSD11B1 (-9.6); RBP4 (-9.6)
332	Quercetin-7-O-B-D-xylopyranoside	*Adansonia digitata*	HSD11B1 (-9.2); PPARG (-9.2)
333	Quercetin-3,7-diglucoside	*Agathosma betulina*	DPP4 (-9.0); HSD11B1 (-9.4); PPARD (-10.3); PPARG (-9.8)
334	Quercetin-3-o-(6'-malonyl-glucoside)	*Moringa oleifera*	HSD11B1 (-9.8); PPARD (-9.2); PPARG (-9.2)
335	Quercitrin	*Danais fragrans, Euphorbia hirta, Thesium hystrix*	AKR1B1 (-9.3); HSD11B1 (-9.2); PPARD (-9.5); PPARG (-9.2)
336	Quindoline	*Cryptolepis sanguinolenta*	AKR1B1 (-9.5); RBP4 (-9.7); RXRA (-10.1)
337	Rauvoxin	*Rauvolfia vomitoria*	PPARD (-9.4)
338	Rauvoxinin	*Rauvolfia vomitoria*	PPARD (-9.4)
339	Reserpinic acid	*Rauvolfia vomitoria*	PPARD (-9.0); PPARG (-9.3)
340	Reserpine	*Catharanthus roseus, Rauvolfia caffra, Rauvolfia, vomitoria*	DPP4 (-9.0); PPARD (-9.4); PPARG (-9.8)

341	Resveratrol	*Terminalia sericea*	AKR1B1 (-9.0); FFAR1 (-9.6)
342	Resveratrol-3-O-B-rutinoside	*Terminalia sericea*	DPP4 (-9.6); FFAR1 (-9.0); GCK (-9.3); HSD11B1 (-9.9); PPARD (-9.1); PPARG (-10.2); PYGL (-9.5)
343	Retrorsine	*Crotalaria spartioides, Senecio retrorsus*	HSD11B1 (-9.3)
344	Rhinocerotinoic acid	*Elytropappus rhinoceotis*	HSD11B1 (-9.2); RBP4 (-9.9)
345	Rohituka 3	*Trichilia emetica*	PYGL (-9.3)
346	Rooperol	*Hypoxis hemerocallidea*	AKR1B1 (-9.7); FFAR1 (-9.4); GCK (-9.6); RXRA (-9.3)
347	Rotenone	*Mundulea sericea*	AKR1B1 (-9.6); GCK (-10.1)
348	Rubiadin	*Danais fragrans*	RBP4 (-9.9)
349	Rubiadin xyloglucoside	*Danais fragrans*	AMY2A (-9.0); DPP4 (-9.8); HSD11B1 (-11.0); PPARD (-9.4); PPARG (-9.9); PTPN9 (-9.1); PYGL (-9.1)
350	Ruscogenin-(25S)-form	*Sansivieria hyancinthoides*	AMY2A (-10.1); DPP4 (-9.0); NR5A2 (-9.2)
351	Rutaretin	*Ruta graveolens*	AKR1B1 (-9.1); RBP4 (-9.0)
352	Rutin	*Acacia senegal, Agathosma betulina, Hypericum perforatum, Ruta graveolens*	DPP4 (-9.4); GCK (-9.2); HSD11B1 (-10.2); PPARD (-10.1); PPARG (-9.7); PTPN9 (-9.4)
353	Sakurasosaponin	*Rapanea melanphloeos*	AMY2A (-9.3); DPP4 (-9.8)
354	Sanguinarine	*Argemone ochroleuca, Fumaria officinalis, Zanthoxylum capense*	AKR1B1 (-10.3); AMY2A (-9.3); GCK (-9.5); HSD11B1 (-10.1); INSR (-9.2); PPARD (-10.1); PPARG (-9.9); RBP4 (-9.7)
355	Sanjoinine A	*Ziziphus mucronata*	PPARD (-11.7)
356	Sarcovimiside B	*Sarcostemma viminale*	AKR1B1 (-10.8); AMY2A (-10.0); DPP4 (-10.1); GCK (-10.3); PDK2 (-9.8); PPARD (-9.1); PPARG (-9.2)
357	Scillaren A	*Urginea maritima, Urginea sanguinea*	AMY2A (-9.3); DPP4 (-10.6); NR5A2 (-9.4); PPARD (-9.6); PTPN9 (-9.0)
358	Scillarenin	*Urginea maritima*	AKR1B1 (-10.3); DPP4 (-9.3); GCK (-10.2); HSD11B1 (-10.0); PPARD (-9.5); PPARG (-10.0)
359	Senecionine	*Lotononis laxa, Senecio serratuloides*	HSD11B1 (-10.3); PPARD (-9.4)
360	Sericic acid	*Terminalia sericea*	AMY2A (-9.7); HSD11B1 (-10.9)

361	Sericoside	*Terminalia sericea*	AMY2A (-9.3); DPP4 (-9.3)
362	Serpenticine	*Rauvolfia vomitoria*	AKR1B1 (-9.2); HSD11B1 (-9.7); PPARD (-9.4); PPARG (-9.2)
363	Serpentine	*Catharanthus roseus, Rauvolfia vomitoria*	HSD11B1 (-9.7); PPARD (-9.6)
364	Sitosterol-3-glucoside	*Pelargonium sidoides*	AMY2A (-9.4); DPP4 (-9.2); GCK (-9.2); PPARD (-9.1)
365	Sodium-en- knipholone-6-O-sulfate	*Bulbine frutescens*	PPARG (-9.1)
366	Solanine	*Solanum incanum, Solanum tuberosum*	AMY2A (-9.5); DPP4 (-9.6); PPARG (-9.5)
367	Solanocapsine	*Solanum pseudocapsicum*	AKR1B1 (-9.6); AMY2A (-9.4); DPP4 (-10.2); HSD11B1 (-10.5); NR5A2 (-9.1); PPARG (-9.1)
368	Solasodine	*Solanum incanum, Solanum nigrum*	AMY2A (-9.7); NR5A2 (-9.4)
369	Solasonine	*Solanum incanum*	AMY2A (-9.1); DPP4 (-11.0); MGAM (-9.9); PYGL (-9.7)
370	Sophoracoumestan B	*Cyclopia spp*	AKR1B1 (-9.7); GCK (-9.1); HSD11B1 (-9.5); PPARG (-9.2); RBP4 (-10.7)
371	Sophoricoside	*Styphnolobium Japonicum*	FFAR1 (-10.2); HSD11B1 (-10.1); MGAM (-9.0); PPARD (-9.3); PPARG (-9.9)
372	Stachenol	*Spirostachys africana*	AKR1B1 (-9.0); HSD11B1 (-9.3); PPARD (-9.7); PPARG (-9.7); RXRA (-9.1)
373	Stachenone	*Spirostachys africana*	AKR1B1 (-9.0); HSD11B1 (-9.3); PPARD (-9.6)
374	Stigmast-4-en-3-one	*Prunus africana*	AKR1B1 (-9.2); AMY2A (-9.4); HSD11B1 (-10.5); PPARD (-9.7)
375	Stigmasterol	*Dodonaea angustifolia, Harpagophytum procumbens, Kigelia africana, Ravenala madagascariensis*	HSD11B1 (-10.0); NR5A2 (-9.0); PPARD (-10.5)
376	Strictosamine	*Nauclea latifolia*	AMY2A (-9.0); DPP4 (-9.4); HSD11B1 (-9.9); PPARD (-9.3); PPARG (-10.7)
377	Strictosidine	*Nauclea latifolia*	GCK (-9.9); HSD11B1 (-10.8); PPARD (-9.3); PPARG (-9.7)
378	Strogoside	*Strophanthus gratus*	DPP4 (-9.4); PPARD (-9.4); PTPN9 (-9.0)
379	Strophantidin	*Strophanthus gratus*	DPP4 (-9.4); HSD11B1 (-9.6); PPARD (-9.1); PPARG (-9.8)

380	Strychnine	*Strychnos henningsii, Strychnos spinosa*	AMY2A (-9.5); GCK (-9.4); HSD11B1 (-10.2); INSR (-9.1); PPARD (-10.0); PPARG (-9.4)
381	Synaptolepis factor K1	*Synaptolepis kirkii*	AMY2A (-10.0); DPP4 (-10.1); PPARD (-10.7)
382	Synaptolepis factor K7	*Synaptolepis kirkii*	HSD11B1 (-9.3); PPARD (-9.5)
383	Taraxerol	*Euphorbia hirta*	AKR1B1 (-9.2); HSD11B1 (-10.8)
384	Tephrosin	*Mundulea sericea*	AKR1B1 (-10.0); DPP4 (-9.1); GCK (-9.6); HSD11B1 (-10.0); PPARG (-9.3)
385	Terminoic acid	*Terminalia sericea*	PPARD (-9.1)
386	Tetrahydroalstonine	*Catharanthus roseus*	PPARD (-9.7)
387	Tetrahydrocannabinol	*Cannabis sativa*	FFAR1 (-9.1); GCK (-9.2); RBP4 (-9.5); RXRA (-9.2)
388	Tetrahydroxyflavanone	*Lannea acida*	AKR1B1 (-9.0); HSD11B1 (-9.2); RBP4 (-9.4)
389	Thesinine	*Borago officinalis, Thesium minkwitzianum*	AKR1B1 (-9.3); FFAR1 (-9.7); RXRA (-9.2)
390	Thevetin A	*Thevetia peruviana*	DPP4 (-9.2); PPARD (-9.5); PPARG (-9.6)
391	Thevetin B	*Thevetia peruviana*	AMY2A (-10.1); DPP4 (-9.8); PPARD (-9.1); PPARG (-9.5)
392	Trichilin A	*Trichilia emetica*	DPP4 (-9.3)
393	Tinyatoxin	*Euphorbia hirta*	AMY2A (-9.7); DPP4 (-10.2); GCK (9.4); NR5A2 (-9.2); PDK2 (-9.1); PPARD (-10.9); PPARG (-9.4); PTPN9 (-9.0)
394	Toddalosin	*Toddalia asiatica*	AMY2A (-9.5); HSD11B1 (-9.3); PPARD (-9.9)
395	Toddasin	*Toddalia asiatica*	AMY2A (-9.9); HSD11B1 (-9.1); PPARD (-9.6); PPARG (-10.1); PYGL (-9.1)
396	Trachelogenin	*Cnicus benedictus*	AKR1B1 (-9.2)
397	Tyledoside C	*Cotyledon orbiculata*	AMY2A (-9.1); DPP4 (-9.1); PTPN9 (-9.6)
398	Typhaphtalide	*Typha capensis*	AKR1B1 (-9.1); FFAR1 (-10.1); RBP4 (-9.6)
399	Typharin	*Typha capensis*	AKR1B1 (-10.2); DPP4 (-9.5); FFAR1 (-11.6); GCK (-9.4); PPARD (-9.2); RBP4 (-9.6)
400	Typhasterol	*Typha capensis*	HSD11B1 (-9.3); PPARD (-9.4)
401	Ursolic acid	*Catharanthus roseus, Ligustrum lucidum, Prunus africana, Rauvolfia vomitoria*	AMY2A (-9.5); DPP4 (-9.8); HSD11B1 (-11.5); PPARG (-9.4)
402	Urushiol III	*Hyaenanche globosa*	RBP4 (-9.1)

403	Uzarigenin	*Xysmalobium undulatum*	AKR1B1 (-9.2); AMY2A (-9.1); GCK (-9.0); HSD11B1 (-10.3); PPARD (-9.6); PPARG (-9.1)
404	Uzarin	*Xysmalobium undulatum*	PPARD (-10.3)
405	Valerenic acid	*Valeriana capensis*	AKR1B1 (-9.0)
406	Vellosimine	*Rauvolfia vomitoria*	AKR1B1 (-9.8); GCK (-9.2); HSD11B1 (-9.8); PPARD (-9.0); PPARG (-9.0)
407	Vermeerin	*Geigeria ornativa*	RBP4 (-9.8)
408	Verminoside	*Kigelia africana*	AKR1B1 (-9.4); AMY2A (-9.0); GCK (-9.5); HSD11B1 (-9.9)
409	Vernolide	*Vernonia amygdalina, Vernonia colorata*	HSD11B1 (-9.6); PPARD (-9.3)
410	Vernomygdin	*Vernonia amygdalina*	HSD11B1 (-9.6); PPARD (-9.4)
411	Vernonioside A1	*Vernonia amygdalina*	AMY2A (-9.0); DPP4 (-9.6); PPARD (-9.6); PYGL (-9.4)
412	Vernonioside A3	*Vernonia amygdalina*	AMY2A (-9.7); DPP4 (-10.7); HSD11B1 (-10.5)
413	Vernonioside B1	*Vernonia amygdalina*	DPP4 (-9.9); PDK2 (-9.6)
414	Vernonioside B2	*Vernonia amygdalina*	DPP4 (-10.2)
415	Vernonioside B3	*Vernonia amygdalina*	DPP4 (-9.8); PDK2 (-9.7)
416	Vinburnine	*Voacanga africana*	AKR1B1 (-9.6); HSD11B1 (-9.1); PPARD (-9.3); PPARG (-9.4); RBP4 (-10.7); RXRA (-9.3)
417	Vincamine	*Vinca minor*	PPARD (-9.1)
418	Vindoline	*Catharanthus roseus*	PPARD (-9.2)
419	Vitexin	*Combretum micranthum*	HSD11B1 (-9.1)
420	Voacamine	*Voacanga africana*	DPP4 (-9.4)
421	Voacangine	*Voacanga africana*	HSD11B1 (-9.3); PPARD (-9.3)
422	Voacorine	*Voacanga africana*	DPP4 (-9.3)
423	Voaphylline	*Voacanga africana*	AMY2A (-9); DPP4 (-9.6); GCK (-9.1); HSD11B1 (-9.3); PPARD (-9.1); PPARG (-9.8); RBP4 (-9.2)
424	Vobtusine	*Voacanga africana*	AMY2A (-9.7); DPP4 (-10.0); MGAM (-9.5); PTPN9 (-9.5); PYGL (-9.1)
425	Withaferin A	*Withania somnifera*	AKR1B1 (-9.3); AMY2A (-9.6); DPP4 (-9.5); HSD11B1 (-11.3); PPARD (-10.1)
426	Withasomnine	*Withania somnifera*	FFAR1 (-9.1)

427	Xanthinin	*Xanthium strumarium*	AKR1B1 (-9.1)
428	Xylopic acid	*Xylopia aethiopica*	HSD11B1 (-9.2); PPARD (-9.5)
429	Yamogenin	*Balanites aegyptiacus*	AMY2A (-10.0); PPARD (-11.3)
430	Yohimbine	*Catharanthus roseus*	HSD11B1 (-9.6); PPARD (-9.0)

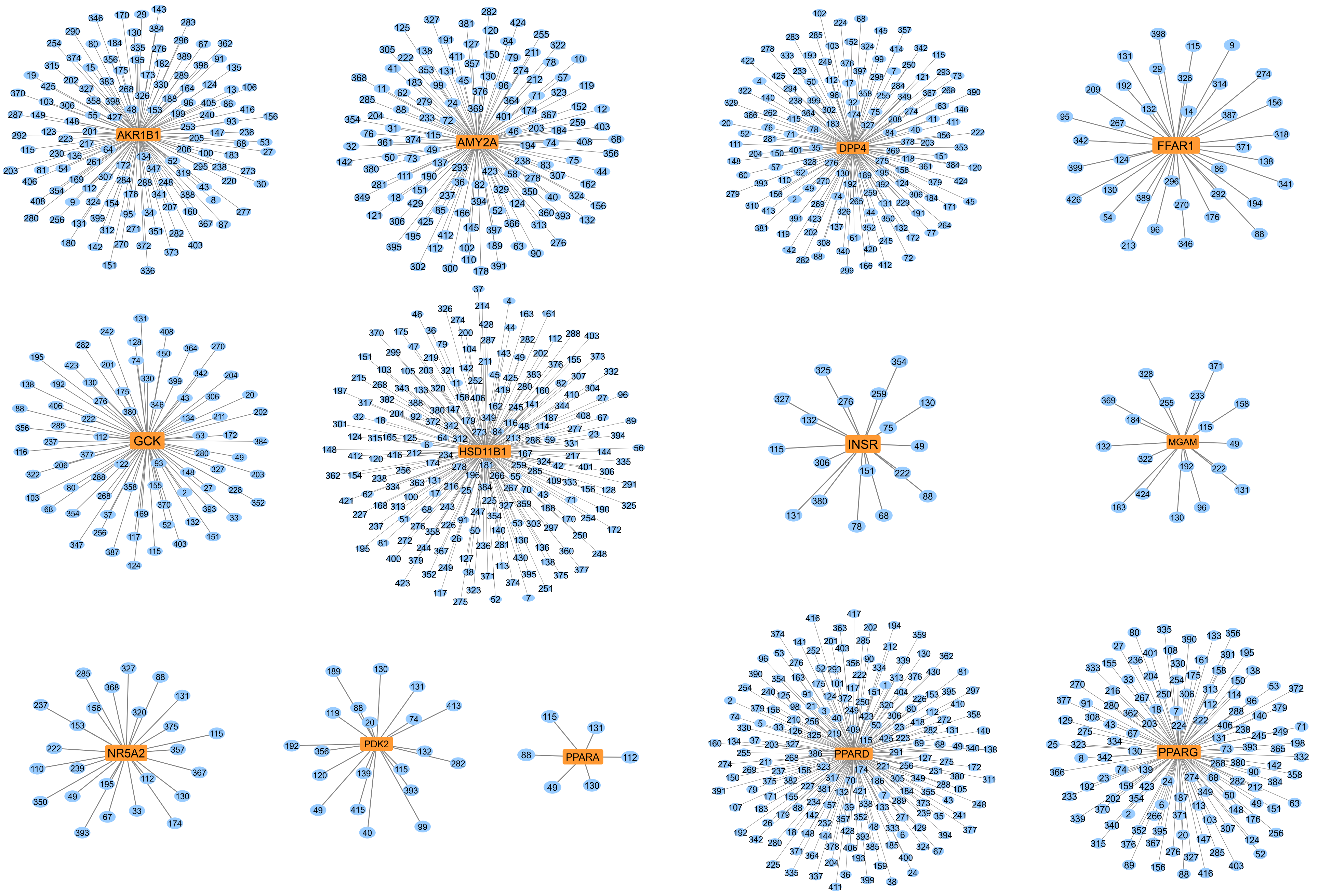
AKR1B1
AMY2A
DPP4
FFAR1
GCK
HSD11B1
INSR
MGAM
NR5A2
PDK2
PPARA
PPARD
PPARG

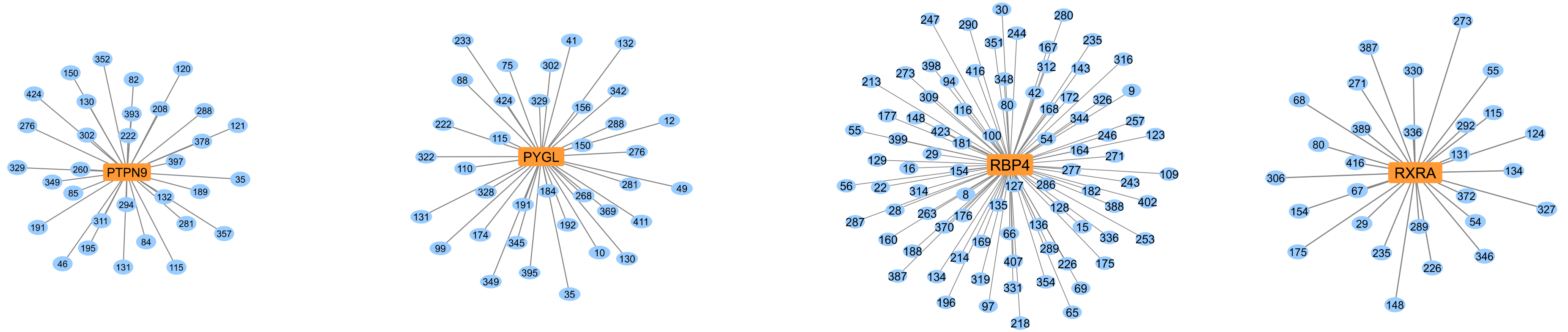

Figure S1. Individual predicted active compound-protein target networks. Compunds are represented by their assigned numerical value as in Table S2.

Table S3. Plants having scientific anti-diabetic evidence and evidence of traditional use only identified by virtual screening and their predicted bioactive compounds.

Plant name	Family	Compounds
**<u>Plants with scientific evidence</u>**		
Abrus Precatorius	Fabaceae	**Glycyrrhizin[191]**
Acacia karroo	Fabaceae	**Beta-sitosterol[70]**, **epicatechin[164]**, **quercetin[331]**
*Acacia senegal**	Fabaceae	**Quercetin[331]**
Adansonia digitata	Malvaceae	**Alpha-Linolenic acid[29]**, **avenasterol[67]**, **bauerenol[79]**, **betulinic acid[85]**, **beta-sitosterol[70]**, **campesterol[96]**, **catechin[100]**, **friedelin[174]**, isofucosterol[236], quercetin-7-O-B-D-xylopyranoside[332]
Adiantum capillus-veneris	Pteridaceae	Adiantone[33]
Aframomum melegueta	Zingiberaceae	alpha-Humulene[28]
Agathosma betulina	Rutaceae	**Diosmetin[154]**, **diosmin[155]**, quercetin-3,7-diglucoside[333], **rutin[352]**
Aloe ferox	Asphodelaceae	**Aloe-emodin anthrone[42]**, **aloeresin A[43]**, aloinoside A[44], aloinoside B[45]
*Artemisia Afra**	Asteraceae	**Alpha-amyrin[46]**, **beta-amyrin[82]**, **friedelin[174]**
Aspalathus linearis	Fabaceae	**Aspalathin[64]**, **isoorientin[238]**, **nothofagin[292]**, **orientin[303]**, **quercetin[331]**
*Balanites aegyptiacus**	Zygophyllaceae	Balagyptin[71], balanitins 1[72], 2[73], 3[74], 4[75], 6[76], 7[77], **balanitoside[78]**, **diosgenin[153]**, 6-methyldiosgenin[24], **yamogenin[429]**
Borago officinalis	Boraginaceae	Thesinine[389]
*Bulbine frutescens**	Asphodelaceae	**Chrysophonol[109]**, gaboroquinone A[178], gaboroquinone B[179], knipholone[254], Sodium ent knipholone-6-O-sulfate[365]
*Cajanus cajan**	Fabaceae	**Alpha-Linolenic acid[29]**, **apigenin[55]**, **betulinic acid[85]**, **biochanin A[86]**, cajaquinone[94], **genistein[188]**, **2-hydroxygenistein[13]**, **isorhamnetin[243]**, longistylin A[270], longistylin C[271], **pinostrobin[316]**, **quercetin[331]**
Cannabis sativa	Cannabaceae	**Tetrahydrocannabinol[387]**
Cassine transvaalensis	Celastraceae	Elaeocyanidin[161], **ouratea proanthocyanidin A[305]**
*Catharanthus roseus**	Apocynaceae	Ajmalicine[37], akuammine[39], brassinolide[91], leurosidine[264], **leurosine[265]**, **lochnerine[269]**, **lupeol[272]**, **petunidine[312]**, reserpine[340], serpentine[363], **tetrahydroalstonine[386]**, **ursolic acid[401]**, **vindoline[418]**, **yohimbine[430]**
Centella asiatica	Apiaceae	**Asiatic acid[62]**, **asiaticoside[63]**, **beta-sitosterol[70]**, centelloside[102], **isoquercetin[240]**, madecassic acid[278]
Chironia baccifera	Gentianaceae	Chironioside[104]
Cichorium intybus	Asteraceae	Lactucin[257]
Cissampelos capensis	Menispermaceae	Cissacapine[110], cissampreine[111], insularine[233]
*Cnicus benedictus**	Asteraceae	Trachelogenin[296]
Combretum micranthum	Combretaceae	**Catechin[100]**, **combretin A[117]**, **combretin B[118]**, **isovitexin[245]**, **vitexin[419]**

*Commiphora myrrha**	Burseraceae	Furanoeudesma-1-3-diene[177]
*Cryptolepis sanguinolenta**	Apocynaceae	Biscryptolepine[88], cryptoheptine[128], **cryptolepine[129]**, cryptomisrine[130], cryptoquindoline[131], cryptospirolepine[132], isocryptolepine[235], neocryptolepine[289], quindoline[336]
Curtisia dentata	Curtisiaceae	**Cornustannin-2[119]**
Cyclopia intermedia	Fabaceae	**Afrormosin[34]**, calycosin[95], **eriodictyol[168]**, flemmichapparin C[172], **formononetin[173]**, fujikinetin[176], **hesperitin[213]**, isosakuranetin[244], medicagol[280], **naringenin[286]**, pseudobaptigen[26], sophoracoumestan B[370]
Duranta erecta	Verbenaceae	Durantosides I[157], II[158], III[159]
Echium vulgare	Boraginaceae	Lithospermic acid[268]
Euclea undulata	Ebenaceae	Diospyrin[156]
*Euphorbia hirta**	Euphorbiaceae	**Alpha-amyrin[46]**, **beta-amyrin[82]**, **beta-sitosterol[70]**, **campesterol[96]**, **chlorogenic acid[106]**, **friedelin[174]**, ingenol[231], ingenol triacetate[232], **kaempferol[247]**, **leucocyanidin[263]**, **quercetin[331]**, **quercitrin[335]**, **taraxerol[383]**, tinyatoxin[393]
*Garcinia kola**	Clusiaceae	Garcifuran B[182], **garcinia biflavonoid 1[183]**, **garcinia biflavonoid 2[184]**, **kolaflavanone[255]**, kolanone[256]
Glycyrrhiza glabra	Fabaceae	**Glycyrrhizin[191]**, liquiritin[267]
Gunnera perpensa	Gunneraceae	Celastrin[101]
Harpagophytum procumbens	Pedaliaceae	**Beta-sitosterol[70]**, **harpagoside[200]**, **stigmasterol[375]**
Harpephyllum caffrum	Anacardiaceae	**Kaempferol[247]**
Harungana madagascariensis	Hypericaceae	**Betulinic acid[85]**, **chrysophonol[109]**, **epicatechin[164]**, **friedelin[174]**, **harunganin[201]**, **harunganol B[202]**, harunmadagascarin A[203], harunmadagascarin B[204], hypericin[222], **quercetin[331]**
Hedera helix	Araliaceae	**Hederagenin[208]**
Helichrysum species	Asteraceae	Alpha-humulene[28], **helichrysetin[209]**
*Hibiscus sabdariffa**	Malvaceae	**Delphinidin[143]**, gossypetin[196], hibiscetin[214], hibiscin[215], hibiscitrin[216], **palmitic acid[309]**
*Hoodia gordonii**	Apocynaceae	**P57 compound[308]**
Hypericum perforatum	Hypericaceae	**Hyperforin[221]**, hypericin[222], **hyperin[223]**, **rutin[352]**
Hypoxis hemerocallidea	Hypoxidaceae	**Beta-sitosterol[70]**, hypoxoside[224], rooperol[346]
Ipomoea pes-caprae ssp. Brasiliensis	Convolvulaceae	**Alpha-amyrin[46]**, **alpha-amyrin acetate[47]**, **beta-amyrin[82]**, beta-amyrin acetate[83], beta-glochidone[84], **isoquercetin[240]**
Jatropha curcas	Euphorbiaceae	Curcuson A[134], curcuson C[135]
Kigelia africana	Bignoniaceae	**Beta-sitosterol[70]**, 2-(1-hydroxyethyl)naphtho[2,3-beta]furan-4,9-dione[9], isokigelinol[237], isopinnatal[239], kigelinol[252], kigelinone[253], **lapachol[261]**, **luteolin[273]**, **quercetin[331]**, **stigmasterol[375]**, **verminoside[408]**
Lantana camara	Verbenaceae	Lantadene A[259], lantadene B[260]
Leonotis leonurus	Lamiaceae	Premarrubin[320]

Ligustrum lucidum	Oleaceae	Ligstroside[266], **oleanolic acid[299]**, **oleuropein[301]**, **ursolic acid[401]**
Melia azedarach	Meliaceae	Melianol[281], meliatoxin B1[283], meliatoxin B2[284]
Moringa oleifera	Moringaceae	**Chlorogenic acid[106]**, **isoquercetin[240]**, **kaempferol[247]**, **kaempferol-3-glucoside[248]**, kaempferol-3-o-(6'-malonyl-glucoside)[249], pterygospermin[327], **quercetin[331]**, quercetin-3-o-(6'-malonyl-glucoside)[334]
*Nauclea latifolia**	Rutaceae	Angustine[52], angustoline[53], nauclefidine[287], naucletine[288], strictosamine[376], strictosidine[377]
Nerium oleander	Apocynaceae	Oleandrin[297]
*Olea europaea**	Oleaceae	**8-Hydroxypinoresinol[27]**, oleacein[296], **oleanolic acid[299]**, **oleuropein[301]**
Prunus africana	Rosaceae	**Amygdalin[51]**, **beta-sitosterol[70]**, **campesterol[96]**, **catechin[100]**, **corosolic acid[120]**, **daucosterol[140]**, **epicatechin[164]**, **3-epicorosolic acid[71]**, **epimaslinic acid[166]**, esculentic acid[11], **friedelin[174]**, **maslinic acid[279]**, **oleanolic acid[299]**, oleanolic-3-acetate[300], **stigmast-4-en-3-one[374]**, **ursolic acid[401]**
Prunus laurocerasus	Rosaceae	**Amygdalin[51]**
*Psidium guajava**	Myrtaceae	Amritoside[50], guiajaverin[198], **quercetin[331]**
*Punica granatum**	Lythraceae	**Punicalagin[328]**, **punicalin[329]**
Rauvolfia caffra	Apocynaceae	Ajmalicine[37], ajmaline[38], reserpine[340]
Rauvolfia vomitoria	Apocynaceae	Ajmaline[38], carapanaubine[98], desacetyldesformoakuammilin[141], geissoschizine[185], geissoschizol[186], isoreserpiline[241], isoreserpine[242], **kaempferol[247]**, norajmaline[291], picrinine[313], **quercetin[331]**, rauvoxin[337], rauvoxinin[338], reserpic acid[339], reserpine[340], serpenticine[362], serpentine[363], **ursolic acid[401]**, vellosimine[406]
*Ravenala madagascariensis**	Strelitziaceae	**Alpha-spinasterol[48]**, **beta-sitosterol[70]**, cycloartenol[138], delta-7-avenasterol[144], **stigmasterol[375]**
Rhoicissus tridentata	Vitaceae	**Cyanidin[136]**, **delphinidin[143]**
Ruta graveolens	Rutaceae	Rutaretin[351], **rutin[352]**
*Sclerocarya birrea**	Anacardiaceae	**Catechin[100]**, **procyanidin C1[322]**
Securidaca longipedunculata	Polygalaceae	Presenegenin[321]
Solanum incanum	Solanaceae	Solanine[366], solasodine[368], solasonine[369]
Solanum nigrum	Solanaceae	Solasonine[369]
Strophanthus gratus	Apocynaceae	Acolongifloroside K[31], **8-hydroxypinoresinol[27]**, ouabain[304], **pinoresinol[315]**, strogoside[378], strophathidin[379]
Strychnos henningsii	Loganiaceae	Henningsiine[212], strychnine[380]
Styphnolobium japonicum	Fabaceae	**Sophoricoside[371]**
Syzygium cordatum	Myrtaceae	**Arjunolic acid**, **beta-sitosterol[70]**, **delphinidin[143]**, epifriedelinol[165], **friedelin[174], oleanolic acid[299]**
Tarchonanthus camphoratus	Asteraceae	Pinocembrin[314]
*Terminalia sericea**	Combretaceae	Anolignan B[54], **arjunglucoside[57]**, **resveratrol[341]**, resveratrol-3-o-b-rutinoside[342], sericic acid[360], sericoside[361], terminoic acid[385]

Toddalia asiatica	Rutaceae	Arnottianamide[59], avicine[68], benzo[c]phenanthridine[80], **chelerythrine[103]**, chelerythrine-psi-cyanide[105], des-N-methylchelerythrine[147], N-methylflindersine[290], oxyavicine[306], oxychelerythrine[307], toddalosin[394], toddasin[395]
Tribulus terrestris	Zygophyllaceae	**Gitogenin[190]**, harman[199]
Trichilia emetica	Meliaceae	**Chlorogenic acid[106]**, rohituka 3[345], trichilin A[392]
*Vernonia amygdalina**	Asteraceae	**Chlorogenic acid[106]**, **1,5-dicaffeoylquinic acid[2]**, **luteolin[273]**, luteolin-4-rutinoside[274], **luteolin-7-glucoside[275]**, **luteolin-7-rutinoside[276]**, vernolide[409], vernomygdin,[410] vernoniosides A1[411], A3[412], B1[413], B2[414], B3[415]
*Vernonia colorata**	Asteraceae	Hydroxyvernolide[219], vernolide[409]
Withania somnifera	Solanaceae	**Withaferin A[425]**, withasomnine[426]
Xanthium strumerium	Asteraceae	Xanthinin[427]
Xylopia aethiopica	Annonaceae	Bisabolene[87], kauran-16-alpha-ol[251], 15-oxo-kaurenoic acid[6], xylopic acid[428]
Ziziphus mucronata	Rhamnaceae	Mucronine D[285], sanjoinine A[355]
**<u>Plants with traditional use but lack of scientific evidence</u>**		
Argemone ochroleuca	Papaveraceae	**Berberine[81]**, **protopine[325]**, **sanguinarine[354]**
*Bulbine natalensis**	Asphodelaceae	Knipholone254
Elytropappus rhinocerotis	Asteraceae	Rhinocerotinoic acid[344]
Fumaria officinalis	Papaveraceae	**Sanguinarine[354]**
Gelsemium sempervirens	Gelsemiaceae	Gelsemicine[187]
Lannea acida	Anacardiaceae	Tetrahydroxyflavanone[388]
Mondia whitei	Apocynaceae	5-Chloropropacin[23], 7-hydroxy-4,6-dimethoxypropacin[25], propacin[323]
Rumex lanceolatus	Polygalaceae	Chrysophonein[108], **chrysophonol[109]**
Solanum tuberosum	Solanaceae	Solanine[366]
Strychnos spinosa	Loganiaceae	10-Hydroxyakagerine[3], strychnine[380]
Voacanga africana	Apocynaceae	Ibogaine[225], ibogamine[226], iboxygaine[227], vinburnine[416], voacamine[420], voacangine[421], voacorine[422], voaphylline[423], vobtusine[424]
Xysmalobium undulatum	Apocynaceae	Allouzarin[40], alloxysmalorin[41], uzarigenin[403], uzarin[404]

* These plants were noted in the source books African Herbal Pharmacopoeia (Brendler et al., 2010) and Medicinal Plants of South Africa (Van Wyk, 1997) as having anti-diabetic properties. The numbers 1-430 serves as the identification of each compound in Figure 1. All compounds in bold are compounds identified with some previous literature on their potential anti-diabetic activity.

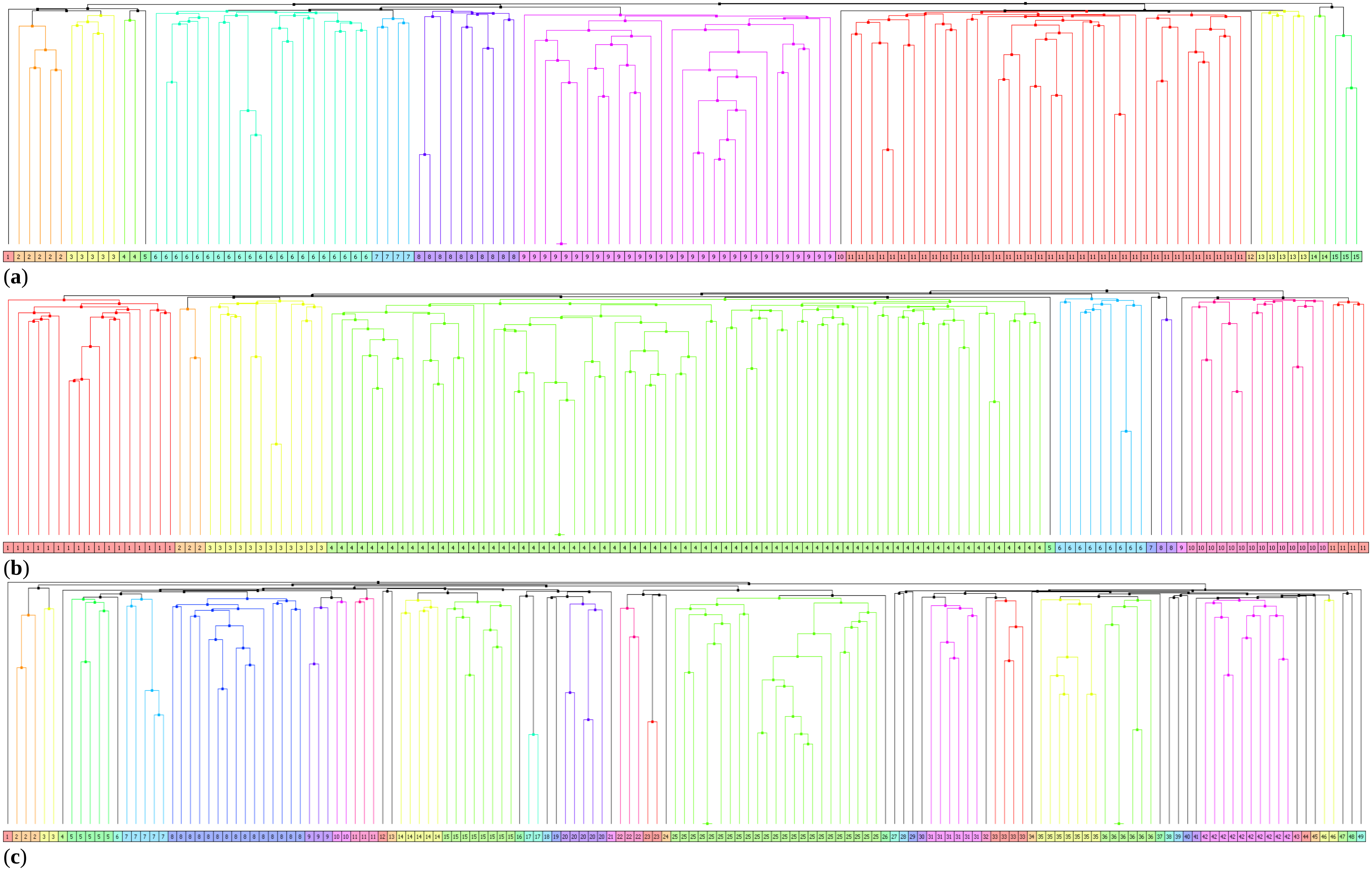
(a)
(b)
(c)

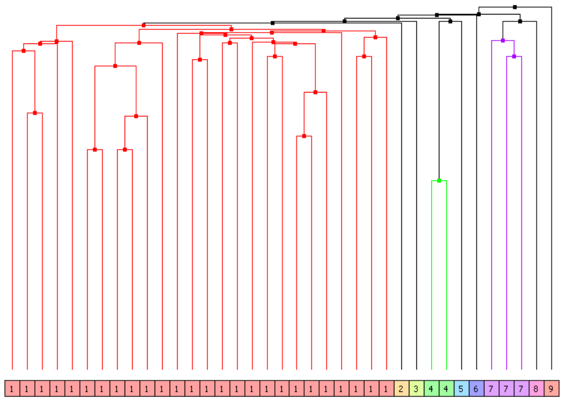

(d)

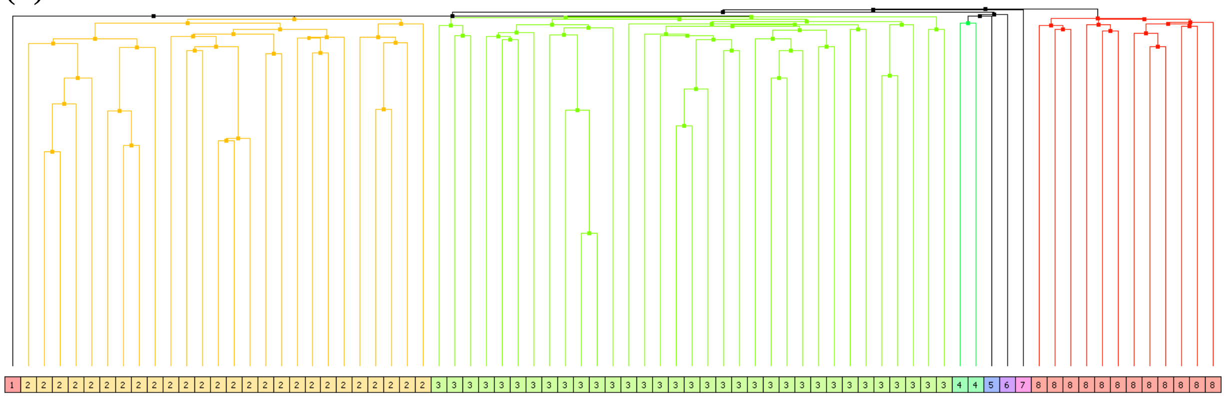

(e)

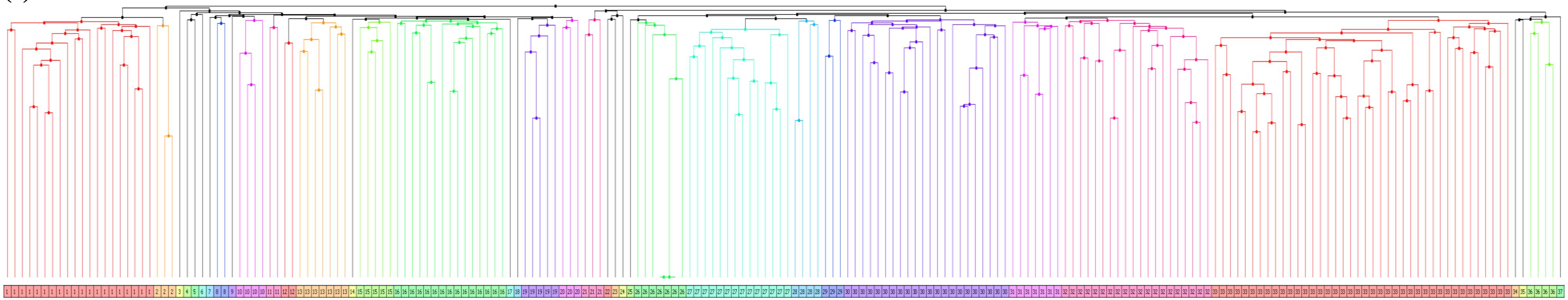

(f)

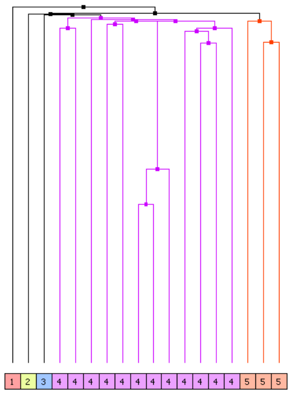

(g)

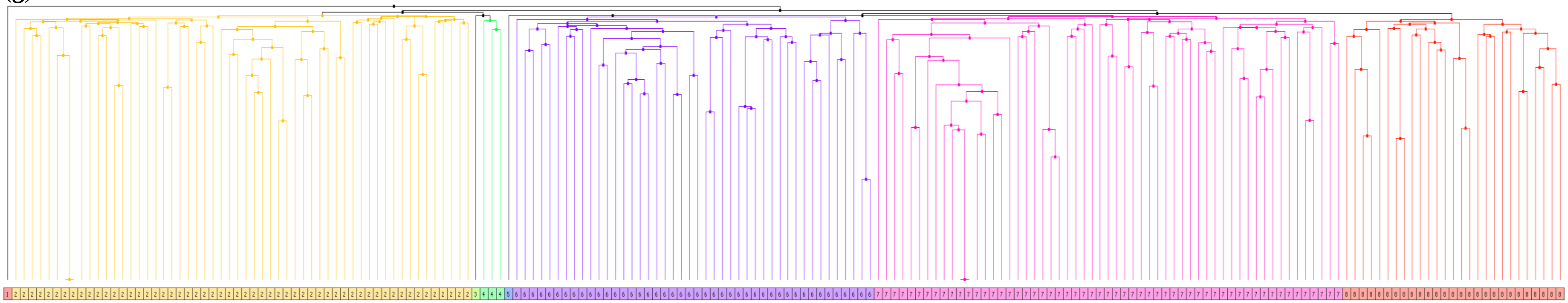

(h)

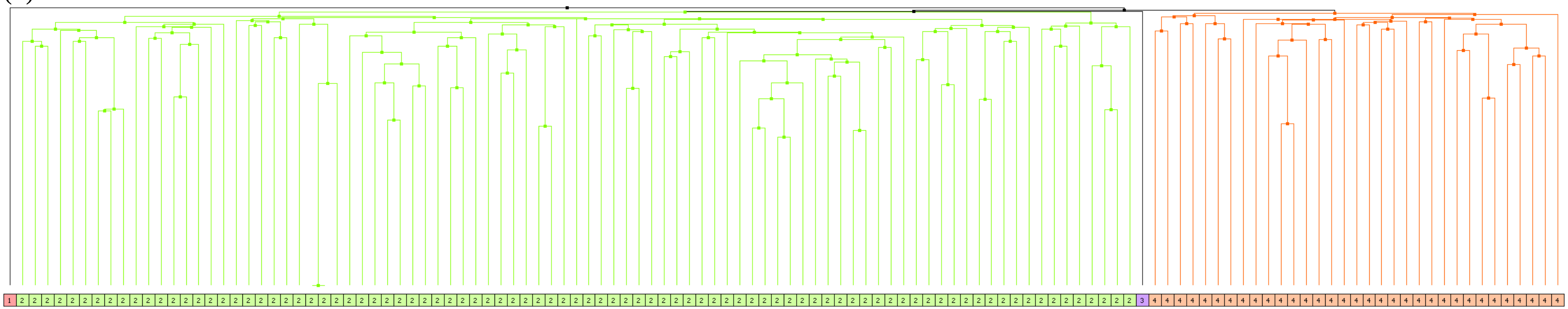

(i)

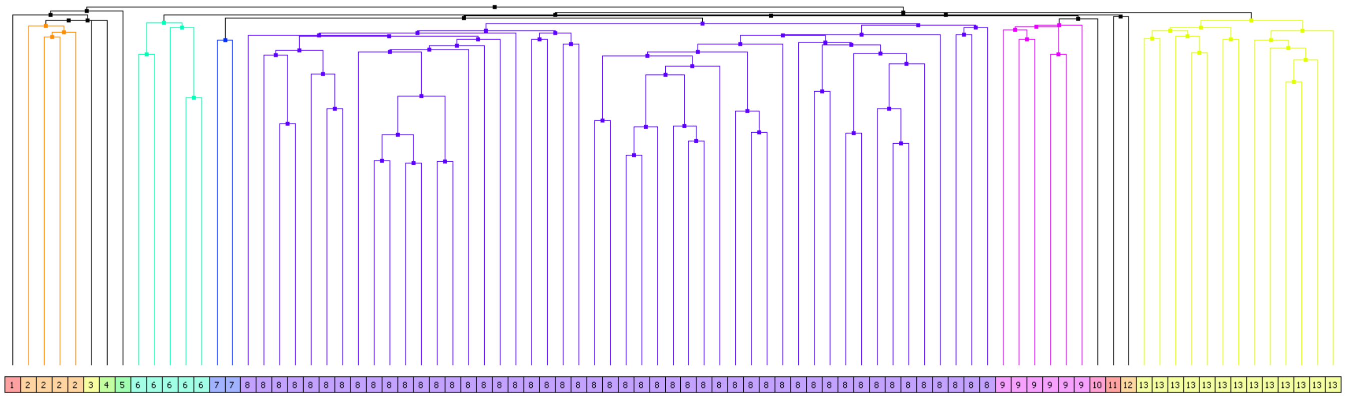

(j)

Figure S2. Dendrograms of hierarchical clustering analysis for (**a**) AKR1B1; (**b**) AMY2A; (**c**) DPP4; (**d**) FFAR1; (**e**) GCK; (**f**) HSD11B1; (**g**) MGAM; (**h**) PPARD; (**i**) PPARG; (**j**) RBP4. No clustering of compounds was found for six of the protein target-compound groups namely INSR, NR5A2, PDK2, PPARA, PTPN9, PYGL and RXRA.
